# The Effect of Vicinal Difluorination on the Conformation and Potency of Histone Deacetylase Inhibitors

**DOI:** 10.3390/molecules26133974

**Published:** 2021-06-29

**Authors:** A. Daryl Ariawan, Flora Mansour, Nicole Richardson, Mohan Bhadbhade, Junming Ho, Luke Hunter

**Affiliations:** 1 School of Chemistry, UNSW Sydney, Sydney, NSW 2052, Australia; daryl.ariawan@mq.edu.au (A.D.A.); flora_marlin26@hotmail.com (F.M.); nicole.richardson@unsw.edu.au (N.R.); junming.ho@unsw.edu.au (J.H.); 2 Mark Wainwright Analytical Centre, UNSW Sydney, Sydney, NSW 2052, Australia; m.bhadbhade@unsw.edu.au

**Keywords:** SAHA, Scriptaid, stereoselective fluorination, HDAC

## Abstract

Histone deacetylase enzymes (HDACs) are potential targets for the treatment of cancer and other diseases, but it is challenging to design isoform-selective agents. In this work, we created new analogs of two established but non-selective HDAC inhibitors. We decorated the central linker chains of the molecules with specifically positioned fluorine atoms in order to control the molecular conformations. The fluorinated analogs were screened against a panel of 11 HDAC isoforms, and minor differences in isoform selectivity patterns were observed.

## 1. Introduction

**Figure 1:**
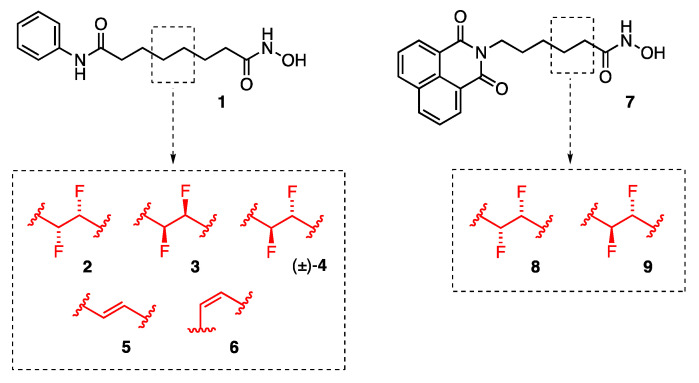
The known HDAC inhibitors SAHA (**1**) and Scriptaid (**7**), and the novel targets of this work (i.e., **2**–**6**, **8**, **9**).

Histone deacetylases (HDACs) are important enzymes whose functions include the regulation of gene expression [1]. In the nucleus, DNA is wound around histone proteins to create chromatin. Histones are post-translationally modified to contain acetyl groups on the ε-amino groups of some surface lysine residues; HDACs can catalyze the removal of these acetyl groups, thereby altering how tightly DNA winds around the histone, which in turn affects gene expression. Aberrant HDAC function is associated with a variety of diseases, including cancer [2], and this has prompted widespread interest in these enzymes as drug targets. Two well-known HDAC inhibitors are SAHA (also known as Vorinostat, **1**, Figure 1) [3] and Scriptaid (**7**) [4]. These inhibitors share three key features: an aromatic capping group that binds at the surface of the enzyme; a linker chain that penetrates through a tunnel leading into the interior of the enzyme; and a zinc-binding group that coordinates to a metal cofactor in the enzyme’s deeply buried active site.

A major issue in developing HDAC inhibitor drugs is the challenge of achieving isoform selectivity [5]. There are 18 different HDAC isoforms, comprising class I (HDAC1–3, HDAC8), class IIa (HDAC4–5, HDAC7, HDAC9), class IIb (HDAC6, HDAC10), class III (sirtuins 1–7), and class IV (HDAC11). The catalytic domains of HDAC classes I, II and IV are structurally very similar to one another. Compounds **1** and **7** are both pan-inhibitors, meaning that they are not selective for any one HDAC isoform. Although SAHA (**1**) has been approved for the treatment of cutaneous T-cell lymphoma, its administration causes side-effects such as nausea, anemia, and other metabolic issues [5]. There is hope that more isoform-selective HDAC inhibitors might cause fewer side-effects and/or be applicable to treat other diseases.

Significant effort has been invested into identifying the structural features that can increase isoform selectivity in HDAC inhibitors [6]. For example, the zinc-binding group (i.e., the hydroxamic acid moiety of **1** and **7**) can take many alternative forms, e.g., thiol, ketone, epoxide, oxadiazole or aminoanilide; the bulkier of these zinc-binding groups can confer selectivity for HDAC isoforms that have larger binding cavities proximal to the metal. Likewise, the capping group (i.e., the aromatic moieties of **1** and **7**) provides a wide scope for structural variation, with known HDAC inhibitors containing, e.g., cyclic peptides, linear peptides, indoles, cage hydrocarbons and many other structural motifs at this position. In contrast, the linker moiety (i.e., the alkyl chain of **1** and **7**) seems to offer a more limited opportunity for structural variation. Some HDAC isoforms (e.g., HDAC6, HDAC8, HDAC10) have wider substrate-binding tunnels that can be selectively targeted by bulkier linkers with, e.g., embedded aryl moieties [7]. However, HDAC isoforms that have narrow tunnels (e.g., HDAC1–3) are challenging to target through variation of the linker [8] because slim linkers usually fit not just the narrow-tunneled HDACs but also the wider ones.

A potential strategy for constraining the conformation of the linker chain without dramatically increasing the steric bulk is to attach fluorine atoms. In vicinal difluoroalkanes, conformations in which the F–C–C–F dihedral angle is *gauche* (±60°) are stabilized by hyperconjugative interactions (e.g., σ_CH_→σ*_CF_ or σ_CC_→σ*_CF_) [9]. According to this rationale, different stereochemical configurations of the 1,2-difluoroalkane motif should give different preferred conformations: *threo*-difluoroalkanes should favor the extended zigzag conformation, while *erythro*-difluoroalkanes should induce a “bend” into the alkyl chain (but also see ref. [10]). This phenomenon has been successfully exploited to modulate the properties of several classes of biologically relevant compounds, including lipids [11,12], neurotransmitters [13], cyclic peptides [14,15], alkaloids [16], sugars [17] and amino acids [18]. In the current work, we hypothesized that vicinal difluorination could provide a means of biasing the conformation of the linker chains of scaffolds **1** and **7** in ways that might confer selectivity for HDAC isoforms that have narrow binding tunnels (e.g., HDAC1–3).

Herein, we describe the synthesis, conformational analysis and HDAC inhibitory activity of three difluorinated analogs of SAHA (i.e., **2**–**4**, Figure 1). Two alkene analogs of SAHA (i.e., **5**–**6**, Figure 1) are also investigated in order to test the correspondence of *E*- and *Z*-alkenes with *threo*- and *erythro*-difluoroalkanes, respectively. We also describe a preliminary investigation of the Scriptaid scaffold, with two difluorinated analogs targeted (i.e., **8**–**9**).

## 2. Results and Discussion

### 2.1. Synthesis

**Scheme 1:**
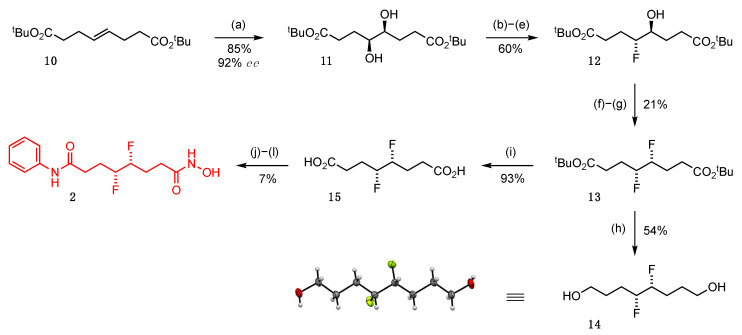
Synthesis of *threo*-difluorinated SAHA analog **2**. Reagents and conditions: (a) K_2_OsO_4_, (DHQ)_2_PHAL, K_3_Fe(CN)_6_, K_2_CO_3_, CH_3_SO_2_NH_2_, ^t^BuOH, H_2_O, 0 °C; (b) SOCl_2_, pyridine, CH_2_Cl_2_, 0 °C; (c) NaIO_4_, RuCl_3_, H_2_O, CH_2_Cl_2_, CH_3_CN, 0 °C; (d) TBAF, THF, CH_3_CN; (e) *p*-TsOH, dioxane; (f) (CF_3_SO_2_)_2_O, pyridine, CH_2_Cl_2_, 0 °C; (g) TBAF, THF, CH_2_Cl_2_, 0 °C; (h) LiAlH_4_, THF; (i) CF_3_CO_2_H; (j) DIC, HOBt, aniline, DMF; (k) ClCO_2_Et, Et_3_N, CH_3_OH, THF; (l) NH_2_OH.

The *threo*-difluorinated SAHA analog **2** was synthesized according to the method outlined in Scheme 1. The known *E*-alkene **10** [19] underwent a Sharpless asymmetric dihydroxylation reaction to deliver the diol **11** in good yield and with reasonable enantiopurity. Diol **11** was then converted into the fluorohydrin **12** through a four-step sequence proceeding via a cyclic sulfate intermediate [20]. The alcohol group of **12** was then activated as the triflate and displaced with fluoride [20], giving the vicinal difluoroalkane **13** in modest yield along with a suspected lactone-containing side product. The *threo*-stereochemistry of difluoroalkane **13** was proven by converting a portion of this material into the diol **14**, whose structure was unambiguously determined by X-ray crystallography (Scheme 1) [21]. The remaining quantity of diester **13** was then deprotected by treatment with trifluoroacetic acid to give diacid **15**. The latter compound was converted into the target compound **2** through a three-step sequence. First, a statistical coupling reaction between diacid **15** and aniline was performed [22], giving a non-symmetrical acid/amide as the major product. The acid moiety of this intermediate was then converted into a mixed anhydride [23], which finally upon treatment with hydroxylamine gave the hydroxamic acid derivative **2** in low overall yield.

In order to obtain the enantiomeric *threo*-difluorinated SAHA analog **3** (Figure 1), a variation of the sequence shown in Scheme 1 was performed whereby the initial asymmetric dihydroxylation step was performed using the pseudoenantiomeric cinchona alkaloid catalyst (see Supplementary Materials).

**Scheme 2:**
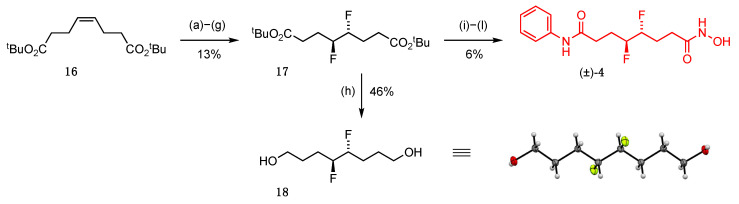
Synthesis of *erythro*-difluorinated SAHA analog (±)-**4**. Reagents and conditions: (a) AD-mix α, CH_3_SO_2_NH_2_, ^t^BuOH, H_2_O, (CH_3_)_2_CO, 0 °C; (b) SOCl_2_, pyridine, CH_2_Cl_2_, 0 °C; (c) NaIO_4_, RuCl_3_, H_2_O, CH_2_Cl_2_, CH_3_CN, 0 °C; (d) TBAF, THF, CH_3_CN; (e) *p*-TsOH, dioxane; (f) (CF_3_SO_2_)_2_O, pyridine, CH_2_Cl_2_, 0 °C; (g) TBAF, THF, CH_2_Cl_2_, 0 °C; (h) LiAlH_4_, THF; (i) CF_3_CO_2_H; (j) DIC, HOBt, aniline, DMF; (k) ClCO_2_Et, Et_3_N, CH_3_OH, THF; (l) NH_2_OH.

The *erythro*-difluorinated SAHA analog (±)-**4** was synthesized as shown in Scheme 2. The known *Z*-alkene **16** [24] underwent *cis*-dihydroxylation, followed by stepwise fluorinations by analogy with the methods already described in Scheme 1, to afford the *meso*-difluoroalkane **17** (Scheme 2). The stereochemistry of compound **17** was proven by converting a portion of this material into the crystalline derivative **18** (Scheme 2 [21]). Finally, the remaining stock of compound **17** was converted into the racemic *erythro*-difluorinated SAHA analog (±)-**4** using the same methods as those described in Scheme 1.

The syntheses of the *E*- and *Z*-alkene analogs of SAHA (i.e., compounds **5**–**6**, Figure 1) are described in the Supplementary Materials.

**Scheme 3:**
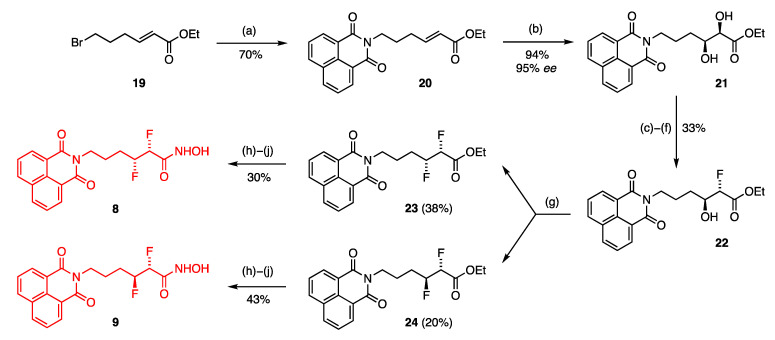
Synthesis of fluorinated Scriptaid analogs **8**–**9**. Reagents and conditions: (a) naphthalimide, K_2_CO_3_, DMF, 50 °C; (b) AD-mix α, CH_3_SO_2_NH_2_, ^t^BuOH, H_2_O, 0 °C; (c) SOCl_2_, pyridine, CH_2_Cl_2_; (d) NaIO_4_, RuCl_3_, H_2_O, CH_2_Cl_2_, CH_3_CN, 0 °C; (e) TBAF, THF, CH_3_CN; (f) H_2_SO_4_, H_2_O, THF; (g) DeoxoFluor, 80 °C; (h) HCl, dioxane, 101 °C; (i) ClCO_2_Et, *N*-methylmorpholine, THF, 0 °C; (j) NH_2_OH.HCl.

With the targeted SAHA analogs in hand, attention was turned to the Scriptaid series. Only two fluorinated Scriptaid analogs (i.e., **8**–**9**, Figure 1) were targeted in this preliminary investigation. The α,β-locations of the fluorine atoms in **8**–**9** were originally envisaged as an opportunity to investigate Jacobsen’s one-step difluorination methodology [25]; however, the divergent stepwise fluorination route shown in Scheme 3 ultimately became the preferred method. To commence the synthesis, the known alkyl bromide **19** [26] underwent a Gabriel-type reaction to deliver the naphthalimide-containing compound **20**. Asymmetric dihydroxylation then furnished the diol **21** in high yield and with 95% *ee*. Cyclic sulfate formation, followed by ring-opening with TBAF, delivered the fluorohydrin **22** as a single regioisomer. Fluorohydrin **22** was then treated with DeoxoFluor with the intention of converting it into the *threo*-difluorinated product **23** (Scheme 3). Compound **23** was indeed obtained by these means, but unexpectedly the *erythro*-difluorinated product **24** was also formed in significant quantity. The formation of **24** might be explained by invoking anchimeric assistance of the naphthalimide moiety [27]. The diastereoisomeric compounds **23** and **24** were readily separable by flash chromatography. Finally, esters **23** and **24** were hydrolyzed under acidic conditions to the corresponding acids, and converted via the mixed anhydride into the target hydroxamic acid derivatives **8**–**9** (Scheme 3).

### 2.2. Conformational Analysis

The influences of fluorination upon the conformations of **2**–**4** and **8**–**9** were examined through a DFT study. Given the number of rotatable bonds within these molecules, it was not feasible to fully explore the conformational space. Instead, a starting structure was created in which the alkyl chain was fully extended, then only certain key dihedral angles (i.e., F–C–C–F for **2**–**4**, F–C–C–F and F–C–C=O for **8**–**9**) were rotated through 120° increments, and finally the various conformers obtained were optimized at the M06-2X level of theory with the 6-31G* basis set, using SMD implicit solvation in methanol [28]. The resulting relative Gibbs free energies are displayed in Table 1. In order to validate the DFT results, NMR coupling constants were predicted for the low-energy conformers using the GIAO method at the B3LYP/6-311+G(d,p) level of theory [13]. The predicted J-values were averaged according to a Boltzmann distribution, and then compared with experimental J-values derived from ^1^H and ^19^F NMR spectra (Table 1).

For the *threo*-difluorinated SAHA analog **2**, the extended zigzag conformation (i.e., **2a**, Table 1) was identified as having the lowest energy. The “bent” conformation in which the C–F bonds are again *gauche* (i.e., **2b**, Table 1) was found to be slightly higher in energy. The remaining conformation (**2c**), in which the C–F bonds are aligned *anti*, was found to have a considerably higher energy and was therefore excluded from further analysis. Assuming no significant population of higher-energy conformers, conformers **2a** and **2b** should be present in a 58:42 molar ratio at 298 K. This population distribution gives a reasonable match with experimental J-values (Table 1), but the predicted ^3^J_HF_ values are somewhat lower than the experimental values. This suggests that the major conformer (**2a**) might actually be even more dominant in solution. The **2a**:**2b** ratio was adjusted by trial-and-error, and 80:20 was found to give a better match between calculated and experimental J-values (not shown). Regardless, the major conformer of the *threo*-difluorinated SAHA analog **2** is the extended one, as expected.

The enantiomeric *threo*-difluorinated SAHA analog **3** was not investigated computationally because its conformers are the mirror-image of those of **2** and the energies are the same. Notably, the lowest-energy (i.e., extended) conformations of **2** and **3** would be superimposable except for the orientations of the C–F bonds, so analogs **2** and **3** might be expected to share similar biological properties.

For the *erythro*-difluorinated SAHA analog **4**, one enantiomer was arbitrarily selected for computational investigation (Table 1). All three staggered rotamers about F–C–C–F were found to have very similar energies: the *g*^+^ and *g*^−^ conformers (i.e., **4a** and **4b**, respectively) were isoenergetic, and the *anti* conformer (i.e., **4c**) was only slightly higher in energy. This translates into a predicted population distribution of 37.5:37.5:25. Comparison of J-values (Table 1) once again showed a reasonable match between theory and experiment, although adjusting the ratio of **4a**:**4b**:**4c** by trial-and-error (not shown) revealed that a 46:46:8 ratio seemed to give a closer match between calculated and experimental J-values. Regardless, the major conformers of the *erythro*-difluorinated SAHA analog **4** are the “bent” ones, as expected. Notably, the predicted *g*^+^/*g*^−^ disorder of **4** means that its two enantiomers would be expected to share similar backbone conformations [14].

For the *threo*-difluorinated Scriptaid analog **8**, an extended conformation (**8a**) was identified as having the lowest energy (Table 1). In this conformation, the F–C–C–F dihedral angle is *gauche* and the F–C–C=O angle is close to 180°, as expected [29]. There is a substantial gap in energy between conformation **8a** and the next higher-energy conformation (**8b**); in the latter conformation, the F–C–C=O dihedral angle remains at 180° while the alkyl chain bends to give a F–C–C–F dihedral angle that is *g*^−^ rather than *g*^+^. Slightly higher again is conformation **8c**, in which both the F–C–C–F and F–C–C=O dihedral angles are close to 180°. Unfortunately, it was not possible to validate these findings by comparing calculated vs. experimental NMR J-values because the NMR spectra of **8** were complex and had multiple overlapping signals. Nevertheless, the correspondence between theory and experiment that was observed with the other compounds in the series (Table 1) provides some reassurance that this conformational analysis of **8** is reliable, i.e., that analog **8** strongly prefers an extended alkyl chain.

For the *erythro*-difluorinated Scriptaid analog **9**, two low-energy conformers were identified in which the F–C–C–F dihedral angle is alternately *g*^−^ and *g*^+^ (**9a** and **9b**, respectively, Table 1). Both of these low-energy conformations have a “bent” carbon chain. In the next higher-energy conformation (**9c**), the F–C–C–F dihedral angle is *anti*; this conformation is considerably higher in energy and was therefore excluded from further analysis. Assuming no contribution from any higher-energy conformations, conformations **9a** and **9b** should be present in a 60:40 molar ratio. This ratio gives a good match between calculated and experimental NMR J-values (Table 1), confirming that the *erythro*-difluorinated analog **9** has an overall “bent” shape.

### 2.3. HDAC Inhibition

**Figure 2:**
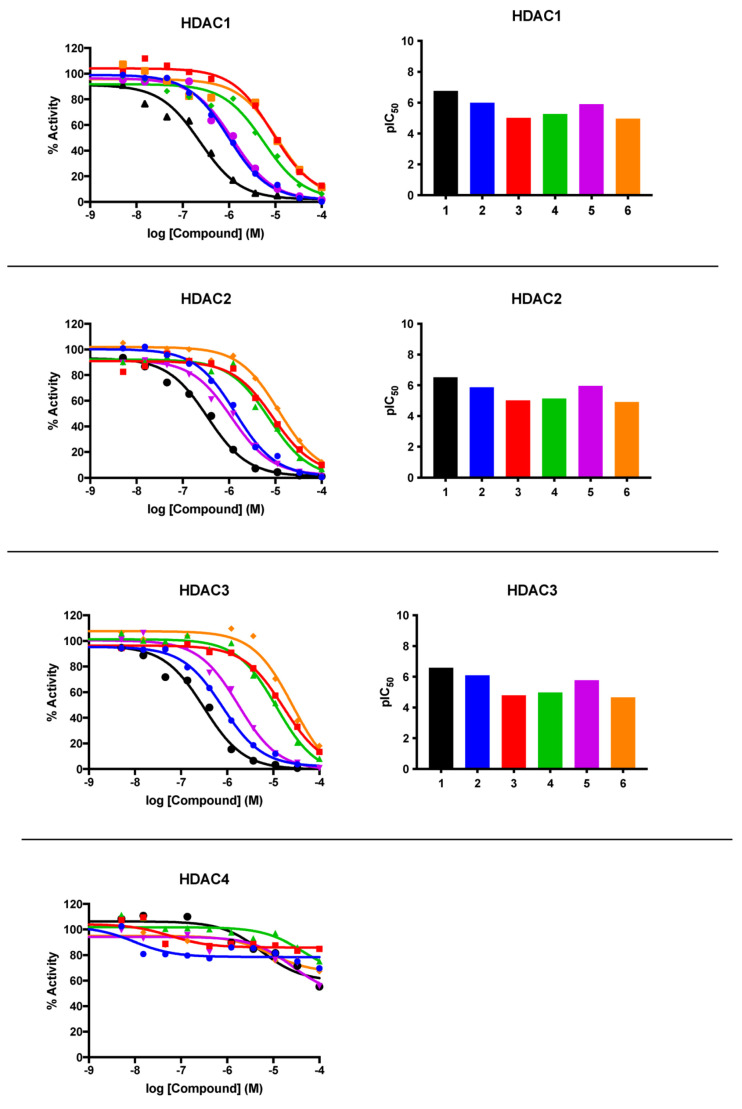

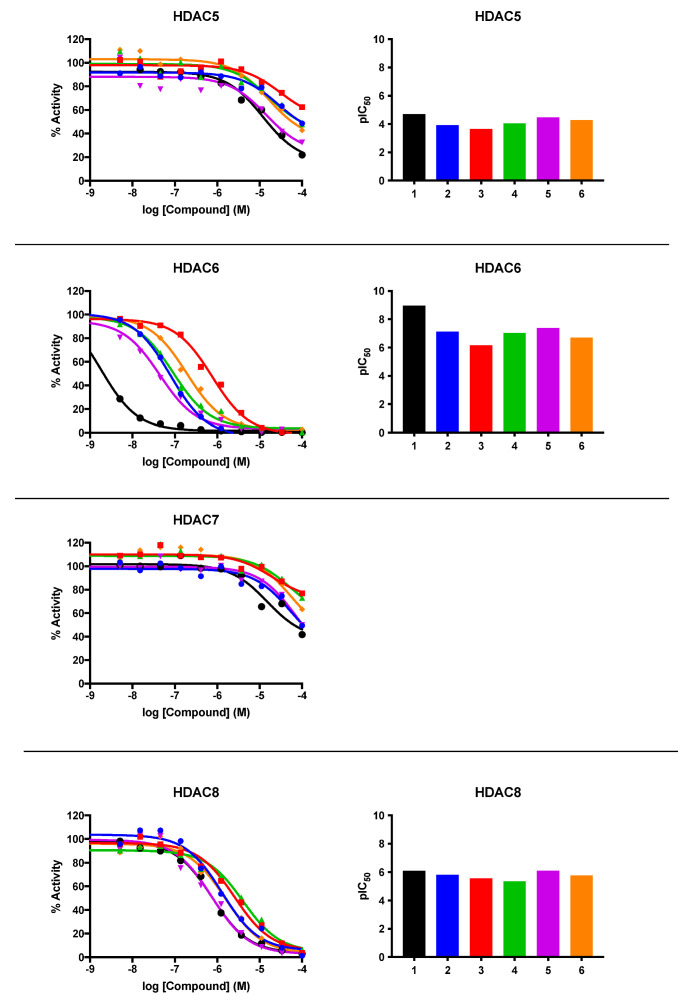

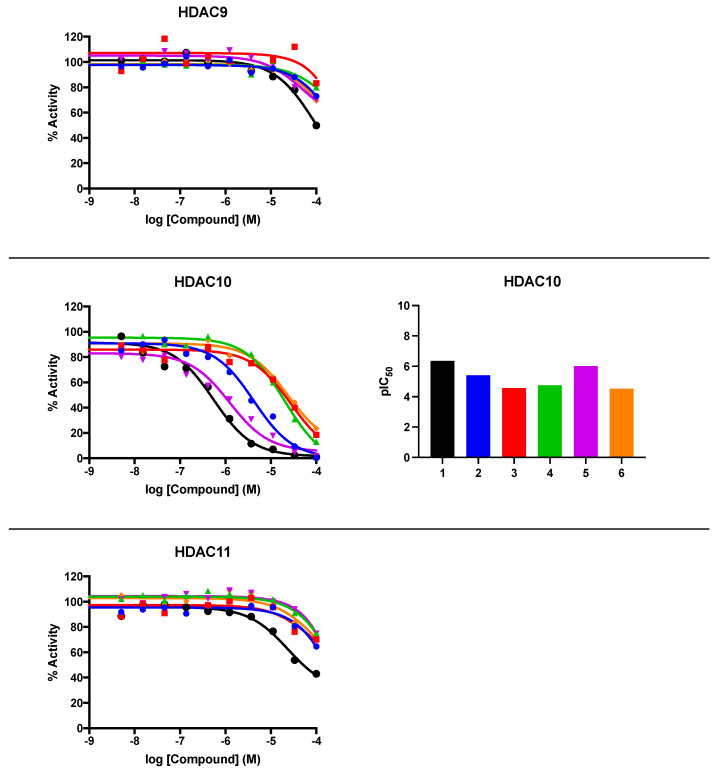
HDAC inhibitory activity of SAHA analogs. Black = **1**; blue = **2**; red = **3**; green = (±)-**4**; magenta = **5**; orange = **6**. Histograms of pIC_50_ values (right column) are provided only where the inhibition is significant.

**Figure 3:**
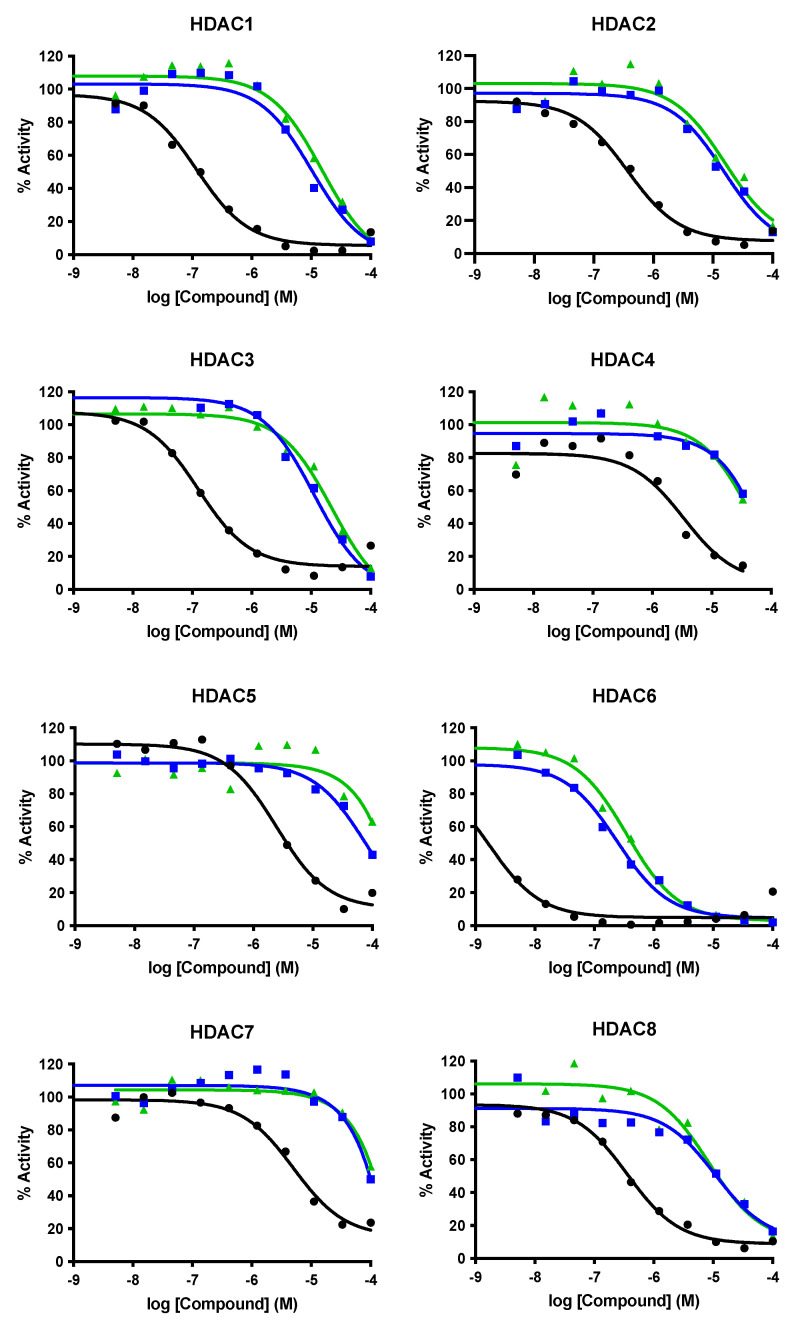

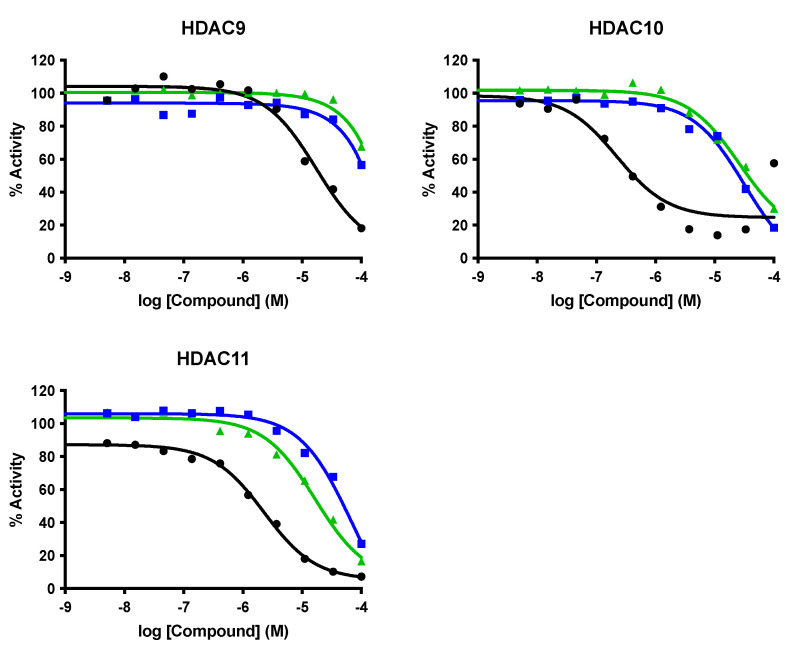
HDAC inhibitory activity of Scriptaid analogs. Black = **7**; blue = **8**; green = **9**.

Compounds **1**–**9** were investigated in inhibition assays targeting 11 HDAC isoforms (Figure 2 and Figure 3; also see Supplementary Materials for the data in tabulated form).

The SAHA-series compounds are considered first (i.e., **1**–**6**, Figure 2). These compounds all tend to be more active towards HDAC classes I and IIb (i.e., HDAC1,2,3,6,8,10) and less active towards HDAC classes IIa and IV (i.e., HDAC4,5,7,9,11). The lead compound (**1**) was the most potent of the series towards all HDAC isoforms tested, particularly the HDAC6 isoform.

Although there were no improvements in potency due to fluorination or alkene incorporation, there are nevertheless some interesting comparisons to be made within the set of new analogs **2**–**6**.

Towards the class I and IIb HDACs (i.e., HDAC1,2,3,6,8,10), the *threo*-difluorinated analog **2** and the *E*-alkene analog **5** are consistently more potent than the *erythro*-difluorinated analog **4** and the *Z*-alkene analog **6**, respectively. This suggests that an extended geometry is optimal for binding to all of these HDAC isoforms, and that the *threo*- and *erythro*-difluoroalkane motifs are effective mimics of *E*- and *Z*-alkenes, respectively. This feeds into a broader discussion about the influence of vicinal difluorination on the conformations of longer carbon chains [10]. 

The *threo*-difluorinated analog **2** is consistently more potent than its enantiomer (**3**). This result is intriguing because, at first glance, the two enantiomers have broadly similar preferred geometries (i.e., an extended zigzag carbon chain) and might therefore have been expected to have similar activity. To probe this issue in more detail, docking studies were undertaken and the results are presented in the next section.

Within the SAHA series (i.e., compounds **1**–**6**), fluorination or alkene incorporation does not alter the HDAC isoform selectivity in a dramatic fashion; for example, all compounds in the series are most active towards HDAC6, like SAHA. Nevertheless, some subtle changes in isoform selectivity can be seen. For example, the lead compound (**1**) is 5-fold selective for HDAC3 over HDAC8, whereas in the analogs **3**–**6** the direction of selectivity between these two isoforms is reversed (see Supplementary Materials for a side-by-side comparison). Thus, fluorination can subtly alter the isoform selectivity within a single HDAC class (I). 

The Scriptaid-series compounds (i.e., compounds **7**–**9**, Figure 3) were considered next. The lead compound (**7**) is a broader-spectrum inhibitor than SAHA (**1**): compound **7** shows some activity even towards the HDAC isoforms that were not inhibited by the SAHA-series compounds (i.e., HDAC4,5,7,9,11). However, fluorination was generally detrimental to potency within the Scriptaid series, albeit less so towards the HDAC6 isoform, and little difference between the two fluorinated compounds **8** and **9** was observed (Figure 3). This suggests that the bioactive conformations are precluded in analogs **8**–**9**, an issue which is explored in the next section.

### 2.4. Docking Studies

Docking studies were performed in an attempt to rationalize some of the observed trends in HDAC inhibition by compounds **1**–**9**. HDAC2 was selected as the template for this study because of its importance as a target for breast cancer drug development [30], and because it features a narrow substrate-binding tunnel that we had hoped to target through our linker modifications. A published crystal structure [22] of SAHA (**1**) bound to HDAC2 formed the basis for our docking study, which we performed using Discovery Studio/GOLD.

**Figure 4:**
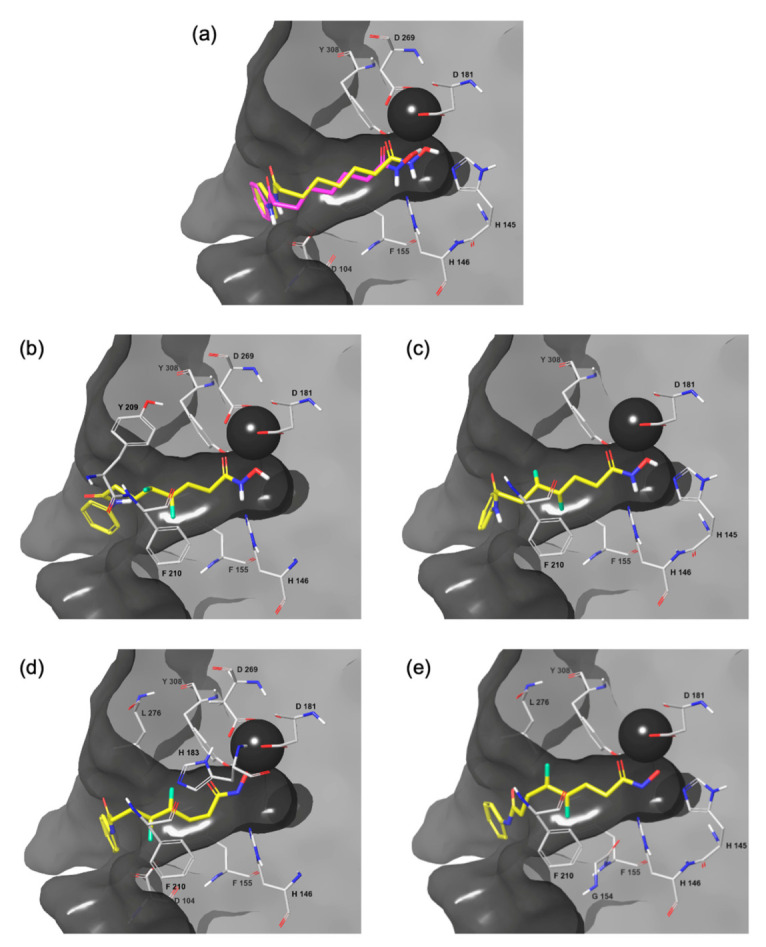
Predicted binding poses of SAHA analogs to HDAC2. Dark grey sphere = zinc ion. (**a**) Ligand **1** (magenta = crystal structure [21]; yellow = docked); (**b**) ligand **2**; (**c**) ligand **3**; (**d**) ligand (4*R*,5*S*)-**4**; (**e**) ligand (4*S*,5*R*)-**4**.

The SAHA series of compounds (**1**–**6**) was considered first. Re-docking of the lead compound (**1**, Figure 4a) generated a binding pose that quite closely resembled the pose from the crystal structure, providing confidence that the docking protocol was reliable. The bound pose of **1** features an extended zigzag conformation in the central portion of the linker moiety. Next, the fluorinated analogs **2**–**4** were docked according to the same protocol (Figure 4b–e; note that the two enantiomers of **4** were docked separately; all docking scores are provided in the Supplementary Materials). For each fluorinated inhibitor, the bound pose maintains an extended zigzag conformation in the central portion of the linker moiety. This could explain why the *threo*-difluorinated analog **2** is a more potent HDAC2 inhibitor than the *erythro*-difluorinated analog **4**: for analog **2** the required binding conformation is already highly populated in solution, whereas for analog **4** the required binding conformation is only a small fraction of the population in solution.

Comparing the docking results for the enantiomeric *threo*-difluorinated SAHA analogs **2** and **3** may help to explain the surprisingly low potency of **3**. Although both **2** and **3** favor the same extended conformation of the linker moiety, the bound poses do not precisely overlay with one another (Figure 4b,c). For analog **3**, the bound pose is somewhat rotated and translated compared with **2**, such that analog **3** coordinates the zinc only in a monodentate fashion and also makes fewer favorable contacts at the entrance to the binding tunnel. It may be that unfavorable steric and/or electronic clashes between the fluorines and the residues lining the tunnel are preventing analog **3** from adopting the same pose as that of analog **2**. 

**Figure 5:**
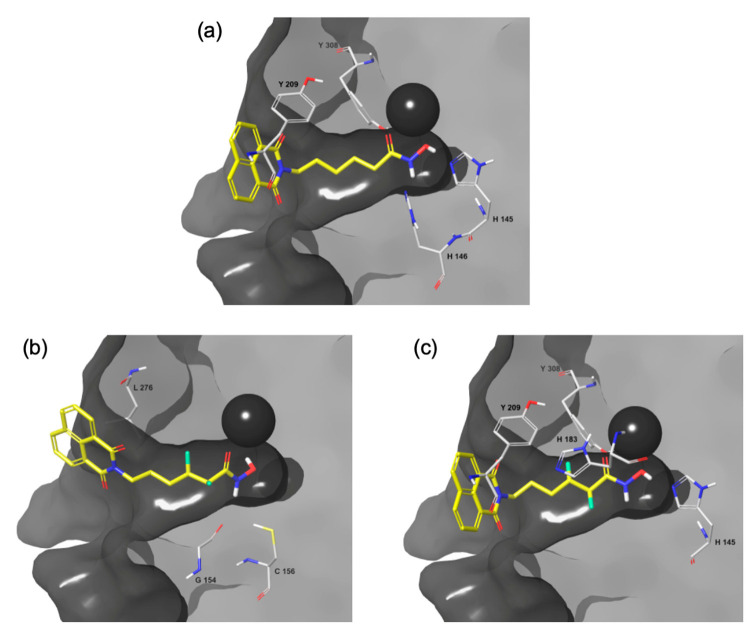
Predicted binding poses of Scriptaid analogs to HDAC2. (**a**) Ligand **7**; (**b**) ligand **8**; (**c**) ligand **9**.

The Scriptaid series of compounds (**7**–**9**) was considered next. For the lead compound (**7**, Figure 5a), the docked pose features bidentate chelation of the metal and an extended conformation of the linker moiety in the portion neighboring the hydroxamic acid moiety (a pose which appears to differ slightly from that seen in a previous docking study [31]). The extended linker geometry is maintained in the docked poses of both fluorinated Scriptaid analogs (**8**–**9**, Figure 5b,c). Notably, however, the bound conformations of **8** and **9** do not resemble any of the low-energy conformations that were identified in the DFT study (Table 1). For analog **8**, the F–C–C=O dihedral angle in the docked pose is far from the ideal value of 180°, suggesting that the docked pose is a high-energy conformation that would explain the poor inhibitory activity of **8**. For analog **9**, neither the F–C–C–F dihedral angle nor the F–C–C=O dihedral angle matches the preferred conformation, once again explaining the poor inhibitory activity.

It is intriguing to consider the enantiomer of **8** (not shown), which was not synthesized in this preliminary study. The lowest-energy conformation of *ent*-**8** can be inferred from Table 1; its carbon chain would closely resemble the bound pose of **8** (Figure 5b). This suggests that *ent*-**8** might be an interesting candidate for future study.

## 3. Conclusions

A series of new HDAC inhibitors based on SAHA (**1**) and Scriptaid (**7**) have been synthesized, in which the central linker portion of each analog is decorated with stereospecifically positioned fluorine atoms. Conformational analysis by DFT and NMR reveals that the *threo*-difluorinated analogs (i.e., **2**, **3**, **8**) favor an extended geometry in solution, while the *erythro*-difluorinated analogs (i.e., **4**, **9**) favor a “bent” carbon chain with *g*^+^/*g*^−^ disorder. Screening of the seven new compounds against HDAC1–11 revealed that fluorination is generally detrimental to inhibitory potency but that it can cause subtle variations in selectivity between isoforms within class I HDACs. Overall, this work highlights that achieving selectivity for HDAC isoforms that have narrow substrate binding tunnels (e.g., HDAC1–3) through modifications to the linker remains a challenging task.

## Figures and Tables

**Table 1:** Conformational analysis of **2**–**4** and **8**–**9**.

Chemical Structure	Conformations	Calculated (Black) vs. Experimental (Grey) NMR Coupling Constants
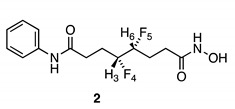	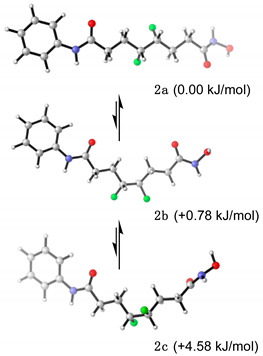	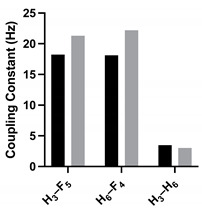
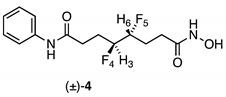	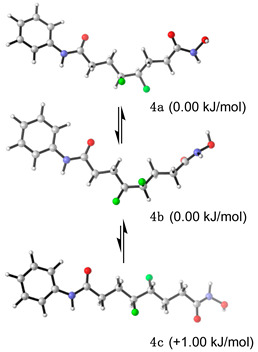	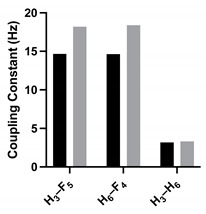
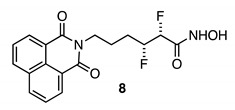	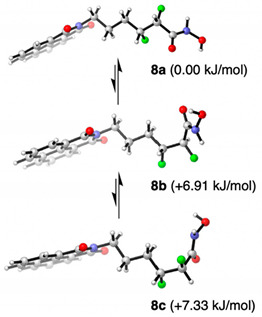	n/a *
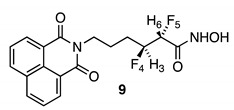	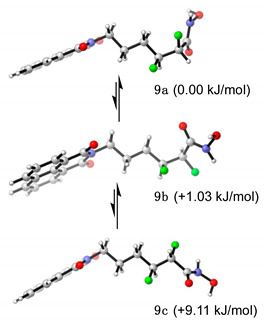	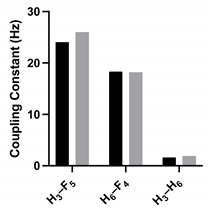

* Overlapping signals in experimental NMR spectra.

## Data Availability

No applicable.

## Supplementary Materials

Synthetic procedures; measurement of optical purity of compounds **12** and **21**; NMR spectra of novel compounds; solid-state structures of compounds **14** and **18**; solution-state conformations of compounds **14** and **18**; HDAC inhibition assays; docking studies.

## Sample Availability

Samples of the compounds are not available from the authors.
