# Supplementary material for: The Effect of Vicinal Difluorination on the Conformation and Potency of Histone Deacetylase Inhibitors
Source: Molecules. 2021 Jun 29;26(13):3974. doi: 10.3390/molecules26133974 (PMC8271401; doi:10.3390/molecules26133974)
Supplement: Supplementary file 1 [file molecules-26-03974-s001.zip › molecules-1257879-supplementary.pdf]

The effect of vicinal difluorination on the conformation and potency of histone deacetylase inhibitors

*A. Daryl Ariawan, Flora Mansour, Nicole Richardson, Mohan Bhadbhade, Junming Ho and Luke Hunter**

SUPPORTING INFORMATION

Contents:

1. Reagents and instrumentation …………………………………………… S-2
2. Synthetic procedures ……………………………………………………. S-3
3. Measurement of optical purity of compounds **12** and **21** ……………. S-24
4. NMR spectra of novel compounds …………………………………… S-28
5. Solid-state structures of compounds **14** and **18** ………………………. S-69
6. Solution-state conformations of compounds **14** and **18** …………….. S-71
7. HDAC inhibition assays ……………………………………………… S-76
8. Docking studies ………………………………………………………. S-78
9. References ……………………………………………………………. S-80

1. Reagents and instrumentation

All reactions were performed in oven-dried glassware under the protection of a nitrogen atmosphere unless otherwise stated. All reagents and solvents were purchased in the highest available quality and used as received. Reactions were monitored by thin layer chromatography (TLC) using Merck aluminium backed silica gel 60 F_{254} plates (0.2 mm), and visualised under short-wave UV light (254 nm) in combination with potassium permanganate stain. Purification by flash chromatography was carried out using Davisil 40−63 mesh silica gel; eluting solvents are stated as volume/volume mixtures. Nuclear Magnetic Resonance (NMR) spectra were recorded using a Bruker Avance III 300, 400 or 600 MHz spectrometer at 298 K unless otherwise stated; chemical shifts are reported in parts per million downshift from tetramethylsilane. Most resonances were assigned unambiguously by inspection of the coupling constants and chemical shift; otherwise, two-dimensional (COSY, HSQC, HMBC) experiments were used to assign the resonances. The numbering system used for NMR assignments is indicated on each chemical structure drawing. Melting points were determined using a Stanford Research Systems OptiMelt automated melting point apparatus. Infrared spectra were recorded using a Cary 360 Fourier Transform Infrared (FTIR) spectrometer equipped with attenuated total reflectance (ATR) with diamond crystal inset. Optical rotations were measured using a Perkin Elmer model 341 polarimeter (λ = 589 nm, l = 1 dm) and concentrations are reported in grams per 100 mL. High Resolution Mass Spectrometry (HRMS) was recorded at the Bioanalytical Mass Spectrometry Facility (BMSF) at UNSW using an Orbitrap LTQ XL ion trap MS in positive or negative ion mode with an electrospray (ESI) ion source.

2. Synthetic procedures

(4*R*,5*R*)-4,5-Difluoro-*N*[1]-hydroxy-*N*[8]-phenyloctanediamide (2)

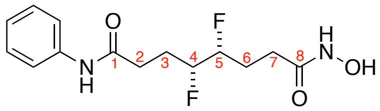

Preparation of hydroxylamine solution: A mixture of $NH_2OH.HCl$ (130 mg, 1.87 mmol), KOH (105 mg, 1.87 mmol) and MeOH (2 mL) was stirred for 15 min at 0 °C. The formed precipitate was filtered off, and the filtrate was used for the coupling reaction.

Coupling reaction: A mixture of acid **25** (12 mg, 0.042 mmol), ethylchloroformate (0.0060 mL, 0.063 mmol), *N*-methylmorpholine (0.0070 mL, 0.063 mmol), THF (1.5 mL) and MeOH (0.5 mL) was stirred at 0 °C for 15 min. The precipitate was filtered off, and freshly prepared hydroxylamine solution (0.2 mL) was added to the filtrate. The resulting mixture was stirred at r.t. for 30 min. The mixture was concentrated under a stream of N_2, and the residue was subjected to preparative reverse-phase HPLC employing 0.1% formic acid/H_2O as eluent A and 0.1% formic acid/MeCN as eluent B (gradient = 98% A for 2 min, then ramped to 100% B over 40 min, then kept at 100% B for 6 min) to afford the title compound as a white solid (3.5 mg, 29%); **m.p.** 172.5–173.1 °C; $[\alpha]_D$ +22.9 (*c* 0.18, MeOH); **IR** (neat) ν_{max} (cm^{-1}) 2360, 1659, 1541, 1443, 1063; **^{1}H NMR** (600 MHz, MeOD) δ 7.56 (d, 2H, *J* = 8.1 Hz, ArH), 7.32 (t, 2H, *J* = 7.7 Hz, ArH), 7.10 (t, 1H, *J* = 7.7 Hz, ArH), 4.66–4.48 (m, 2H, H4 and H5), 2.59 (m, 2H, H7), 2.30 (m, 2H, H2), 2.19–1.85 (m, 4H, H3 and H6); **^{13}C{^{1}H} NMR** (150 MHz, MeOD) δ 171.8 (C1), 170.4 (C8), 138.4 (ArC), 128.4 (ArC), 123.8 (ArC), 119.9 (ArC), 92.4 (dd, *J* = 177.0, 19.8 Hz, C4), 92.3 (dd, *J* = 173.5, 16.8 Hz, C5), 31.5 (d, *J* = 4.0 Hz, C7), 27.7 (d, *J* = 3.1 Hz, C2), 26.3 (dd, *J* = 18.9, 6.0 Hz, C6), 26.2 (dd, *J* = 15.5, 3.0 Hz, C3); **^{19}F NMR** (376 MHz, MeOD) δ –200.8 (m, 2F); **^{19}F{^{1}H} NMR** (376 MHz, MeOD) δ –200.7 (d, 1F, *J* = 9.8 Hz), –200.8 (d, 1F, *J* = 9.8 Hz); **HRMS** (ESI, +ve): $C_{14}H_{18}F_2N_2O_3Na^+$ [MNa^+] requires *m/z* 323.1178, found 323.1230.

(4*S*,5*S*)-4,5-Difluoro-*N*[1]-hydroxy-*N*[8]-phenyloctanediamide (3)

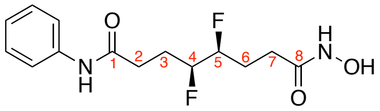

Preparation of hydroxylamine solution: A mixture of $NH_2OH.HCl$ (0.12 g, 1.7 mmol), KOH (97 mg, 1.7 mmol) and MeOH (2 mL) was stirred for 15 min at 0 °C. The formed precipitate was filtered off, and the filtrate was used for the coupling reaction.

Coupling reaction: A mixture of *ent*-**25** (12 mg, 0.042 mmol), ethylchloroformate (0.0060 mL, 0.063 mmol), *N*-methylmorpholine (0.0070 mL, 0.063 mmol), THF (1.5 mL) and MeOH (0.5 mL) was stirred at 0 °C for 15 min. The precipitate was filtered off, and freshly prepared hydroxylamine solution (0.2 mL) was added to the filtrate. The resulting mixture was stirred at r.t. for 30 min. The mixture was concentrated under a stream of N_2, and the residue was subjected to preparative reverse-phase HPLC employing 0.1% formic acid/H_2O as eluent A and 0.1% formic acid/MeCN as eluent B (gradient = 98% A for 2 min, then ramped to 100% B over 40 min, then kept at 100% B for 6 min) to afford the title compound as a white solid (5.0 mg, 40%); **m.p.** 184.5–184.9 °C; **$[\alpha]_D$** –22.5 (*c* 0.45, MeOH); **IR** (neat) ν_{max} (cm^{-1}) 2405, 1650, 1602, 1423, 1106, 893; **^{1}H NMR** (600 MHz, MeOD) δ 7.57 (d, 2H, *J* = 8.7 Hz, ArH), 7.33 (t, 2H, *J* = 7.6 Hz, ArH), 7.10 (t, 1H, *J* = 7.4 Hz, ArH), 4.70–4.44 (m, 2H, H4 and H5), 2.57 (m, 2H, H7), 2.30 (m, 2H, H2), 2.19–1.91 (m, 4H, H3 and H6); **^{13}C{^{1}H} NMR** (150 MHz, MeOD) δ 171.9 (C1), 170.4 (C8), 138.4 (ArC), 128.4 (ArC), 123.8 (ArC), 119.9 (ArC), 92.4 (dd, *J* = 177.2, 20.3 Hz, C4), 92.3 (dd, *J* = 177.5, 20.5 Hz, C5), 31.5 (d, *J* = 4.2 Hz, C7), 27.7 (d, *J* = 3.9 Hz, C2), 26.4 (dd, *J* = 11.3, 5.8 Hz, C6), 26.2 (dd, *J* = 11.1, 6.0 Hz, C3); **^{19}F NMR** (376 MHz, MeOD) δ –200.8 (m, 2F); **^{19}F{^{1}H} NMR** (376 MHz, MeOD) δ –200.7 (d, 1F, *J* = 9.9 Hz), –200.8 (d, 1F, *J* = 9.9 Hz); **HRMS** (ESI, +ve): $C_{14}H_{18}F_2N_2O_3Na^+$ [MNa^+] requires *m/z* 323.1178, found 323.1171.

rac-(4*R*,5*S*)-4,5-Difluoro-*N*^{1}-hydroxy-*N*^{8}-phenyloctanediamide (±-4)

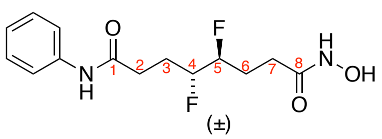

Preparation of hydroxylamine solution: A mixture of $NH_2OH.HCl$ (114 mg, 1.64 mmol), KOH (92.0 mg, 1.64 mmol) and MeOH (2 mL) was stirred for 15 min at 0 °C. The formed precipitate was filtered off, and the filtrate was used for the coupling reaction.

Coupling reaction: A mixture of acid (±)-**29** (12 mg, 0.042 mmol), ethylchloroformate (0.0060 mL, 0.063 mmol), *N*-methylmorpholine (0.0070 mL, 0.063 mmol), THF (1.5 mL) and MeOH (0.5 mL) was stirred at 0 °C for 15 min. The precipitate was filtered off, and freshly prepared hydroxylamine solution (0.2 mL) was added to the filtrate. The resulting mixture was stirred at r.t. for 30 min. The

mixture was concentrated under a stream of N_2, and the residue was subjected to preparative reverse-phase HPLC employing 0.1% formic acid/H_2O as eluent A and 0.1% formic acid/MeCN as eluent B (gradient = 98% A for 2 min, then ramped to 100% B over 40 min, then kept at 100% B for 6 min) to afford the title compound as a white solid (2.3 mg, 18%); **m.p.** 153.3–155.1 °C; **IR** (neat) ν_{max} (cm^{-1}) 3260, 2352, 1657, 1613, 1554, 1394, 1030; **^{1}H NMR** (600 MHz, MeOD) δ 7.56 (d, 2H, *J* = 7.9 Hz, ArH), 7.32 (t, 2H, *J* = 7.6 Hz, ArH), 7.10 (t, 1H, *J* = 7.4 Hz, ArH), 4.75–4.51 (m, 2H, H4 and H5), 2.60 (m, 2H, H7), 2.31 (m, 2H, H2), 2.18–1.93 (m, 4H, H3 and H6); **^{13}C{^{1}H} NMR** (150 MHz, MeOD) δ 171.8 (C1), 170.4 (C8), 138.4 (ArC), 128.4 (ArC), 123.8 (ArC), 119.9 (ArC), 92.8 (dd, *J* = 174.6, 17.7 Hz, C4), 92.6 (dd, *J* = 174.6, 16.1 Hz, C5), 31.6 (d, *J* = 3.0 Hz, C7), 27.7 (d, *J* = 3.0 Hz, C2), 25.7 (dd, *J* = 17.3, 5.7 Hz, C6), 25.6 (dd, *J* = 18.1, 5.9 Hz, C3); **^{19}F NMR** (376 MHz, MeOD) δ –195.8 (m, 2F); **^{19}F{^{1}H} NMR** (376 MHz, MeOD) δ – 195.7 (d, 1F, *J* = 13.6 Hz), –195.9 (d, 1F, *J* = 13.6 Hz); **HRMS** (ESI, +ve): $C_{14}H_{18}F_2N_2O_3Na^+$ [MNa^+] requires *m/z* 323.1178, found 323.1175.

(*E*)-*N*^{1}-hydroxy-*N*^{8}-phenyloct-4-enediamide (5)

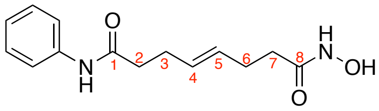

Preparation of hydroxylamine solution: A mixture of $NH_2OH.HCl$ (121 mg, 1.75 mmol), KOH (98.0 mg, 1.75 mmol) and MeOH (2 mL) was stirred for 15 min at 0 °C. The formed precipitate was filtered off, and the filtrate was used for the coupling reaction.

Coupling reaction: A mixture of acid **31** (11 mg, 0.042 mmol), ethylchloroformate (0.0064 mL, 0.067 mmol), *N*-methylmorpholine (0.0074 mL, 0.067 mmol), THF (1.5 mL) and MeOH (0.5 mL) was stirred at 0 °C for 15 min. The precipitate was filtered off, and freshly prepared hydroxylamine solution (0.4 mL) was added to the filtrate. The resulting mixture was stirred at r.t. for 30 min. The mixture was concentrated under a stream of N_2, and the residue was subjected to preparative reverse-phase HPLC employing 0.1% formic acid/H_2O as eluent A and 0.1% formic acid/MeCN as eluent B (gradient = 98% A for 2 min, then ramped to 100% B over 40 min, then kept at 100% B for 6 min) to afford the title compound as a white solid (5.1 mg, 38%); **m.p.** 173.7–174.1 °C; **IR** (neat) ν_{max} (cm^{-1}) 3290, 2407, 1647, 1619, 1423, 967, 752; **^{1}H NMR** (600 MHz, MeOD) δ 7.55 (d, 2H, *J* = 8.7 Hz, ArH), 7.31 (t, 2H, *J* = 7.5 Hz, ArH), 7.10 (t, 1H, *J* = 7.4 Hz, ArH), 5.56 (m, 2H, H4 and H5), 2.43 (m, 2H, H2), 2.36 (m, 2H, H3), 2.31 (m, 2H, H6), 2.13 (t, 2H, *J* = 7.8 Hz, H7); **^{13}C{^{1}H} NMR** (150 MHz, MeOD) δ 172.5 (C1), 170.9 (C8), 138.4 (ArC), 129.7 (C4), 129.2 (C5), 128.4 (ArC),

123.7 (ArC), 119.9 (ArC), 36.5 (C2), 32.4 (C7), 28.4 (C3), 28.2 (C6); **HRMS** (ESI, +ve): $C_{14}H_{18}N_2O_3Na^+$ [MNa^+] requires *m/z* 285.1210, found 285.1205.

(*Z*)-*N*^{1}-hydroxy-*N*^{8}-phenyloct-4-enediamide (6)

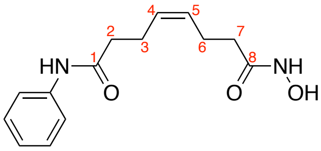

Preparation of hydroxylamine solution: A mixture of $NH_2OH.HCl$ (256 mg, 3.69 mmol), KOH (207 mg, 3.69 mmol) and MeOH (3 mL) was stirred for 15 min at 0 °C. The formed precipitate was filtered off, and the filtrate was used for the coupling reaction.

Coupling reaction: A mixture of acid **33** (27.0 mg, 0.109 mmol), ethylchloroformate (0.0158 mL, 0.164 mmol), *N*-methylmorpholine (0.0176 mL, 0.164 mmol), THF (3.0 mL) and MeOH (1.0 mL) was stirred at 0 °C for 15 min. The precipitate was filtered off, and freshly prepared hydroxylamine solution (0.9 mL) was added to the filtrate. The resulting mixture was stirred at r.t. for 30 min. The mixture was concentrated under a stream of N_2, and the residue was subjected to preparative reverse-phase HPLC employing 0.1% formic acid/H_2O as eluent A and 0.1% formic acid/MeCN as eluent B (gradient = 98% A for 2 min, then ramped to 100% B over 40 min, then kept at 100% B for 6 min) to afford the title compound as a clear oil (14.0 mg, 49%); **IR** (neat) ν_{max} (cm^{-1}) 3209, 1640, 1498, 1419, 1310, 1062; **^{1}H NMR** (400 MHz, MeOD) δ 7.55 (d, 2H, *J* = 7.9 Hz, ArH), 7.31 (t, 2H, *J* = 7.7 Hz, ArH), 7.10 (t, 1H, *J* = 7.5 Hz, ArH), 5.47 (m, 2H, H4 and H5), 2.50–2.38 (m, 6H, H2, H3, and H6), 2.15 (t, *J* = 7.3 Hz, 2H, H7); **^{13}C{^{1}H} NMR** (150 MHz, MeOD) δ 172.5 (C1), 170.9 (C8), 138.4 (ArC), 129.1 (C4), 128.5 (C5), 128.4 (ArC), 123.8 (ArC), 119.9 (ArC), 36.5 (C2), 32.3 (C7), 23.2 (C3), 23.0 (C6); **HRMS** (ESI, +ve): $C_{14}H_{18}N_2O_3Na^+$ [MNa^+] requires *m/z* 285.1210, found 285.1214.

(2*R*,3*R*)-6-(1,3-Dioxo-1*H*-benzo[*de*]isoquinolin-2(3*H*)-yl)-2,3-difluoro-*N*-hydroxyhexanamide (8)

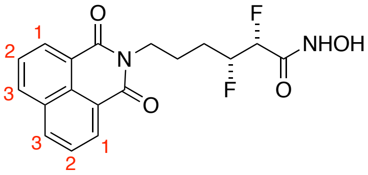

To a solution of ester **23** (12.0 mg, 0.032 mmol) in dioxane (470 μL) was added concentrated HCl (470 μL) and the resultant mixture was heated at reflux overnight. The mixture was concentrated under a flow of nitrogen and dried under vacuum. The resultant residue was dissolved in THF (100 μL) and NMM (3.5 μL, 0.034 mmol) and ethyl chloroformate (3.4 μL, 0.032 mmol) was added at 0 °C and stirred for 2 h. The resultant mixture was filtered and the filtrate was added to a solution of hydroxylamine hydrochloride (2.2 mg, 0.032 mmol) in methanol (10 μL), and was stirred overnight. The mixture was concentrated and HPLC purification (MilliQ/formic acid (0.1%):MeCN/formic acid (0.1%) 0 to 100%) afforded the title compound as a white solid (3.3 mg, 30%); **mp** >200 °C; **[α]D** 28.8 (*c* = 1.00, DCM); **IR** (neat) ν_{max} (cm^{-1}) 3408, 1655, 1049, 1022; **^{1}H NMR** (600 MHz, DMSO-d_6) δ 11.12 (br s, 1H, NHOH), 8.99 (br s, 1H, NH), 8.52 (m, 2H, *J* = 7.2 Hz, H1), 8.48 (d, *J* = 8.3 Hz, 2H, H3), 7.89 (dd, *J* = 8.3, 7.9 Hz, 2H, H2), 5.10–4.98 (m, 2H, CHFCHF), 4.08 (t, *J* = 7.2 Hz, 2H, NCH_2), 1.88–1.65 (m, 4H, $NCH_2CH_2CH_2$); **^{13}C{^{1}H} NMR** (75 MHz, DMSO-d_6) δ 164.4 (2 x C=O) 134.2, 131.6 131.5, 128.1, 127.0, 122.4 (ArC), 91.9 (dd, *J* = 179.2, 20.8 Hz), 88.4 (dd, *J* =189.3, 23.2 Hz), 39.7, 26.7 (dd, *J* = 21.9, 5.7 Hz), 23.7 (d, *J* = 3.3 Hz); **^{19}F NMR** (282 MHz, $CDCl_3$) δ –193.1 (m, 1F), –192.7 (m, 1F); **HRMS** (ESI,+ve) $C_{18}H_{16}F_2N_2O_4H^+$ [$M+H^+$] requires *m/z* 363.1104, found 363.1108.

(2*R*,3*S*)-6-(1,3-Dioxo-1*H*-benzo[*de*]isoquinolin-2(3*H*)-yl)-2,3-difluoro-*N*-hydroxyhexanamide (9)

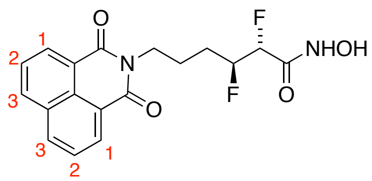

To a solution of ester **24** (12 mg, 0.032 mmol) in dioxane (470 μL) was added concentrated aqueous HCl (470 μL) and the resultant mixture was heated at reflux overnight. The mixture was concentrated under a stream of nitrogen and dried under vacuum. The resultant residue was dissolved in THF (100 μL) and NMM (3.5 μl, 0.034 mmol) and ethyl chloroformate (3.35 μL, 0.032 mmol) were added at 0 °C and stirred for 2 h. The resultant mixture was filtered and the filtrate was added to a solution of hydroxylamine hydrochloride (2.2 mg, 0.032 mmol) in methanol (10 μL), and was stirred overnight. The mixture was concentrated and HPLC (MilliQ/formic acid (0.1%):MeCN/formic acid (0.1%) 0–100%) purification afforded the title compound as a white solid (4.7 mg, 43%); **m.p.** >200 °C; **[α]D** 29.5 (*c* = 1.00, MeOH); **IR** (neat) ν_{max} (cm^{-1}) 3408, 1656, 1049, 1023; **^{1}H NMR** (600 MHz, DMSO-d_6) δ 8.51 (d, *J* = 7.1 Hz, 2H, H1), 8.47 (d, *J* = 8.0 Hz 2H, H3), 7.86 (dd, *J* = 8.0,

7.5 Hz, 2H, H2), 4.89 (ddd, J = 47.0, 27.0, 2.1 Hz, 1H, CHFCH$_2$), 4.60 (ddd, J = 47.5, 28.8, 2.1 Hz, CHFCO), 4.08 (t, J = 9.4 Hz, 2H, NCH$_2$); **^{13}C{^{1}H} NMR** (75 MHz, DMSO-d$_6$) to 160.4, 158.6, 137.0, 134.0 133.5, 130.1, 129.9, 124.7 (ArC), 87.4 (dd, J = 178.8, 22.5 Hz), 87.3 (dd, J = 177.5, 18.4 Hz), 34.8, 22.8 (dd, J = 21.9, 4.3 Hz), 18.9 (dd, J = 7.0, 2.5 Hz); **^{19}F NMR** (282 MHz, DMSO-d$_6$) δ –84.6 (m, 1F, CHFCO), –194.2 (m, 1F,CHFCHFCO); **HRMS** (ESI, +ve) $C_{18}H_{17}F_2N_2O_4^+$ [M+H$^+$] requires *m/z* 363.1104, found 363.1106.

Di-*tert*-butyl (*E*)-oct-4-enedioate (10)[1]

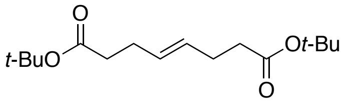

To a stirred solution of diisopropylamine (4.6 mL, 33 mmol) in THF (40 mL) at –78 °C was added *n*-butyllithium (1.6 M solution in hexanes, 21 mL, 33 mmol) dropwise. The mixture was stirred for 15 min before *t*-butyl acetate (4.4 mL, 33 mmol) was added dropwise. The solution was stirred at –78 ºC for 30 min. A solution of (*E*)-1,4-dibromobutene (2.3 g, 11 mmol) in THF (10 mL) was added dropwise at –78 ºC. The mixture was stirred at –78 ºC for 5 h. Saturated aqueous NH_4Cl (50 mL) was added, and the mixture was extracted with ethyl acetate (3 × 60 mL). The combined organic extracts were dried with $MgSO_4$ and concentrated. The residue was purified by flash chromatography (9:1 hexane:EtOAc) to afford the title compound as white solid (2.2 g, 78%); **m.p.** 52–54 °C; **^{1}H NMR** (300 MHz, CDCl$_3$) δ 5.33 (br s, 2H, 2×CH), 2.12 (s, 8H, 4×CH$_2$), 1.31 (s, 18H, 6×CH$_3$); **^{13}C{^{1}H} NMR** (75 MHz, CDCl$_3$) δ 172.1, 129.2, 79.8, 35.1, 27.9, 27.8; spectroscopic data in accordance with literature values.[1]

Di-*tert*-butyl (4*S*,5*S*)-4,5-dihydroxyoctanedioate (11)

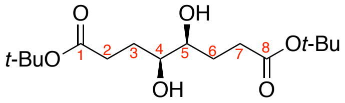

To a solution of $K_2OsO_4.2H_2O$ (22.0 mg, 0.0597 mmol), (DHQ)$_2$PHAL (93.0 mg, 0.119 mmol), $K_3(Fe(CN)_6)$ (11.6 g, 35.3 mmol), K_2CO_3 (4.88 g, 35.3 mmol), and $CH_2SO_2NH_2$ (1.12 g, 11.8 mmol) in *t*-BuOH (80 mL) and H_2O (80 mL) at 0 °C was added alkene **10** (3.35 g, 11.8 mmol). The mixture was stirred at 0 °C overnight. Saturated aqueous Na_2SO_3 solution (90 mL) was added, and the mixture was stirred for 30 min at r.t.. The mixture was extracted with CH_2Cl_2 (3 × 120 mL). The organic

layers were combined, dried with $MgSO_4$, filtered, and concentrated under vacuum. The residue was purified by flash chromatography (2:1 hexane:EtOAc) to afford the title compound as a clear oil (3.20 g, 85%); **[α]D** –5.6 (*c* 1.79, $CHCl_3$); **IR** (neat) ν_{max} (cm^{-1}) 3413, 2974, 1723, 1365, 1251, 1144, 846; **^{1}H NMR** (400 MHz, $CDCl_3$) δ 3.41 (m, 2H, H4 and H5), 3.13 (s, 2H, 2×OH), 2.40 (td, *J* = 7.1, 2.9 Hz, 4H, H2 and H7), 1.76 (m, 4H, H3 and H6), 1.43 (s, 18H, 9×CH_3); **^{13}C{^{1}H} NMR** (100 MHz, $CDCl_3$) δ 173.8, 80.6, 73.7, 31.9, 28.5, 28.1; **HRMS** (ESI, +ve): $C_{16}H_{30}O_6Na^+$ [MNa^+] requires *m/z* 341.1935, found 341.1925.

Di-*tert*-butyl (4*R*,5*R*)-4,5-dihydroxyoctanedioate (*ent*-11)

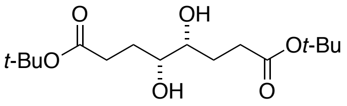

The title compound was obtained *via* the same procedure as that described for compound **11**, with the exception that $(DHQD)_2PHAL$ was used instead of $(DHQ)_2PHAL$. Data for *ent*-**11**: clear oil (3.19 g, 63%); **[α]D** +6.3 (*c* 2.84, MeOH); other spectroscopic data identical to those of compound **11**.

Di-*tert*-butyl (4*R*,5*S*)-4-fluoro-5-hydroxyoctanedioate (12)

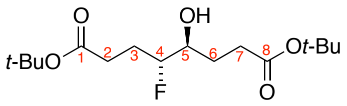

Thionyl chloride (0.51 mL, 7.0 mmol) was added to a solution of diol **11** (1.1 g, 3.5 mmol) and pyridine (0.85 mL, 11 mmol) in dry CH_2Cl_2 (15 mL) at 0 °C. The mixture was stirred for 30 min at 0 °C. Saturated aqueous $CuSO_4$ solution (15 mL) was added, and the mixture was extracted with CH_2Cl_2 (3 × 20 mL). The organic layers were combined, dried ($MgSO_4$), filtered, and concentrated to give an intermediate cyclic sulfite as clear oil. This residue was dissolved in acetonitrile (20 mL) and CH_2Cl_2 (20 mL) and cooled to 0 °C. $NaIO_4$ (1.5 g, 7.0 mmol), ruthenium chloride hydrate (~100 mg) and water (2 mL) were added, and the mixture was stirred at 0 °C for 3 h. Diethyl ether (50 mL) was added, and the mixture was washed with water (30 mL), saturated aqueous $NaHCO_3$ (30 mL) and brine (30 mL). The organic layer was dried ($MgSO_4$), filtered, and concentrated to give an intermediate cyclic sulfate as a clear oil. This residue was dissolved in MeCN (15 mL) and the solution was cooled to 0 °C. A solution of TBAF (1 M in THF, 7.00 mL, 7.00 mmol) was added, and the mixture was stirred at r.t. overnight. Brine (15 mL) was added and the mixture was extracted with

CH_2Cl_2 (3 × 25 mL). The combined organic layers were dried ($MgSO_4$), filtered, and concentrated to give a yellow oil. This residue was dissolved in dioxane (105 mL), and *p*-toluenesulfonic acid (996 mg, 5.24 mmol) was added. The mixture was stirred for 1.5 h at r.t. Saturated $NaHCO_3$ (100 mL) was added, and the mixture was extracted with CH_2Cl_2 (3 × 100 mL). The combined organic layers were dried ($MgSO_4$), filtered, and concentrated. The residue was purified by flash chromatography (4:1 hexane:EtOAc) to afford the title compound as clear oil (671 mg, 60%); **$[\alpha]_D$** +3.90 (*c* 1.59, $CHCl_3$); **IR** (neat) ν_{max} (cm^{-1}) 3445, 2976, 1725, 1366, 1252, 1149, 846; **^1H NMR** (400 MHz, $CDCl_3$) δ 4.36 (ddt, *J* = 48.2, 9.7, 4.9 Hz, 1H, H4), 3.65 (m, 1H, H5), 2.91 (s, 1H, OH), 2.40 (m, 4H, H2 and H7), 1.99 (m, 1H, H3a), 1.92 (m, 1H, H3b), 1.87 (m, 1H, H6a), 1.68 (m, 1H, H6b), 1.43 (s, 18H, 6×CH_3); **$^{13}C\{^1H\}$ NMR** (100 MHz, $CDCl_3$) δ 173.6 (C1), 172.7 (C8), 95.1 (d, *J* = 171.3 Hz, C4), 80.7 ($\underline{C}(CH_3)_3$), 80.6 ($\underline{C}(CH_3)_3$), 71.8 (d, *J* = 23.3 Hz, C5), 31.9, 30.7 (d, *J* = 3.7 Hz, C2), 28.0 (6×CH_3), 27.1 (d, *J* = 4.9 Hz, C6), 25.5 (d, *J* = 20.9 Hz, C3); **^{19}F NMR** (376 MHz, $CDCl_3$) δ –192.4 (m, 1F); **$^{19}F\{^1H\}$ NMR** (376 MHz, $CDCl_3$) δ –192.4 (s, 1F); **HRMS** (ESI, +ve): $C_{16}H_{29}FO_5Na^+$ [MNa^+] requires *m/z* 343.1891, found 343.1885.

Di-*tert*-butyl (4*S*,5*R*)-4-fluoro-5-hydroxyoctanedioate (*ent*-12)

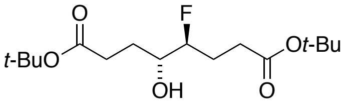

The title compound was obtained *via* the same procedure as that described for compound **12**. Data for *ent*-**12**: clear oil (0.67 g, 46%); **$[\alpha]_D$** –18.2 (*c* 3.28, $CHCl_3$); other spectroscopic data identical to those of compound **12**.

Di-*tert*-butyl (4*R*,5*R*)-4,5-difluorooctanedioate (13)

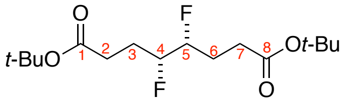

Trifluoromethanesulfonic anhydride (0.280 mL, 1.69 mmol) was added to a solution of fluorohydrin **12** (181 mg, 0.565 mmol) and pyridine (0.140 mL, 1.69 mmol) in CH_2Cl_2 (5 mL) at 0 °C. The resulting mixture was stirred at 0 °C for 5 min. The mixture was filtered and TBAF (1 M in THF, 5.65 mL, 5.65 mmol) was added to the filtrate at 0 °C. The mixture was stirred for 3 h at 0 °C. Brine (10 mL) was added and the mixture was extracted with CH_2Cl_2 (3 × 15 mL). The combined organic

layers were dried with $MgSO_4$, filtered, and concentrated. The residue was purified by flash chromatography (6:1 hexane:EtOAc) to afford the title compound as a clear oil (38.0 mg, 21%); **[α]D** +15.1 (*c* 5.30, $CHCl_3$); **IR** (neat) ν_{max} (cm^{-1}) 2976, 1725, 1366, 1252, 1149, 847; **^{1}H NMR** (400 MHz, $CDCl_3$) δ 4.51 (m, 2H, H4 and H5), 2.43 (m, 4H, H2 and H7), 1.98 (m, 4H, H3 and H6), 1.46 (s, 18H, 6×CH_3); **^{13}C{^{1}H} NMR** (100 MHz, $CDCl_3$) δ 172.0 (C1 and C8), 92.2 (dd, *J* = 177.4, 20.3 Hz, C4 and C5), 80.7 (C(CH_3)$_3$), 30.6 (t, *J* = 2.6 Hz, C2 and C7), 28.1 (6×CH_3), 26.0 (dd, *J* = 15.5, 11.7 Hz, C3 and C6); **^{19}F NMR** (376 MHz, $CDCl_3$) δ –199.2 (m, 2F); **^{19}F{^{1}H} NMR** (376 MHz, $CDCl_3$) δ –199.2 (s, 2F); **HRMS** (ESI, +ve) $C_{16}H_{28}F_2O_4Na^+$ [MNa^+] requires *m/z* 345.1848, found 345.1837.

(4*R*,5*R*)-4,5-Difluorooctane-1,8-diol (14)

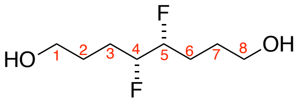

Lithium aluminium hydride (1 M in THF, 3.13 mL, 3.13 mmol) was added to a solution of di-ester **13** (252 mg, 0.782 mmol) in THF (5 mL) at 0 °C. The mixture was stirred at r.t. for 4 h. Water (5 mL) was added slowly. The mixture was diluted with Et_2O (10 mL) and filtered. The filtrate was washed with water (10 mL). The organic layer was dried ($MgSO_4$), filtered, and concentrated. The residue was purified by flash column chromatography (1:2 hexane:EtOAc) to afford the title compound as a white crystalline solid (55.0 mg, 54%); **m.p.** 101.0 °C; **[α]D** +10.0 (*c* 0.30, MeOH); **IR** (neat) ν_{max} (cm^{-1}) 3275, 2946, 1560, 1438, 1059, 997, 815; **^{1}H NMR** (600 MHz, $CDCl_3$) δ 4.50 (m, 2H, H4 and H5), 3.62 (m, 4H, H1 and H8), 1.88–1.73 (m, 4H, H3 and H6), 1.73–1.62 (m, 4H, H2 and H7); **^{13}C{^{1}H} NMR** (150 MHz, $CDCl_3$) δ 93.2 (dd, *J* = 175.6, 20.2 Hz, C4 and C5), 61.1 (C1 and C8), 27.7 (t, *J* = 2.5 Hz, C2 and C7), 26.8 (dd, *J* = 16.3, 11.0 Hz, C3 and C6); **^{19}F NMR** (376 MHz, MeOD) δ –198.9 (m, 2F); **^{19}F{^{1}H} NMR** (376 MHz, MeOD) δ –198.9 (s, 2F); **HRMS** (ESI, +ve): $C_8H_{16}F_2O_2Na^+$ [MNa^+] requires *m/z* 205.1011, found 205.0993.

(4*R*,5*R*)-4,5-Difluorooctanedioic acid (15)

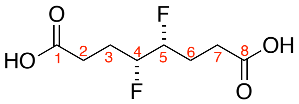

A mixture of di-ester **13** (50 mg, 0.16 mmol) and trifluoroacetic acid (1 mL) was stirred at r.t. for 2 h. The solvent was evaporated under a stream of N_2 to afford the title compound as a white solid (31 mg,

93%); **m.p.** 167.0–167.9 °C; **[α]$_D$** +22.2 (*c* 0.45, MeOH); **IR** (neat) ν_{max} (cm^{-1}) 2947, 1692, 1445, 1269, 1099, 898; **^{1}H NMR** (400 MHz, MeOD) δ 4.56 (m, 2H, H4 and H5), 2.49 (m, 4H, H2 and H7), 2.00 (m, 4H, H3 and H6); **^{13}C{^{1}H} NMR** (100 MHz, MeOD) δ 175.1 (C1 and C8), 92.3 (dd, *J* = 176.4, 20.4 Hz, C4 and C5), 28.7 (*t*, J = 2.3 Hz, C2 and C7), 25.7 (dd, *J* = 15.7, 11.6 Hz, C3 and C6); **^{19}F NMR** (376 MHz, MeOD) δ –201.4 (m, 2F); **^{19}F{^{1}H} NMR** (376 MHz, MeOD) δ –201.4 (s, 2F); **HRMS** (ESI, +ve): $C_8H_{12}F_2O_4Na^+$ [MNa^+] requires *m/z* 233.0596, found 233.0588.

(4*S*,5*S*)-4,5-Difluorooctanedioic acid (*ent*-15)

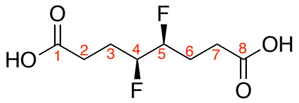

A mixture of *ent*-**13** (40 mg, 0.12 mmol) and trifluoroacetic acid (1 mL) was stirred at r.t. for 2 h. The solvent was evaporated under a stream of N_2 to afford the title compound as a white solid (20 mg, 80%); **m.p.** 160.7–161.0 °C; **[α]$_D$** –32.5 (*c* 0.80, MeOH); other spectroscopic data identical to those reported for **15**.

Di-*tert*-butyl (*Z*)-oct-4-enedioate (16)

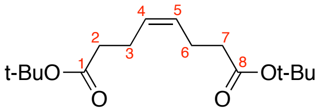

Pyridine (0.27 mL, 3.4 mmol) was added to a solution of (*Z*)-2-butene-1,4-diol (1.4 mL, 17 mmol) in Et_2O (40 mL) and MeCN (5 mL). The mixture was cooled to 0 °C, and PBr_3 (1.3 mL, 14 mmol) was added dropwise. The mixture was stirred at 0 °C for 30 min, and then heated at reflux for 4 h. Saturated aqueous $NaHCO_3$ solution (60 mL) was added, and the mixture was extracted with Et_2O (3 × 75 mL). The combined organic extracts were dried ($MgSO_4$), filtered, and concentrated. The residue was purified by flash chromatography (10:1 hexane:EtOAc) to afford (*Z*)-1,4-dibromo-2-butene as a clear oil (2.5 g, 69%); **^{1}H NMR** (400 MHz, $CDCl_3$) δ 5.92–5.84 (m, 2H, CH=CH), 4.02 (d, *J* = 2.1 Hz, 2H, 2×CH_2Br); **^{13}C{^{1}H} NMR** (100 MHz, $CDCl_3$) δ 129.8, 24.8. These spectroscopic data were in accordance with literature values.[2]

To a stirred solution of diisopropylamine (2.0 mL, 15 mmol) in THF (20 mL) at –78 °C was added *n*-butyllithium (1.6 M solution in hexanes, 9.1 mL, 15 mmol) dropwise. The resulting mixture was

stirred for 15 min before *t*-butyl acetate (2.0 mL, 15 mmol) was added dropwise. The solution was stirred at –78 °C for 30 minutes. Then, a solution of (*Z*)-1,4-dibromo-2-butene (1.0 g, 4.9 mmol) in THF (6 mL) was added dropwise at –78 °C. The mixture was stirred at –78 °C for 2 h. Saturated aqueous NH_4Cl solution (30 mL) was added, and the mixture was extracted with EtOAc (3 × 50 mL). The combined organic layers were dried ($MgSO_4$), filtered, and concentrated. The residue was purified by flash chromatography (20:1 hexane:EtOAc) to afford the title compound as a white solid (1.3 g, 94%); **m.p.** 61.0–62.0 °C; **IR** (neat) ν_{max} (cm^{-1}) 2977, 1728, 1367, 1149, 845; **^{1}H NMR** (400 MHz, $CDCl_3$) δ 5.33 (t, *J* = 4.7 Hz, 2H, H4 and H5), 2.32–2.25 (m, 4H, H2 and H7), 2.23–2.18 (m, 4H, H3 and H6), 1.40 (s, 18H, 9×CH_3); **^{13}C{^{1}H} NMR** (100 MHz, $CDCl_3$) δ 172.3, 128.9, 80.0, 35.4, 28.0, 22.9; **HRMS** (ESI, +ve): $C_{16}H_{28}O_4Na^+$ [MNa^+] requires *m/z* 307.1880, found 307.1873.

Di-*tert*-butyl (4*R*,5*S*)-4,5-difluorooctanedioate (17)

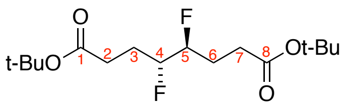

Trifluoromethanesulfonic anhydride (0.28 mL, 1.7 mmol) was added to a solution of fluorohydrin (±)-**27** (180 mg, 0.56 mmol) and pyridine (0.14 mL, 1.7 mmol) in CH_2Cl_2 (5 mL) at 0 °C. The mixture was stirred for 5 min 0 °C. The mixture was filtered and TBAF (1 M in THF, 5.7 mL, 5.7 mmol) was added to the filtrate at 0 °C. The mixture was stirred for 1 h at 0 °C. Brine (10 mL) was added and the mixture was extracted with CH_2Cl_2 (3 × 15 mL). The combined organic layers were dried ($MgSO_4$), filtered, and concentrated. The residue was purified by flash column chromatography (3:1 hexane:EtOAc) to afford the title compound as a clear oil (38 mg, 21%); **IR** (neat) ν_{max} (cm^{-1}) 2976, 1729, 1367, 1254, 1154, 848; **^{1}H NMR (**400 MHz, $CDCl_3$) δ 4.56 (m, 2H, H4 and H5), 2.44 (m, 4H, H2 and H7), 1.99 (m, 4H, H3 and H6), 1.47 (s, 18H, 6×CH_3); **^{13}C{^{1}H} NMR** (100 MHz, $CDCl_3$) δ 172.0 (C1 and C8), 92.5 (dd, *J* = 175.5, 25.3 Hz, C4 and C5), 80.7 ($\underline{C}(CH_3)_3$), 30.6 (t, *J* = 1.5 Hz, C2 and C7), 28.1 (6×CH_3), 25.6 (dd, *J* = 14.8, 11.4 Hz, C3 and C6); **^{19}F NMR** (282 MHz, $CDCl_3$) δ –194.8 (m, 2F); **^{19}F{^{1}H} NMR** (282 MHz, $CDCl_3$) δ –194.8 (s, 2F); **HRMS** (ESI, +ve): $C_{16}H_{28}F_2O_4Na^+$ [MNa^+] requires *m/z* 345.1848, found 345.1849.

(4*R*,5*S*)-4,5-Difluorooctane-1,8-diol (18)

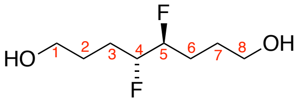

Lithium aluminium hydride (1 M in THF, 4.96 mL, 4.96 mmol) was added to a solution of di-ester **17** (400 mg, 1.24 mmol) in THF (5 mL) at 0 °C. The mixture was stirred at r.t. for 4 h. Water (5 mL) was added slowly. The mixture was diluted with Et_2O (10 mL) and filtered. The filtrate was washed with water (10 mL). The organic layer was dried ($MgSO_4$), filtered, and concentrated. The residue was purified by flash chromatography (1:1→1:2 hexane:EtOAc) to afford the title compound as a white crystalline solid (103 mg, 46%); **m.p.** 68.0–68.5 °C; **IR** (neat) ν_{max} (cm^{-1}) 3360, 2941, 1450, 1055; **^{1}H NMR** (600 MHz, MeOD) δ 4.54 (m, 2H, H4 and H5), 3.62 (m, 4H, H1 and H8), 1.81–1.62 (m, 8H, H2, H3, H6, and H7); **^{13}C{^{1}H} NMR** (150 MHz, MeOD) δ 94.5 (dd, J = 174.1, 24.6 Hz, C4 and C5), 61.1 (C1 and C8), 27.8 (C2 and C7), 26.2 (dd, J = 14.9, 11.0 Hz, C3 and C6); **^{19}F NMR** (376 MHz, MeOD) δ –192.7 (m, 2F); **^{19}F{^{1}H} NMR** (376 MHz, MeOD) δ (ppm) –192.7 (s, 2F, 2×CHF); **HRMS** (ESI, +ve): $C_8H_{16}F_2O_2Na^+$ [MNa^+] requires m/z 205.1011, found 205.1013.

Ethyl (*E*)-6-bromohex-2-enoate (19)[3]

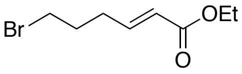

A solution of 4-bromobutanal (21.4 g, 140 mmol) and ethyl (triphenylphosphoranylidene)acetate (46.0 g, 140 mmol) in CH_2Cl_2 (100 mL) was stirred at r.t. overnight. The mixture was concentrated and the residue was triturated with hexane and filtered. The filtrate was concentrated to give the title compound as a yellow oil which was used in the following step without further purification (~47 g, quantitative); **^{1}H NMR** (300 MHz, $CDCl_3$) δ 6.93 (m, 1H, CH_2 $\underline{CH}$=CH), 5.88 (d, J = 15.9 Hz, 1H, $\underline{CH}OOCH_3$), 4.28 (t, J = 6.4 Hz, 2H, CH_2Br), 4.12 (q, J = 11.4, 7.1 Hz, 2H, OCH_2), 2.39 (m, 2H, $\underline{CH_2}$CH=CH), 2.02 (m, 2H, $\underline{CH_2}CH_2Br$), 1.25 (t, J = 6.8 Hz, CH_3); spectroscopic data in accordance with literature values.[3]

Ethyl (*E*)-6-(1,3-dioxo-1H-benzo[*de*]isoquinolin-2(3*H*)-yl)hex-2-enoate (20)

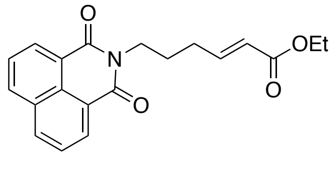

A mixture of naphthalimide (5.9 g, 30 mmol), K_2CO_3 (8.2 g, 59 mmol) and DMF (30 mL) was stirred at r.t. for 10 min. Alkyl bromide **19** (7.1 g, 30 mmol) was added, and the mixture was heated to 100 °C

overnight. The solution was cooled to r.t. and then filtered. The filtrate was extracted with CH_2Cl_2 (3 × 100 mL), dried over magnesium sulfate, filtered and concentrated onto silica. Flash column purification (4:1 hexane:EtOAc) afforded the title compound as a white solid (6.00 g, 59%); **m.p.** > 200 °C; **IR** (neat) ν_{max} (cm^{-1}) 1744, 1667, 1564; **^{1}H NMR** (400 MHz, $CDCl_3$) δ 8.59 (d, J = 7.2 Hz, 2H, ArH), 8.22 (d, J = 8.4 Hz, 2H, ArH), 7.79 (t, J = 7.7 Hz, 2H, ArH), 7.00 (m, 1H, CH=CHCO), 5.89 (d, J = 16.2 Hz, 1H, CH=CHCO), 4.23 (t, J = 7.5 Hz, 2H, CH_2N), 4.14 (q, J = 7.0 Hz, 2H, OCH_2), 2.36 (q, J = 7.5 Hz, 2H, CH_2CH=CH), 1.94 (m, 2H, CH_2CH_2N), 1.26 (t, J = 7.1 Hz, CH_3); **^{13}C{^{1}H} NMR** (75 MHz, $CDCl_3$) δ 166.3 (COOEt), 164.1, 164.0 (2 x NCO), 147.9 (CH=CHCO), 133.9, 131.6, 131.3 126.9, 122, 121.8 (CH=CHCO), 60.1 (OCH_2CH_3), 39.8 (NCH_2), 29.8, 26.4 ($NCH_2CH_2CH_2$), 14.2 (CH_3); **HRMS** (ESI, +ve) $C_{20}H_{19}FNO_4Na^+$ [M+Na^+] requires m/z 360.3810, found 360.3828.

Ethyl (2*R*,3*S*)-6-(1,3-dioxo-1*H*-benzo[*de*]isoquinolin-2(3*H*)-yl)-2,3-dihydroxyhexanoate (21)

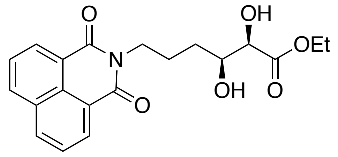

A mixture of alkene **20** (5.9 g, 17 mmol) and AD mix-α (25 g) in *t*-BuOH:H_2O (1:1, 85 mL) was stirred at r.t. for 10 min then cooled to 0 °C. Methanesulfonamide (1.6 g, 17 mmol) was added and the mixture was stirred at 0 °C for 48 h. Sodium sulfite (6 g) was added, and the mixture was stirred for 1 h. The mixture was extracted with EtOAc (2 × 25 mL) and CH_2Cl_2 (2 × 50 mL). The combined organic layers were dried over magnesium sulfate and concentrated onto silica. Flash column purification (4:1 hexane:EtOAc) afforded the title compound as a white solid (6.0 g, 94%, 95% *ee*); **m.p.** >200 °C; **$[\alpha]_D$** +17.8 (c = 1.00, CH_2Cl_2); **IR** (neat) ν_{max} (cm^{-1}) 3540, 3533, 1734, 1576; **^{1}H NMR** (400 MHz, $CDCl_3$) δ 8.56 (d, J = 7.3 Hz, 2H, ArH), 8.19 (d, J = 8.1 Hz, 2H, ArH), 7.73 (t, J = 7.7 Hz, 2H, ArH), 4.31–4.19 (m, 4H, OCH_2, 2 x CHOH), 4.12 (m, 1H, NCH), 4.00 (m, 1H, NCH), 2.05−1.55 (m, 4H, CH_2-CH_2), 1.29 (m, 3H, CH_2-CH_3); **^{13}C{^{1}H} NMR** (100 MHz, $CDCl_3$) δ 173.5 (COEt), 164.2 (2 x NCO), 134.0, 131.5, 128.1, 126.9, 122.5 (ArC), 73.4 (CHO), 72.4 (CHO), 62.0 (CH_2CO), 40.1 (NCH_2), 30.8, 24.5 ($NCH_2CH_2CH_2$), 14.1 (CH_3); **HRMS** (ESI, +ve) $C_{20}H_{21}NO_6Na^+$ [M+Na^+] requires m/z 394.1431, found 394.1442.

Ethyl (2*S*,3*S*)-6-(1,3-dioxo-1*H*-benzo[*de*]isoquinolin-2(3*H*)-yl)-2-fluoro-3-hydroxyhexanoate (22)

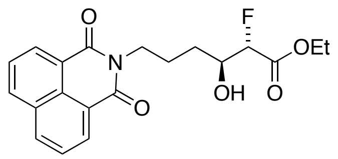

Thionyl chloride (2.5 mL, 32 mmol) was added to a solution of diol **21** (6.0 g, 16 mmol) and pyridine (3.8 mL, 48 mmol) in CH_2Cl_2 (187 mL) at 0 °C and the resulting mixture was stirred for 24 h. Saturated aqueous $CuSO_4$ (187 mL) was added, and the mixture was extracted with CH_2Cl_2 (3 × 200 mL). The combined organic layers were dried over magnesium sulfate, filtered and concentrated to give the cyclic sulfite intermediate as a white foam. This intermediate was dissolved in CH_2Cl_2:MeCN (1:1, 256 mL) and the solution cooled to 0 °C. $NaIO_4$ (6.8 g, 32 mmol), $RuCl_3.H_2O$ (~10 mg) and water (176 mL) were added, and the mixture was stirred at 0 °C for 24 h. Diethyl ether (500 mL) was added and the mixture was washed with water (500 mL), saturated $NaHCO_3$ (2 × 500 mL) and brine (2 × 500 mL). The organic layer was dried over magnesium sulfate, filtered and concentrated to give the cyclic sulfate intermediate as a white foam, which was used immediately in the next step. A mixture of TBAF (1 M solution in THF, 32 mL) and MeCN (266 mL) was dried over 3Å molecular sieves then added *via* cannula to an ice-cold flask containing the cyclic sulfate intermediate. The resulting mixture was stirred at r.t. overnight. The mixture was concentrated to give a brown oil. This residue was dissolved in a mixture of H_2SO_4 (1.3 mL), water (240 µL) and THF (240 mL), and the resulting solution was stirred at r.t. overnight. Brine (100 mL) and water (100 mL) were added and the mixture extracted with CH_2Cl_2 (2 × 100 mL). The combined organic layers were dried over magnesium sulfate, filtered and concentrated onto silica. Flash column purification (5:1 hexane:EtOAc) afforded the title compound as a clear oil (2 g, 34%); **[α]D** +30.2 (*c* = 1.00, $CHCl_3$); **IR** (neat) ν_{max} (cm^{-1}) 3433, 1722, 1545, 1401; **^{1}H NMR** (400 MHz, $CDCl_3$) δ 8.63 (d, *J* = 7.4 Hz, 2H, ArH), 8.24 (d, *J* = 8.2 Hz, 2H, ArH), 7.78 (t, *J* = 7.7 Hz, 2H, ArH), 4.90 (dd, *J* = 48.8, 4.0 Hz, 1H, CHF), 4.33–4.26 (m, 4H, $C\underline{H_2}$OE$\underline{t}$, $NC\underline{H_2}$), 4.17 (m, 1H, $C\underline{H}$OH), 1.97 (m, 2H, $C\underline{H_2}$CHF), 1.68 (m, 2H, $NCH_2C\underline{H_2}$), 1.33 (t, *J* = 7.10 Hz, 3H, CH_3); **^{19}F NMR** (282 MHz, $CDCl_3$) δ –202.1 (m, 1F); **HRMS** (ESI, +ve) $C_{20}H_{20}FNO_5H^+$ [$M+H^+$] requires *m/z* 374.1355, found 374.1354.

Ethyl (2*R*,3*R*)-6-(1,3-dioxo-1*H*-benzo[*de*]isoquinolin-2(3*H*)-yl)-2,3-difluorohexanoate (23) and ethyl (2*R*,3*S*)-6-(1,3-dioxo-1*H*-benzo[*de*]isoquinolin-2(3*H*)-yl)-2,3-difluorohexanoate (24)

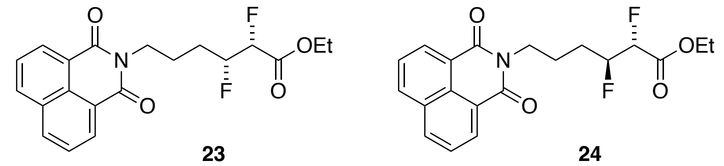

23 **24**

A mixture of fluorohydrin **22** (620 mg, 1.66 mmol) and Deoxofluor (50% in toluene, 6 mL, 17 mmol) was stirred at 70 °C in a plastic reaction vessel overnight, The mixture was cooled to 0 °C, diluted with CH_2Cl_2 and concentrated onto silica. Flash column purification (9:1→1:1, hexane:EtOAc) afforded two products **23** and **24** (2:1 ratio, combined yield 58%).

Data for **23**: clear oil (235 mg, 38%); **[α]D** +31.1 (*c* = 1.00, CH_2Cl_2); **IR** (neat) ν_{max} (cm^{-1}) 1744, 1532, 1403, 1398; **^{1}H NMR** (600 MHz, $CDCl_3$) δ 8.64 (d, *J* = 7.5 Hz, 2H, ArH), 8.25 (d, *J* = 7.5 Hz, ArH), 7.79 (t, *J* = 7.8 Hz, 2H, ArH), 5.09–4.87 (m, 2H, $C\underline{H}FC\underline{H}F$), 4.36–4.26 (m, 4H, $OC\underline{H}_2$, NCH_2), 1.92−2.01 (m, 4H, $NCH_2C\underline{H}_2C\underline{H}_2$), 1.35 (m, *J* = 7.1 Hz, 3H, $C\underline{H}_3$); **^{13}C{^{1}H} NMR** (75 MHz, $CDCl_3$) δ 168.5 ($\underline{C}OOEt$), 163.3 (2 x $N\underline{C}O$), 134.2, 131.6, 128.2, 127.0, 122.4, ($Ar\underline{C}$), 93.0–90.6 (m, 2 x CHF), 61.7 ($O\underline{C}H_2$), 42.8 ($N\underline{C}H_2$), 26.8, 26.6 ($NCH_2\underline{C}H_2\underline{C}H_2$), 14.1 ($CH_3$); **^{19}F NMR** (282 MHz, $CDCl_3$) δ –195.4 (m, 1F), –208.8 (m, 1F); **HRMS** (ESI, +ve) $C_{20}H_{19}F_2NO_4Na^+$ [M+Na^+] requires *m/z* 398.3721, found 398.3755.

Data for **24**: clear oil (124 mg, 20%); **[α]D** +30.3 (*c* = 1.00, CH_2Cl_2); **IR** (neat) ν_{max} (cm^{-1}) 1742, 1530, 1400, 1398; **^{1}H NMR** (600 MHz, $CDCl_3$) δ 8.63 (d, *J* = 7.3 Hz, 2H, ArH), 8.25 (d, *J* = 8.3 Hz, ArH), 7.78 (t, *J* = 7.7 Hz, 2H, ArH), 5.12 (ddd, *J* = 50.0, 17.2, 2.2 Hz, 1H, CHFCO), 4.97 (m, 1H, $C\underline{H_2}CHF$), 4.36–4.20 (m, 4H, OCH_2, $NC\underline{H_2}$), 2.02–1.83 (m, 4H, $NCH_2C\underline{H}_2C\underline{H}_2$), 1.34 (d, *J* = 7.1 Hz, 3H, CH_3); **^{13}C{^{1}H} NMR** (75 MHz, $CDCl_3$) δ 164.2 ($\underline{C}OOEt$), 160.4 (2 x NC=O), 141.1, 134.0, 131.3, 126.9, 122.6 (ArC), 119.6 (dd, 182.2, 18.4 Hz, $\underline{C}HFCO$, in baseline, overlapping 119.4), 119.4 (dd, *J* = 183.1, 17.3 Hz, CHF, in baseline, overlapping 119.6), 61.5 (OCH_2), 39.7 (NCH_2), 26.2 (d, *J* = 22.1 Hz, $NCH_2\underline{C}H_2$) 22.1 (d, *J* = 21.8 Hz ($NCH_2CH_2\underline{C}H_2$), 14.1 ($CH_3$); **^{19}F NMR** (282 MHz, $CDCl_3$) δ –191.1 (m, 1F), –202.2 (m, 1F); **HRMS** (ESI, +ve) $C_20H_{19}F_2NO_4Na^+$ [M+Na^+] requires *m/z* 398.3721, found 398.3735.

(4*R*,5*R*)-4,5-Difluoro-8-oxo-8-(phenylamino)octanoic acid (25)

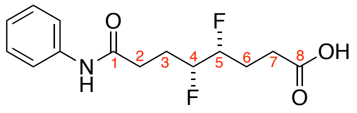

N,*N*′-Diisopropylcarbodiimide (0.017 mL, 0.11 mmol) was added to a solution of di-acid **15** (23 mg, 0.11 mmol), HOBt (15 mg, 0.11 mmol) and aniline (0.015 mL, 0.16 mmol) in DMF (3 mL) at r.t. The mixture was stirred overnight at r.t.. Aqueous hydrochloric acid (1 M) was added, and the mixture was extracted with CH_2Cl_2 (3×). The combined organic layers were dried ($MgSO_4$), filtered, and concentrated. The residue was subjected to preparative reverse-phase HPLC employing 0.1% formic acid/H_2O as eluent A and 0.1% formic acid/MeCN as eluent B (gradient 98% A for 2 min, then ramped to 100% B over 40 min, then maintained at 100% B for 6 min) to afford the title compound as a white solid (7.0 mg, 23%); **m.p.** 158.0 °C; **[α]D** +19.4 (*c* 0.31, MeOH); **IR** (neat) ν_{max} (cm^{-1}) 2971, 2900, 1649, 1424, 1066, 900; **^{1}H NMR** (400 MHz, MeOD) δ 7.56 (d, 2H, *J* = 8.5 Hz, ArH), 7.31 (t, 2H, *J* = 8.3 Hz, ArH), 7.10 (t, 1H, *J* = 7.5 Hz, ArH), 4.71–4.47 (m, 2H, H4 and H5), 2.58 (m, 2H, H7), 2.49 (m, 2H, H2), 2.14 (m, 2H, H6), 1.98 (m, 2H, H3); **^{13}C{^{1}H} NMR** (100 MHz, MeOD) δ 175.3 (C1), 171.9 (C8), 138.4 (ArC), 128.4 (ArC), 123.8 (ArC), 120.0 (ArC), 92.5 (dd, *J* = 175.7, 19.8 Hz, C4), 92.3 (dd, *J* = 178.9, 19.2 Hz, C5), 31.6 (d, *J* = 4.3 Hz, C7), 29.0 (d, *J* = 4.6 Hz, C2), 26.2 (dd, *J* = 21.4, 5.5 Hz, C6), 25.8 (dd, *J* = 21.6, 5.7 Hz, C3); **^{19}F NMR** (376 MHz, MeOD) δ –200.8 (m, 1F), –201.2 (m, 1F); **^{19}F{^{1}H} NMR** (376 MHz, MeOD) δ –200.8 (d, 1F, *J* = 9.8 Hz), –201.2 (d, 1F, *J* = 9.8 Hz); **HRMS** (ESI, +ve): $C_{14}H_{18}F_2NO_3$ [MH^+] requires *m/z* 286.1249, found 286.1247.

(4*S*,5*S*)-4,5-Difluoro-8-oxo-8-(phenylamino)octanoic acid (*ent*-25)

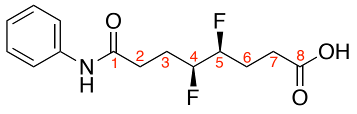

N,*N*′-Diisopropylcarbodiimide (0.025 mL, 0.16 mmol) was added to a solution of *ent*-**15** (28 mg, 0.13 mmol), HOBt (22 mg, 0.16 mmol) and aniline (0.018 mL, 0.20 mmol) in DMF (3 mL) at r.t., and the mixture was stirred overnight at r.t.. Aqueous hydrochloric acid (1 M) was added, and the mixture was extracted with CH_2Cl_2 (3×). The combined organic layers were dried ($MgSO_4$), filtered, and concentrated. The residue was subjected to preparative reverse-phase HPLC employing 0.1% formic acid/H_2O as eluent A and 0.1% formic acid/MeCN as eluent B (gradient 98% A for 2 min, then ramped to 100% B over 40 min, then maintained at 100% B for 6 min) to afford the title

compound as a white solid (9.0 mg, 24%); **m.p.** 155.6–156.1 °C; **[α]D** –17.8 (*c* 0.45, MeOH); **IR** (neat) ν_{max} (cm^{-1}) 3327, 1659, 1602, 1542, 1443; **^{1}H NMR** (400 MHz, MeOD) δ 7.56 (d, 2H, *J* = 8.5 Hz, ArH), 7.32 (t, 2H, *J* = 8.0 Hz, ArH), 7.10 (t, 1H, *J* = 7.5 Hz, ArH), 4.70–4.48 (m, 2H, H4 and H5), 2.58 (m, 2H, H7), 2.49 (m, 2H, H2), 2.15 (m, 2H, H6), 1.98 (m, 2H, H3); **^{13}C{^{1}H} NMR** (100 MHz, MeOD) δ 175.2 (C1), 171.9 (C8), 138.4 (ArC), 128.4 (ArC), 123.8 (ArC), 119.9 (ArC), 92.5 (dd, *J* = 176.0, 19.2 Hz, C4), 92.3 (dd, *J* = 176.0, 19.2 Hz, C5), 31.6 (d, *J* = 4.0 Hz, C7), 28.9 (d, *J* = 4.2 Hz, C2), 26.2 (dd, *J* = 21.9, 5.8 Hz, C6), 25.8 (dd, *J* = 21.9, 5.8 Hz, C3); **^{19}F NMR** (376 MHz, MeOD) δ –200.8 (m, 1F), –201.2 (m, 1F); **^{19}F{^{1}H} NMR** (376 MHz, MeOD) δ –200.8 (d, 1F, *J* = 9.8 Hz), –201.2 (d, 1F, *J* = 9.8 Hz); **HRMS** (ESI, +ve): $C_{14}H_{18}F_2NO_3$ [MNa^{+}] requires *m/z* 308.1069, found 308.1079.

Di-tert-butyl (4*R*,5*S*)-4,5-dihydroxyoctanedioate (26)

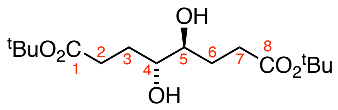

A mixture of AD-mix-α (1.70 g) and methanesulfonamide (117 mg, 1.23 mmol) was stirred in *t*-BuOH (10 mL) and water (10 mL) at r.t. until a homogenous solution formed. Then, a solution of alkene **16** (250 mg, 1.23 mmol) in *t*-BuOH (5 mL), water (5 mL), and acetone (5 mL) was added. The mixture was stirred at r.t. overnight. Saturated aqueous Na_2SO_3 solution was added, and the mixture was extracted with CH_2Cl_2 (3 × 25 mL). The combined organic layers were dried ($MgSO_4$), filtered, and concentrated. The residue was purified by flash chromatography (2:1 hexane:EtOAc) to afford the diol shown in the scheme above as a white solid (240 mg, 87%); **m.p.** 81–82 °C; **IR** (neat) ν_{max} (cm^{-1}) 3499, 2977, 2878, 1699, 1459, 1342, 1150, 1061, 921, 839; **^{1}H NMR** (400 MHz, $CDCl_3$) δ 3.57 (m, 2H, H4 and H5), 2.99 (s, 2H, 2×OH), 2.51–2.38 (m, 4H, H2 and H7), 1.86–1.71 (m, 4H, H3 and H6), 1.46 (s, 18H, 6×CH_3); **^{13}C{^{1}H} NMR** (100 MHz, $CDCl_3$) δ 174.1, 80.7, 74.1, 32.2, 28.1, 26.7; **HRMS** (ESI, +ve): $C_{16}H_{30}O_6Na^{+}$ [MNa^{+}] requires *m/z* 341.1935, found 341.1929.

Di-tert-butyl (4*R*,5*R*)-4-fluoro-5-hydroxyoctanedioate ((±)-27)

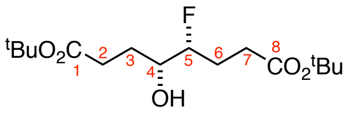

Thionyl chloride (0.280 mL, 3.84 mmol) was added to a solution of diol **26** (612 mg, 1.92 mmol) and pyridine (0.470 mL, 5.77 mmol) in dry CH_2Cl_2 (20 mL) at 0 °C. The mixture was stirred for 30 min at 0 °C. Saturated aqueous $CuSO_4$ solution (20 mL) was added and the mixture was extracted with CH_2Cl_2 (3 × 25 mL). The organic layers were combined, dried ($MgSO_4$), and concentrated to give an intermediate cyclic sulfite as yellow oil. This residue was dissolved in acetonitrile (12 mL) and CH_2Cl_2 (12 mL) and cooled to 0 °C. Sodium metaperiodate (821 mg, 3.84 mmol), ruthenium chloride hydrate (150 mg) and water (2 mL) were added, and the mixture was stirred at 0 °C for 3 h. Diethyl ether (30 mL) was added, and the mixture was washed with water (30 mL), saturated aqueous $NaHCO_3$ (30 mL) and brine (30 mL). The organic layer was dried ($MgSO_4$), filtered, and concentrated to give an intermediate cyclic sulfate as a clear oil. This ressidue was dissolved in MeCN (13 mL), and the solution was cooled to 0 °C. A solution of TBAF (1 M in THF, 3.84 mL, 3.84 mmol) was added, and the mixture was stirred overnight at r.t. Brine (20 mL) was added and the mixture was extracted with CH_2Cl_2 (3 × 30 mL). The combined organic layers were dried ($MgSO_4$), filtered, and concentrated to give yellow oil. This residue was dissolved in dioxane (57 mL) and *p*-toluenesulfonic acid (547 mg, 2.88 mmol) was added. The mixture was stirred for 1.5 h at r.t. Saturated $NaHCO_3$ solution (100 mL) was added, and the mixture was extracted with CH_2Cl_2 (3 × 70 mL). The combined organic layers were dried ($MgSO_4$), filtered, and concentrated. The residue was purified by flash chromatography (4:1 hexane:EtOAc) to afford the fluorohydrin shown in the scheme above as a clear oil (430 mg, 70%); **IR** (neat) ν_{max} (cm^{-1}) 3402, 2976, 2929, 1726, 1367, 1255, 1152, 847; **^{1}H NMR** (600 MHz, $CDCl_3$) δ 4.40 (ddt, *J* = 48.8, 9.3, 4.1 Hz, 1H, H5), 3.64 (m, 1H, H4), 2.45 (m, 2H, H7), 2.39 (m, 2H, H2), 1.97 (m, 2H, H3), 1.83 (m, 2H, H6), 1.47 (s, 18H, 6×CH_3); **^{13}C{^{1}H} NMR** (150 MHz, $CDCl_3$) δ 173.2 (C1), 172.3 (C8), 95.4 (d, *J* = 171.5 Hz, C4), 80.6 ($\underline{C}(CH_3)_3$), 80.6 ($\underline{C}(CH_3)_3$), 72.1 (d, *J* = 20.0 Hz, C5), 31.7 (C7), 30.9 (d, *J* = 4.5 Hz, C2), 28.1 (6×CH_3), 27.8 (d, *J* = 4.9 Hz, C6), 26.4 (d, *J* = 21.5 Hz, C3); **^{19}F NMR** (376 MHz, $CDCl_3$) δ –197.0 (m, 1F); **^{19}F{^{1}H} NMR** (376 MHz, $CDCl_3$) δ –197.0 (s, 1F); **HRMS** (ESI, +ve): $C_{16}H_{29}FO_5Na^+$ [MNa^+] requires *m/z* 343.1891, found 343.1893.

(4*R*,5*S*)-4,5-Difluorooctanedioic acid (28)

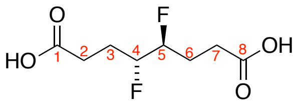

A mixture of di-ester **17** (29 mg, 0.090 mmol) and trifluoroacetic acid (1 mL) was stirred at r.t. for 2 h. The solvent was evaporated under a stream of N_2 to afford the title compound as a white solid

(21 mg, 100%); **m.p.** 137.0–137.7 °C; **IR** (neat) ν_{max} (cm^{-1}) 2938, 1695, 1432, 1267, 1211, 1049; **^{1}H NMR** (400 MHz, MeOD) δ 4.62 (m, 2H, H4 and H5), 2.50 (m, 4H, H2 and H7), 1.97 (m, 4H, H3 and H6); **^{13}C{^{1}H} NMR** (100 MHz, MeOD) δ 175.1 (C1 and C8), 92.6 (dd, *J* = 174.5, 24.7 Hz, C4 and C5), 28.8 (*t*, J = 2.1 Hz, C2 and C7), 25.1 (dd, *J* = 15.3, 12.5 Hz, C3 and C6); **^{19}F NMR** (376 MHz, MeOD) δ –196.4 (m, 2F); **^{19}F{^{1}H} NMR** (376 MHz, MeOD) δ –196.4 (s, 2F); **HRMS** (ESI, +ve): $C_8H_{12}F_2O_4Na^+$ [MNa$^+$] requires *m/z* 233.0596, found 233.0590.

(±)-(4*S*,5*R*)-4,5-Difluoro-8-oxo-8-(phenylamino)octanoic acid ((±)-29)

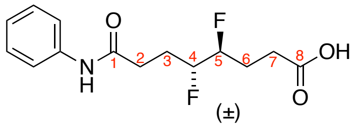

N,*N'*-Diisopropylcarbodiimide (0.017 mL, 0.11 mmol) was added to a solution of di-acid **28** (21 mg, 0.099 mmol), HOBt (15 mg, 0.11 mmol) and aniline (0.014 mL, 0.15 mmol) in DMF (1.5 mL) at r.t., and the mixture was stirred overnight at r.t.. Aqueous hydrochloric acid (1 M) was added, and the mixture was extracted with CH_2Cl_2 (3×). The combined organic layers were dried ($MgSO_4$), filtered, and concentrated. The residue was subjected to preparative reverse-phase HPLC employing 0.1% formic acid/H_2O as eluent A and 0.1% formic acid/MeCN as eluent B (gradient 98% A for 2 min, then ramped to 100% B over 40 min, then maintained at 100% B for 6 min) to afford the title compound as a white solid (10 mg, 35%); **m.p.** 123.4–123.6 °C; **IR** (neat) ν_{max} (cm^{-1}) 3304, 1695, 1654, 1499, 1418, 1046; **^{1}H NMR** (400 MHz, MeOD) δ 7.56 (d, 2H, *J* = 8.9 Hz, ArH), 7.32 (t, 2H, *J* = 7.5 Hz, ArH), 7.10 (t, 1H, *J* = 7.3 Hz, ArH), 4.75–4.55 (m, 2H, H4 and H5), 2.61 (m, 2H, H7), 2.49 (m, 2H, H2), 2.20–1.92 (m, 4H, H3 and H6); **^{13}C{^{1}H} NMR** (100 MHz, MeOD) δ 175.3 (C1), 171.8 (C8), 138.4 (ArC), 128.4 (ArC), 123.8 (ArC), 119.9 (ArC), 92.8 (dd, *J* = 171.9, 17.9 Hz, C4), 92.6 (dd, *J* = 171.1, 19.1 Hz, C5), 31.6 (d, *J* = 3.4 Hz, C7), 28.9 (d, *J* = 3.6 Hz, C2), 25.6 (dd, *J* = 20.9, 5.7 Hz, C6), 25.2 (dd, *J* = 20.9, 5.5 Hz, C3); **^{19}F NMR** (376 MHz, MeOD) δ –195.8 (m, 1F), –196.1 (m, 1F); **^{19}F{^{1}H} NMR** (376 MHz, MeOD) δ –195.8 (d, 1F, *J* = 13.5 Hz), –196.1 (d, 1F, *J* = 13.5 Hz); **HRMS** (ESI, +ve): $C_8H_{12}F_2O_4Na^+$ [MNa$^+$] requires *m/z* 308.1069, found 308.1063.

(*E*)-Oct-4-enedioic acid (30)

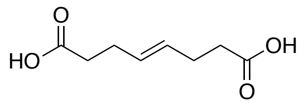

A mixture of di-ester **10** (227 mg, 0.798 mmol) and trifluoroacetic acid (2 mL) was stirred at r.t. for 2 h. The solvent was evaporated under a stream of N_2 to afford the crude title compound (137 mg, 99%), which was used for the next reaction without further purification.

(*E*)-8-Oxo-8-(phenylamino)oct-4-enoic acid (31)

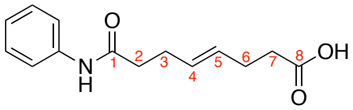

N,*N'*-Diisopropylcarbodiimide (0.052 mL, 0.30 mmol) was added to a solution of di-acid **30** (53 mg, 0.30 mmol), HOBt (45 mg, 0.30 mmol) and aniline (0.042 mL, 0.46 mmol) in DMF (5 mL) at r.t., and the mixture was stirred overnight at r.t.. Aqueous hydrochloric acid (1 M) was added, and the mixture was extracted with CH_2Cl_2 (3×). The combined organic layers were dried ($MgSO_4$), filtered, and concentrated. The residue was subjected to preparative reverse-phase HPLC employing 0.1% formic acid/H_2O as eluent A and 0.1% formic acid/MeCN as eluent B (gradient 98% A for 2 min, then ramped to 100% B over 40 min, then maintained at 100% B for 6 min) to afford the title compound as a white solid (6.0 mg, 10%); **m.p.** 102.4–102.8 °C; **IR** (neat) ν_{max} (cm^{-1}) 3303, 1709, 1598, 1443, 1310, 1177, 756; **^{1}H NMR** (400 MHz, MeOD) δ 7.54 (d, 2H, *J* = 8.0 Hz, ArH), 7.31 (t, 2H, *J* = 7.6 Hz, ArH), 7.09 (t, 1H, *J* = 7.3 Hz, ArH), 5.58 (m, 2H, H4 and H5), 2.46–2.36 (m, 4H, H3 and H6), 2.31–2.26 (m, 4H, H2 and H7); **^{13}C{^{1}H} NMR** (100 MHz, MeOD) δ 172.5, 138.4, 129.6, 129.2, 128.4, 123.7, 119.9, 36.5, 33.7, 28.4, 27.6; **HRMS** (ESI, +ve): $C_{14}H_{17}NO_3Na^+$ [MNa^+] requires *m/z* 270.1101, found 270.1092.

(*Z*)-Oct-4-enedioic acid (32)

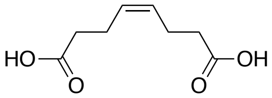

A mixture of di-ester **16** (131 mg, 0.461 mmol) and trifluoroacetic acid (2 mL) was stirred at r.t. for 2 h. The solvent was evaporated under a stream of N_2 to afford the crude title compound (86 mg), which was used for the next reaction without further purification.

(*Z*)-8-Oxo-8-(phenylamino)oct-4-enoic acid (33)

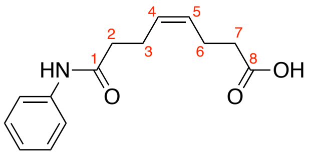

N,*N'*-Diisopropylcarbodiimide (0.026 mL, 0.17 mmol) was added to a solution of di-acid **32** (27 mg, 0.16 mmol), HOBt (23 mg, 0.17 mmol) and aniline (0.021 mL, 0.23 mmol) in DMF (3 mL) at r.t., and the mixture was stirred overnight at r.t.. Aqueous hydrochloric acid (1 M) was added, and the mixture was extracted with CH_2Cl_2 (3×). The combined organic layers were dried ($MgSO_4$), filtered, and concentrated. The residue was subjected to preparative reverse-phase HPLC employing 0.1% formic acid/H_2O as eluent A and 0.1% formic acid/MeCN as eluent B (gradient 98% A for 2 min, then ramped to 100% B over 40 min, then maintained at 100% B for 6 min) to afford the title compound as a white solid (19 mg, 48%); **m.p.** 67.6–68.3 °C; **IR** (neat) ν_{max} (cm^{-1}) 3299, 1710, 1659, 1598, 1543, 1442; **^{1}H NMR** (400 MHz, MeOD) δ 7.55 (d, 2H, *J* = 7.8 Hz, ArH), 7.31 (t, 2H, *J* = 7.8 Hz, ArH), 7.09 (t, 1H, *J* = 7.5 Hz, ArH), 5.47 (m, 2H, H4 and H5), 2.52–2.41 (m, 4H, H3 and H6), 2.41–2.32 (m, 4H, H2 and H7); **^{13}C{^{1}H} NMR** (150 MHz, MeOD) δ 175.6, 172.5, 138.4, 129.0, 128.7, 128.4, 123.8, 119.9, 36.5, 33.6, 23.2, 22.4; **HRMS** (ESI, +ve): $C_{14}H_{17}NO_3Na^+$ [MNa^+] requires *m/z* 270.1101, found 270.1102.

3. Measurement of optical purity of compounds 12 and 21

Measurement of optical purity of 12 through Mosher esterification

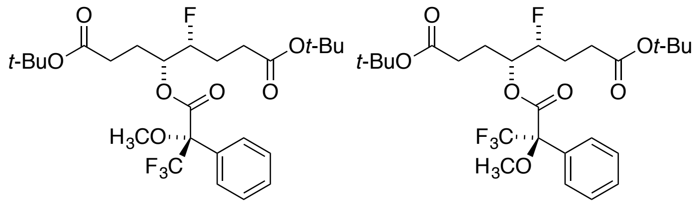

To a solution of fluorohydrin **12** (17 mg, 0.053 mmol) and (*S*)-α-methoxy-α-(trifluoromethyl)phenylacetic acid (37 mg, 0.16 mmol) in CH_2Cl_2 (1 mL) was added DCC (33 mg, 0.16 mmol) and DMAP (20 mg, 0.16 mmol). The resulting mixture was stirred at r.t. overnight. The mixture was filtered through a cotton wool plug and concentrated. Analysis of the residue by ^{19}F NMR ($CDCl_3$) revealed signals at –193.2 and –193.3 ppm that had an integral ratio of 3:97 (see spectrum below), suggesting that fluorohydrin **12** had 94% *ee*.

Repetition of this procedure using the opposite enantiomer of Mosher's acid gave ^{19}F NMR signals at –193.2 and –193.3 ppm with an integral ratio of 96:4 (see spectrum below), suggesting that fluorohydrin **12** had 91% *ee*.

A similar analysis was performed for *ent*-**12** (see spectra below).

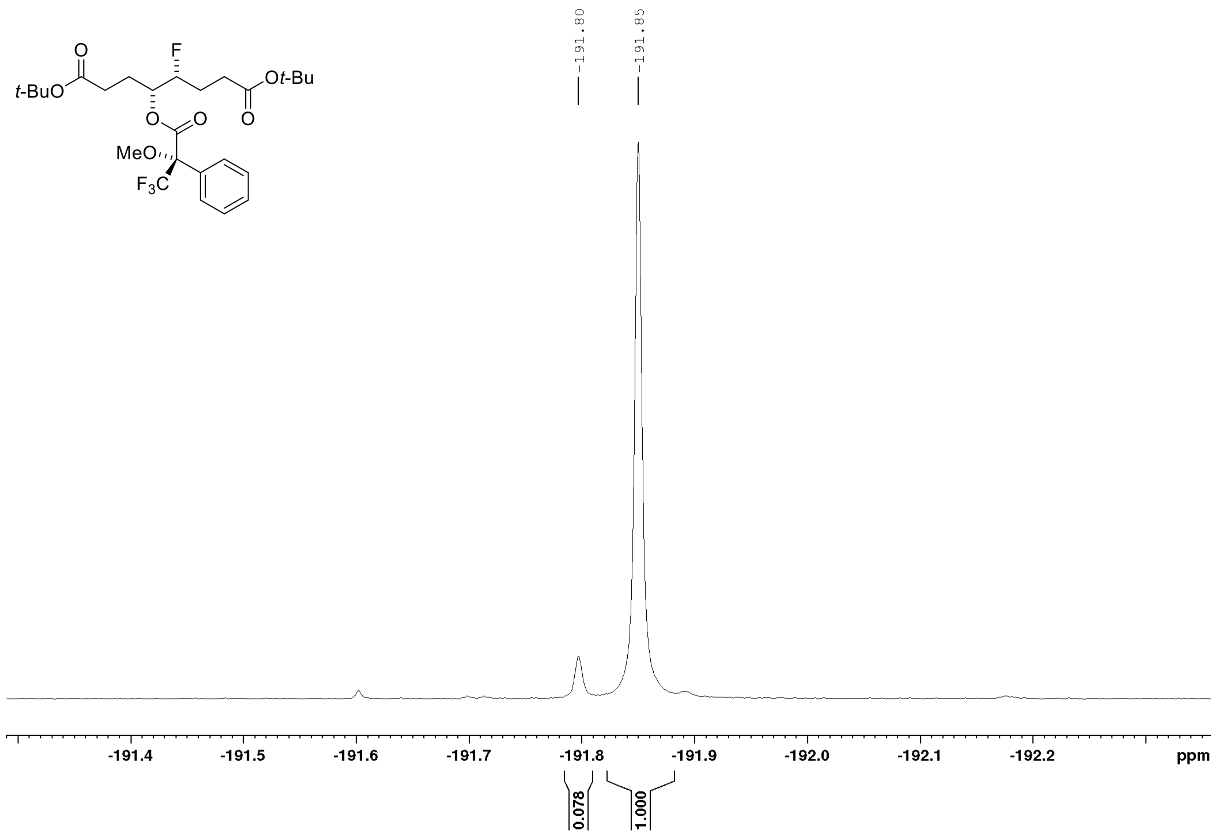
t-BuO
F
Ot-Bu
MeO
F3C
O
-191.80
-191.85
-191.4
-191.5
-191.6
-191.7
-191.8
-191.9
-192.0
-192.1
-192.2
ppm
0.078
1.000

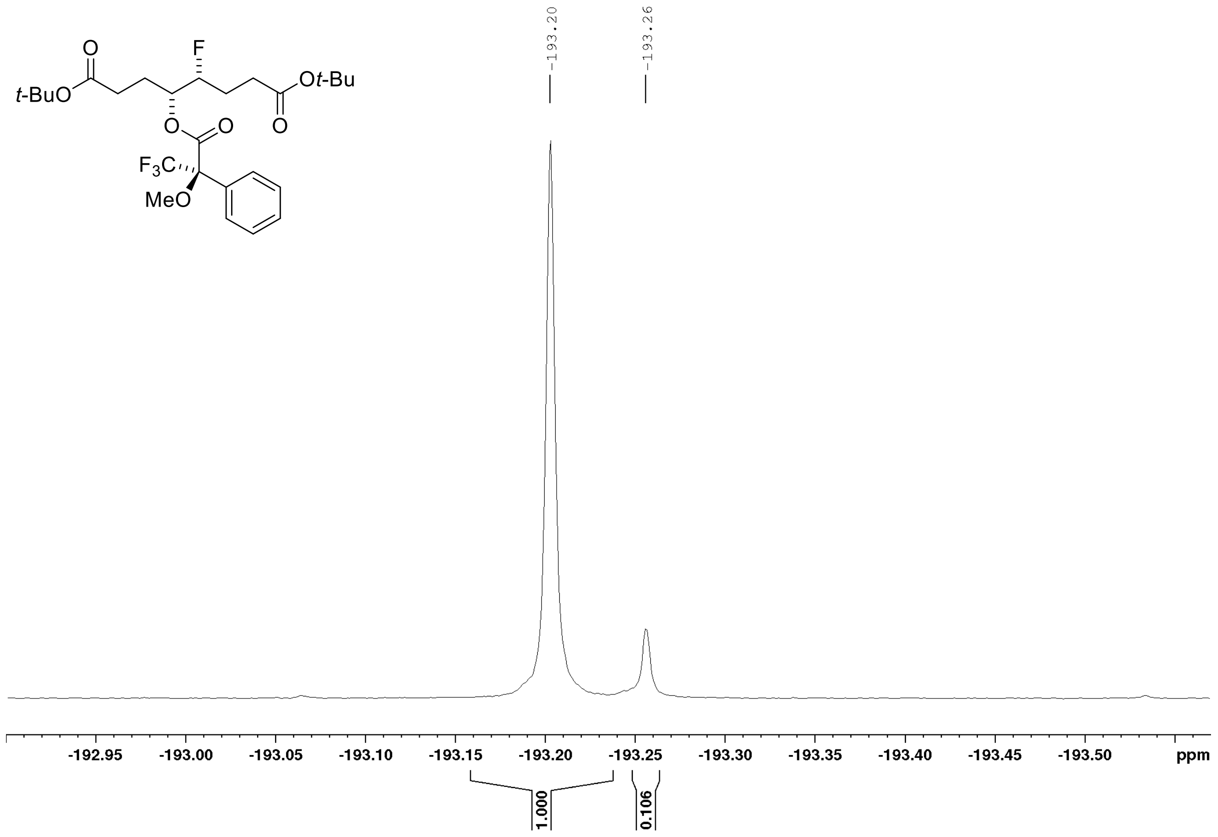
t-BuO
F
Ot-Bu
F3C
MeO
O
-193.20
-193.26
-192.95
-193.00
-193.05
-193.10
-193.15
-193.20
-193.25
-193.30
-193.35
-193.40
-193.45
-193.50
ppm
1.000
0.106

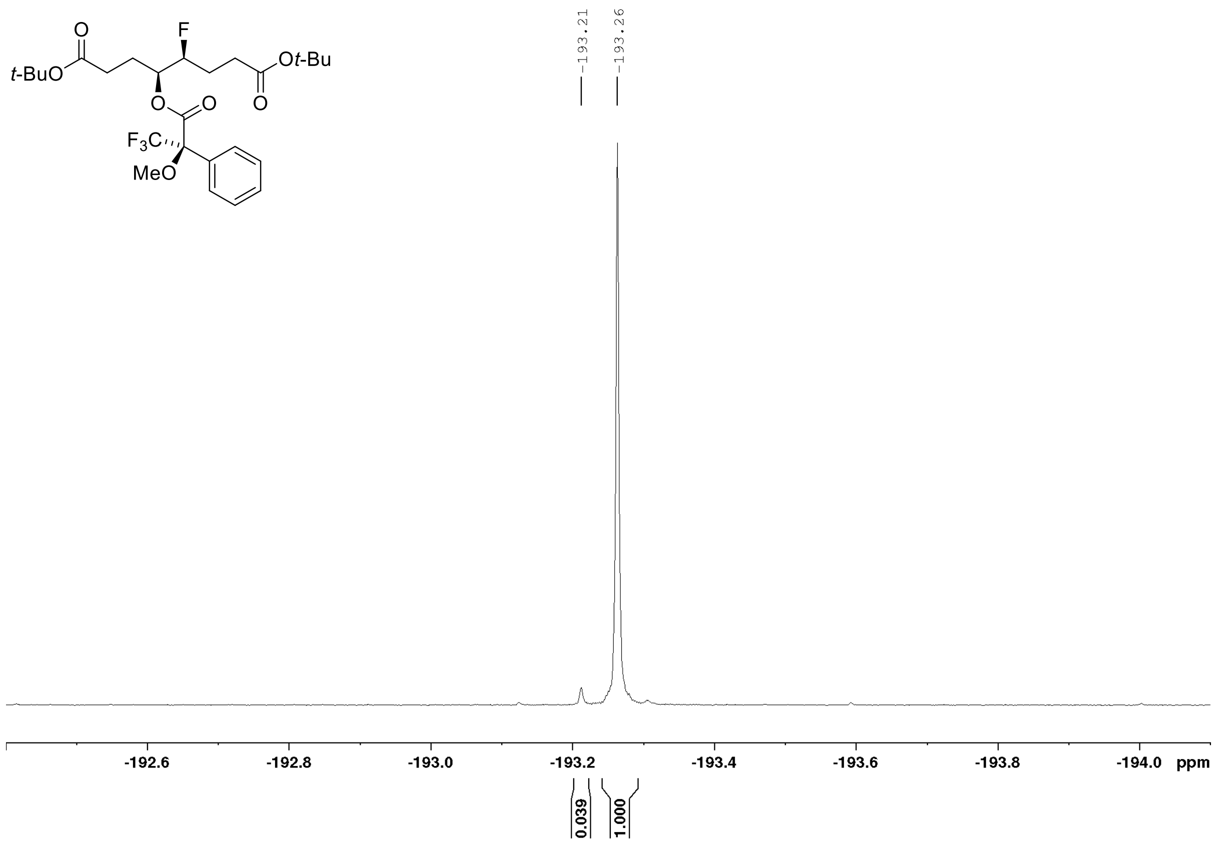
t-BuO
F
Ot-Bu
F3C
MeO
-193.21
-193.26
-192.6
-192.8
-193.0
-193.2
-193.4
-193.6
-193.8
-194.0
ppm
0.039
1.000

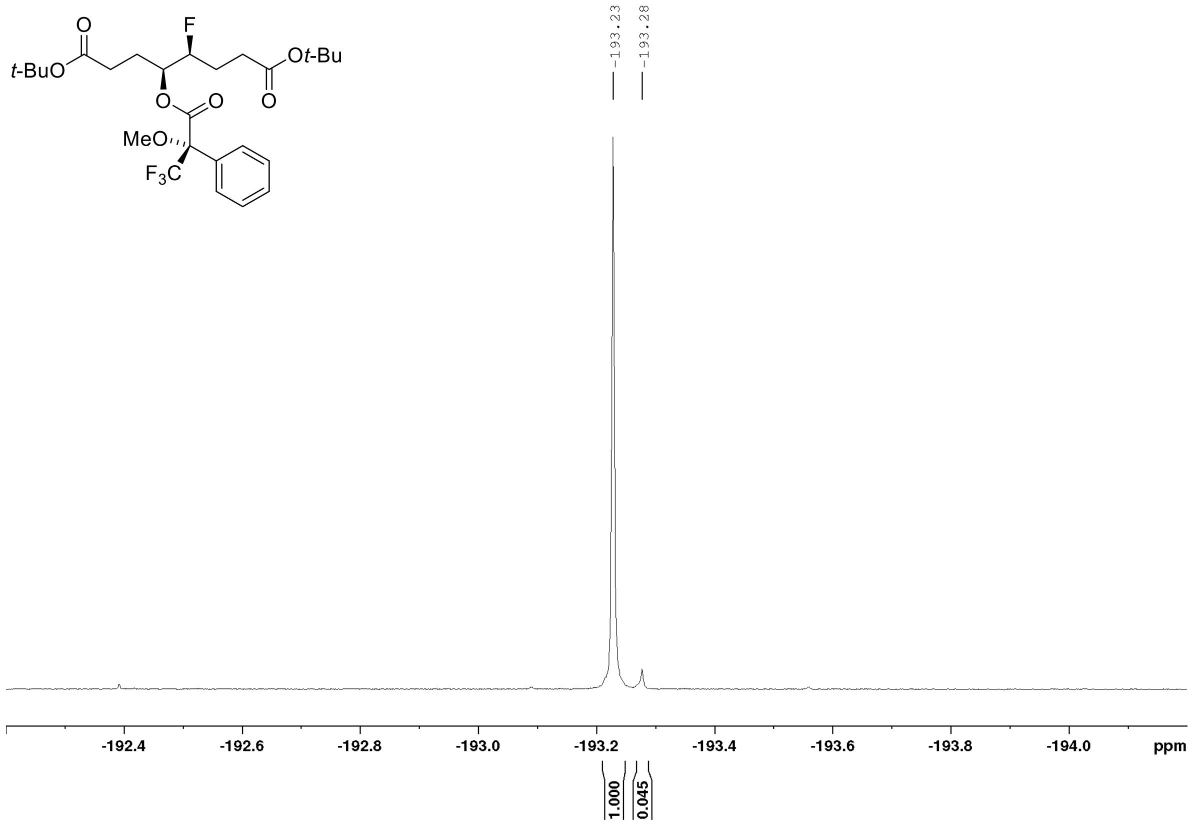
t-BuO
F
Ot-Bu
MeO
F3C
-193.23
-193.28
-192.4
-192.6
-192.8
-193.0
-193.2
-193.4
-193.6
-193.8
-194.0
ppm
1.000
0.045

Measurement of optical purity of 21 through chiral LC-MS

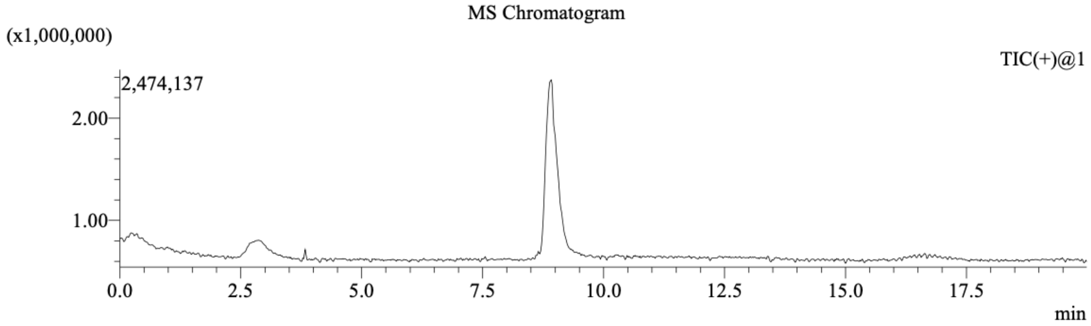

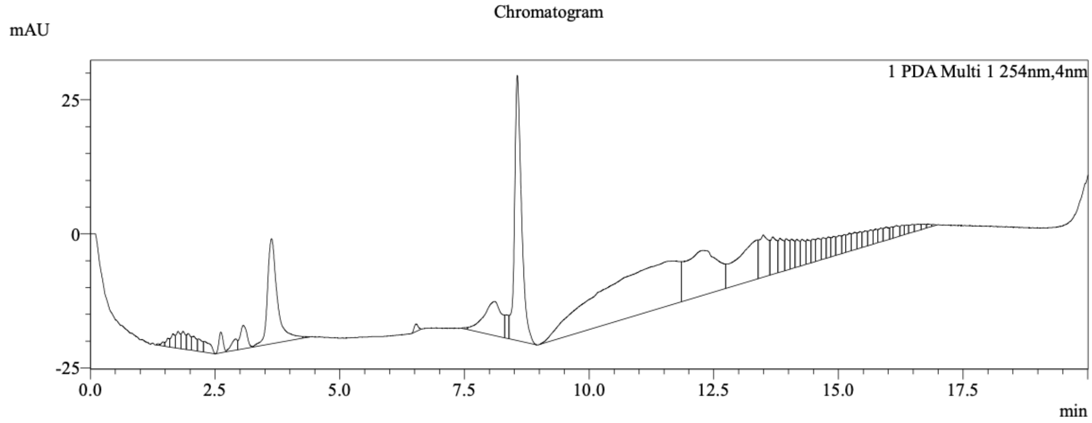

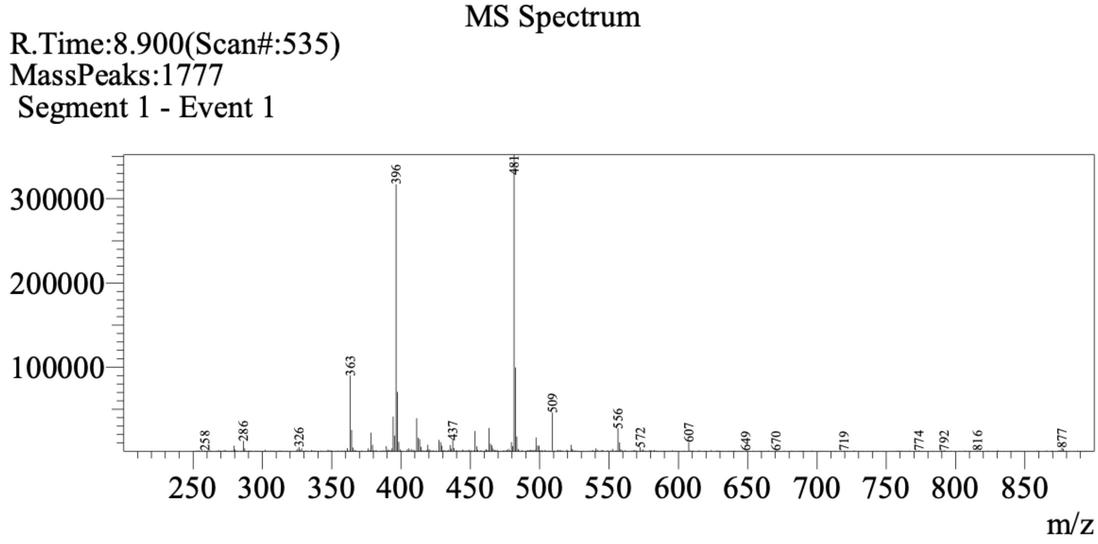

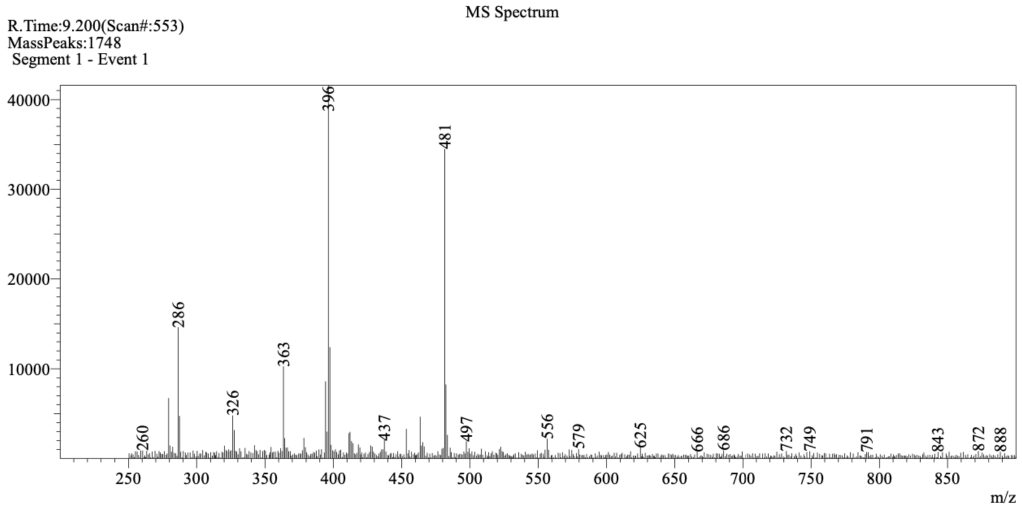

4. NMR spectra of novel compounds

^{1}H NMR (600 MHz, MeOD) of **2**

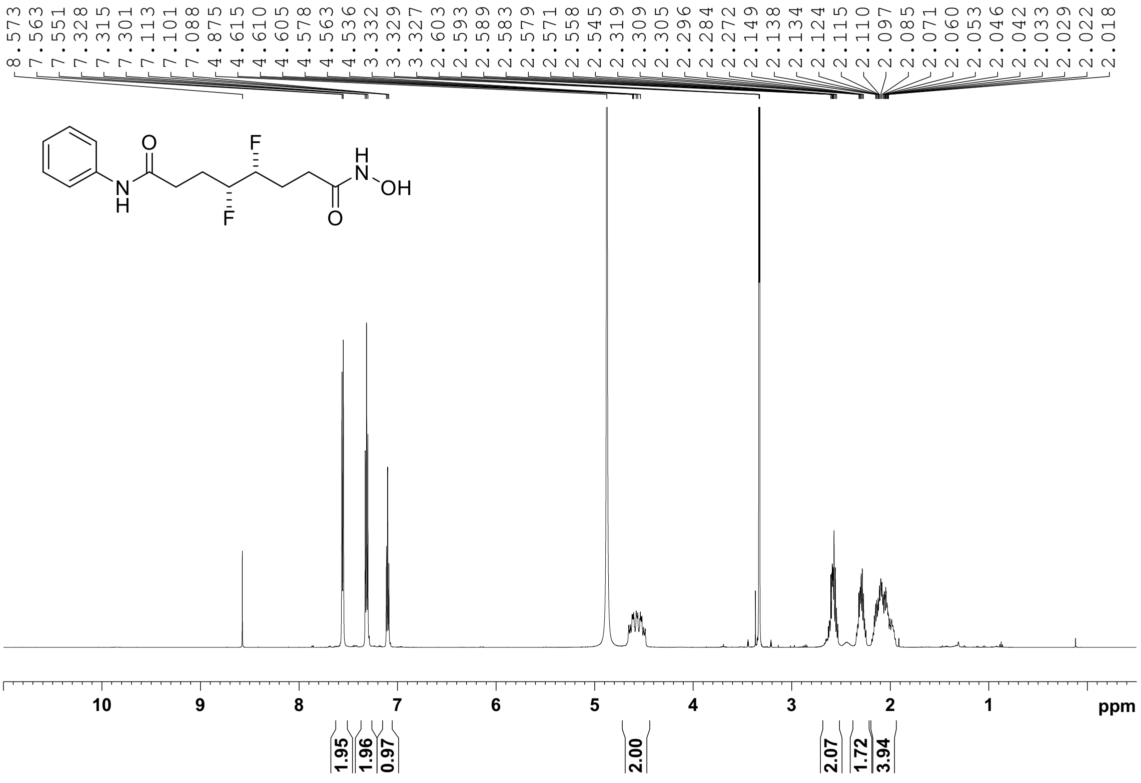

$^{13}C\{^{1}H\}$ NMR (150 MHz, MeOD) of **2**

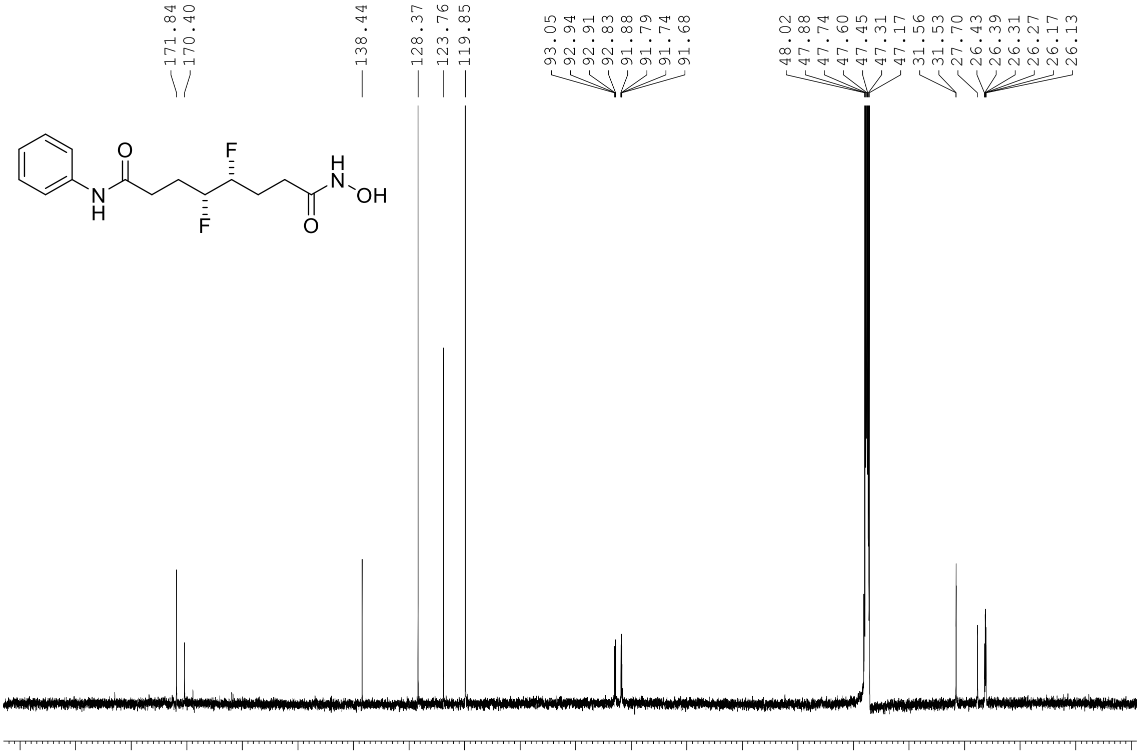

^{19}F NMR (376 MHz, MeOD) of **2**

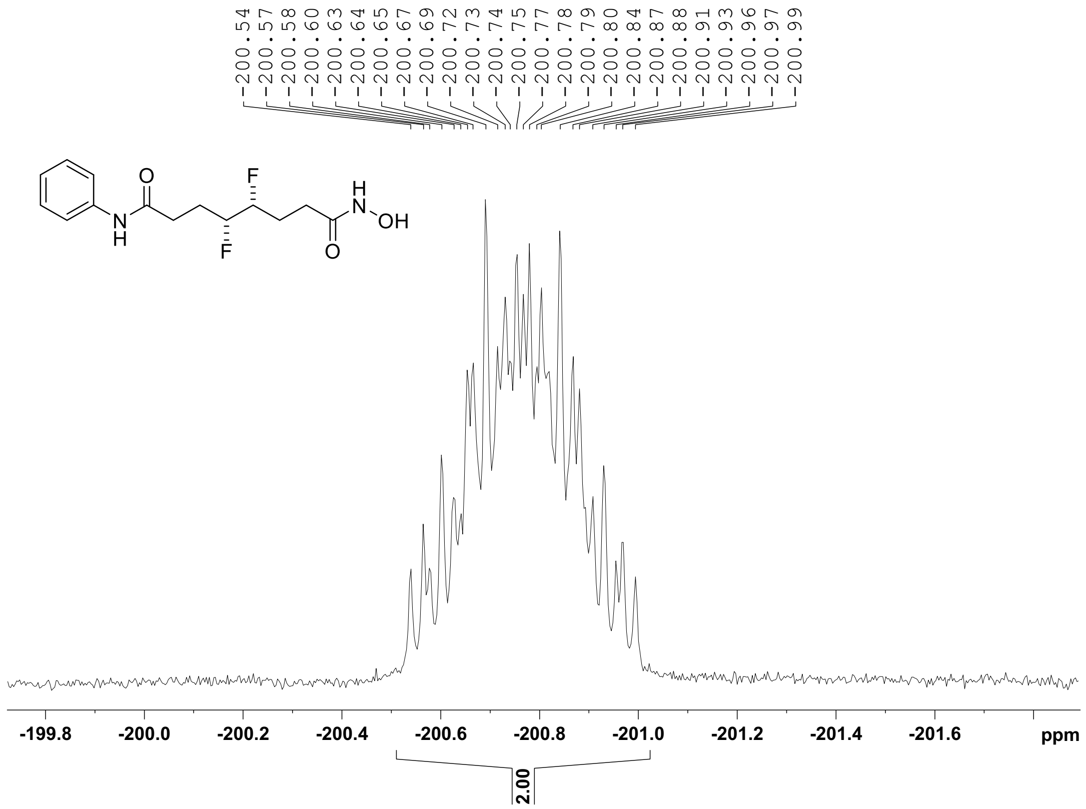

$^{19}F\{^{1}H\}$ NMR (376 MHz, MeOD) of **2**

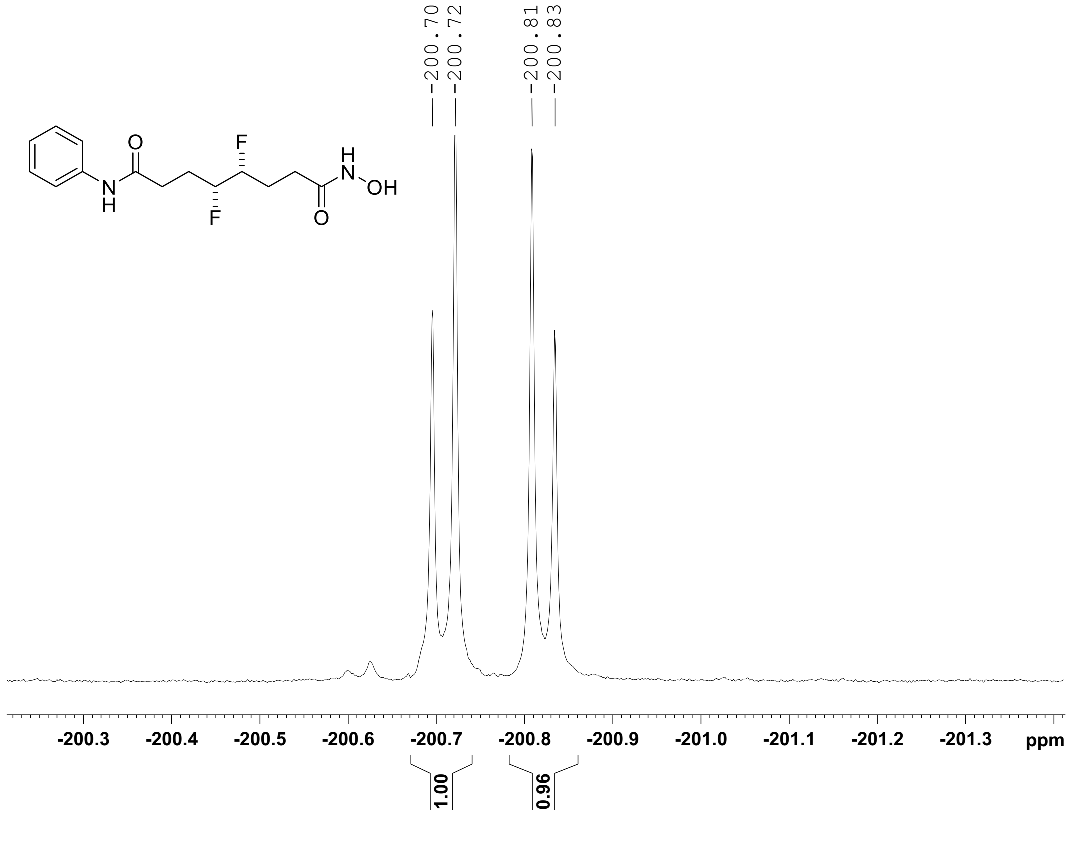

^{1}H NMR (400 MHz, MeOD) of **3**

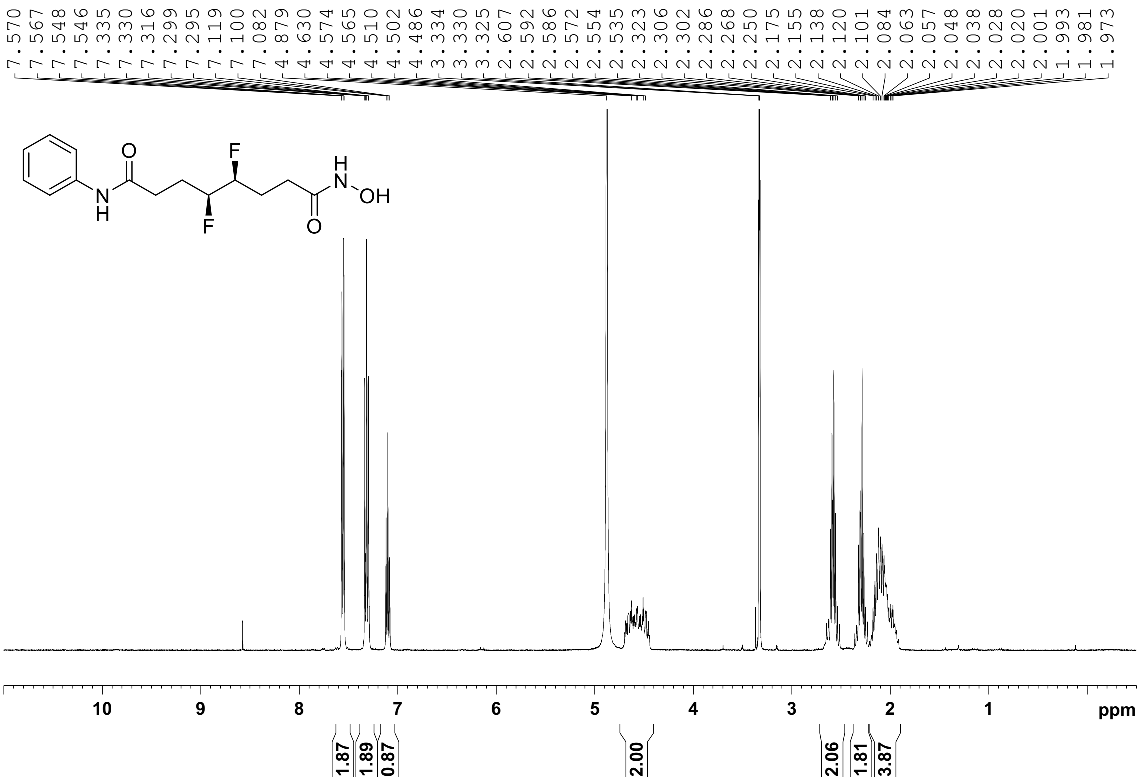

^{13}C{^{1}H} NMR (100 MHz, MeOD) of **3**

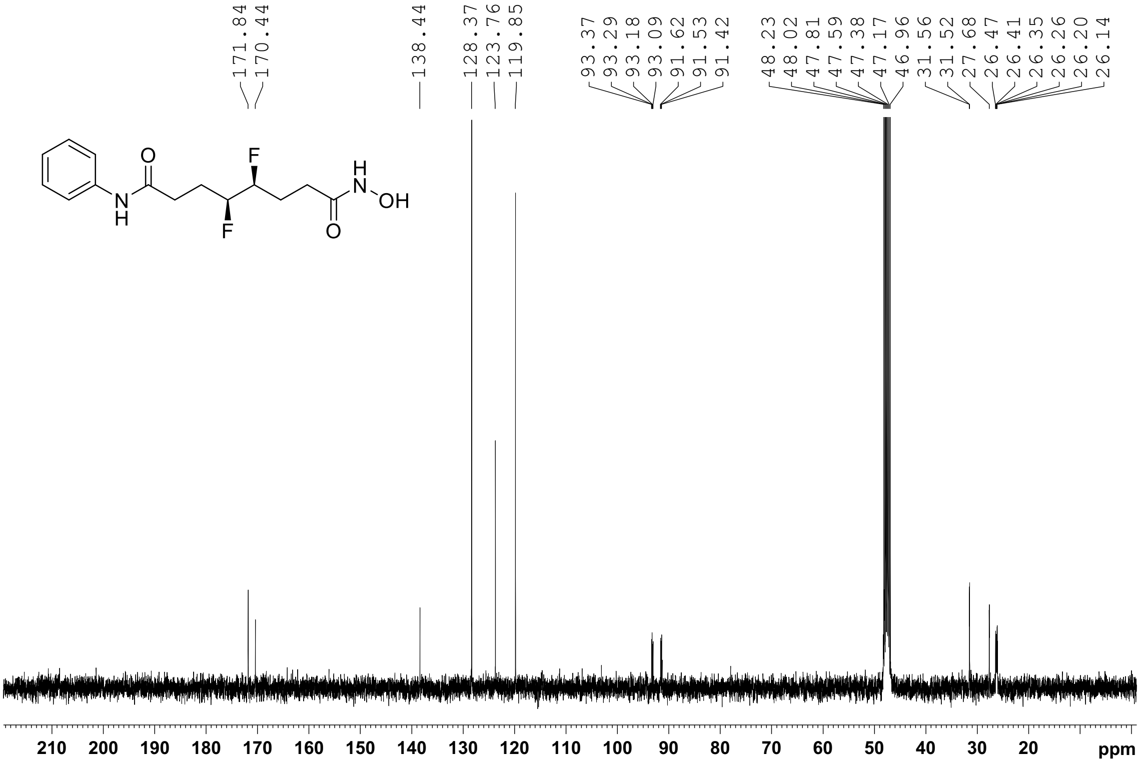

^{19}F NMR (376 MHz, MeOD) of **3**

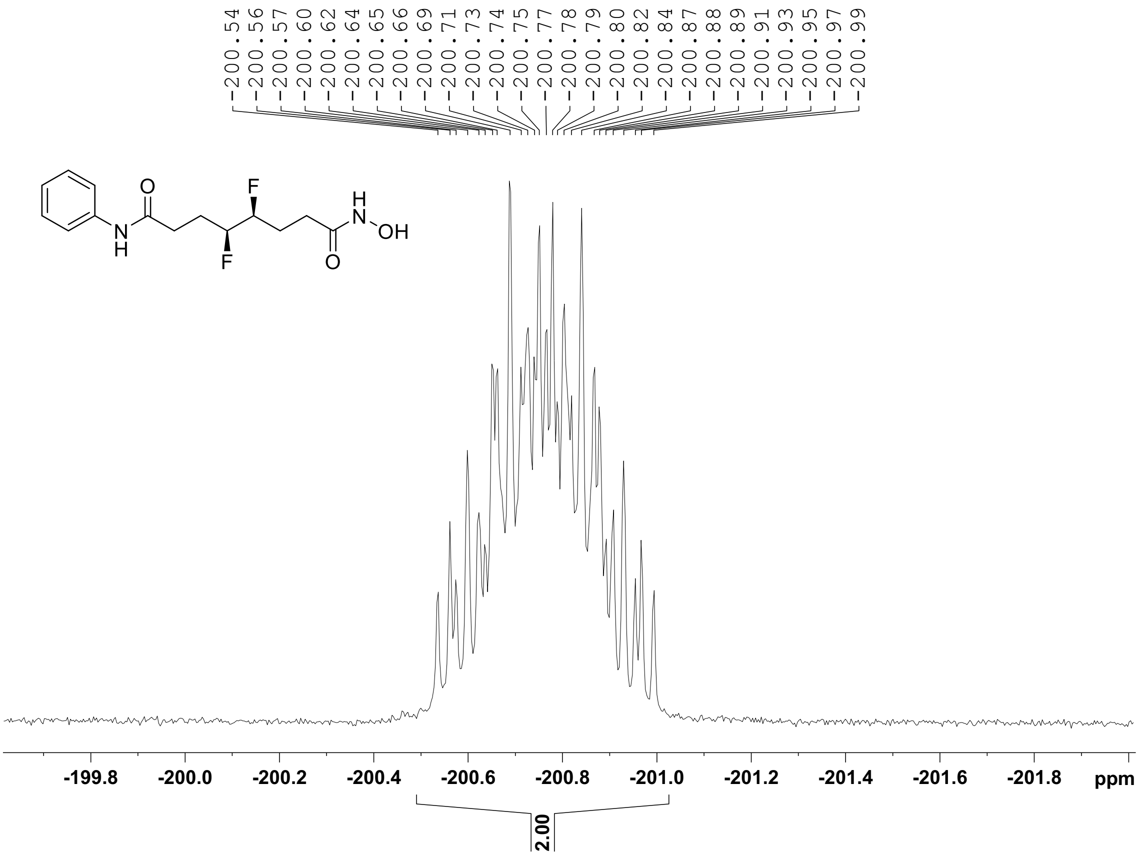

$^{19}F\{^{1}H\}$ NMR (376 MHz, MeOD) of **3**

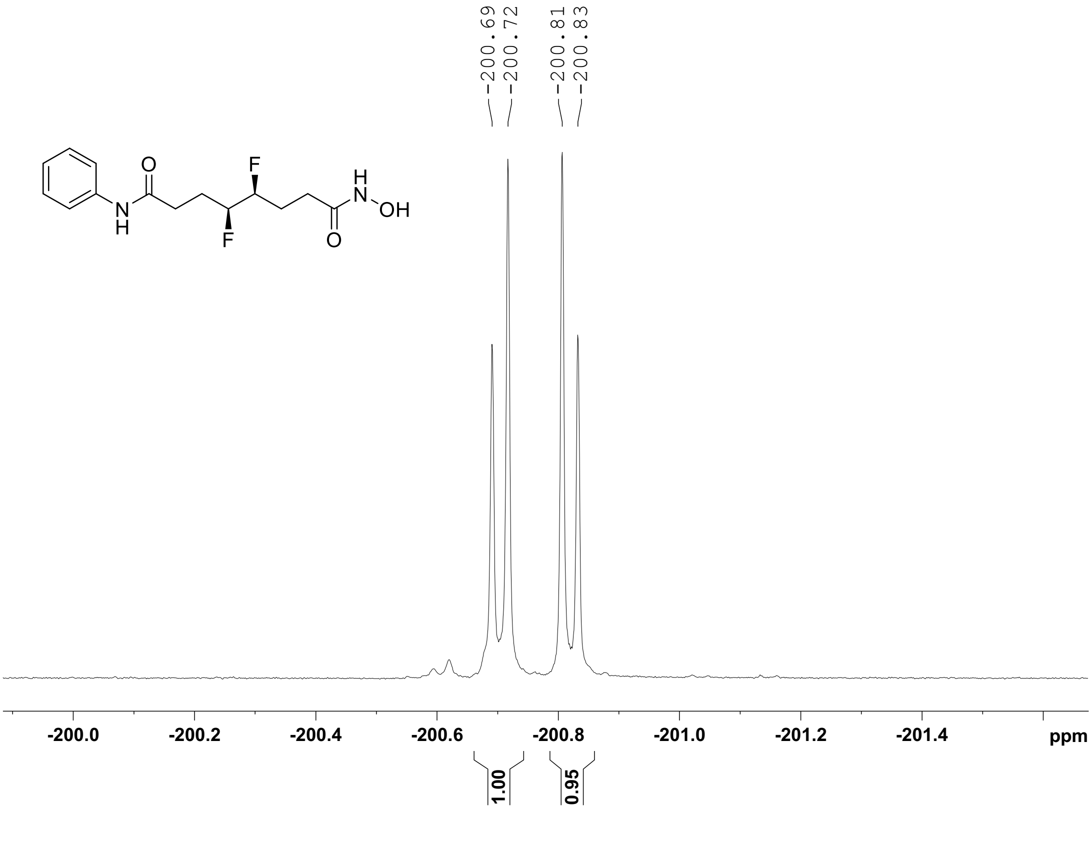

^{1}H NMR (600 MHz, MeOD) of **4**

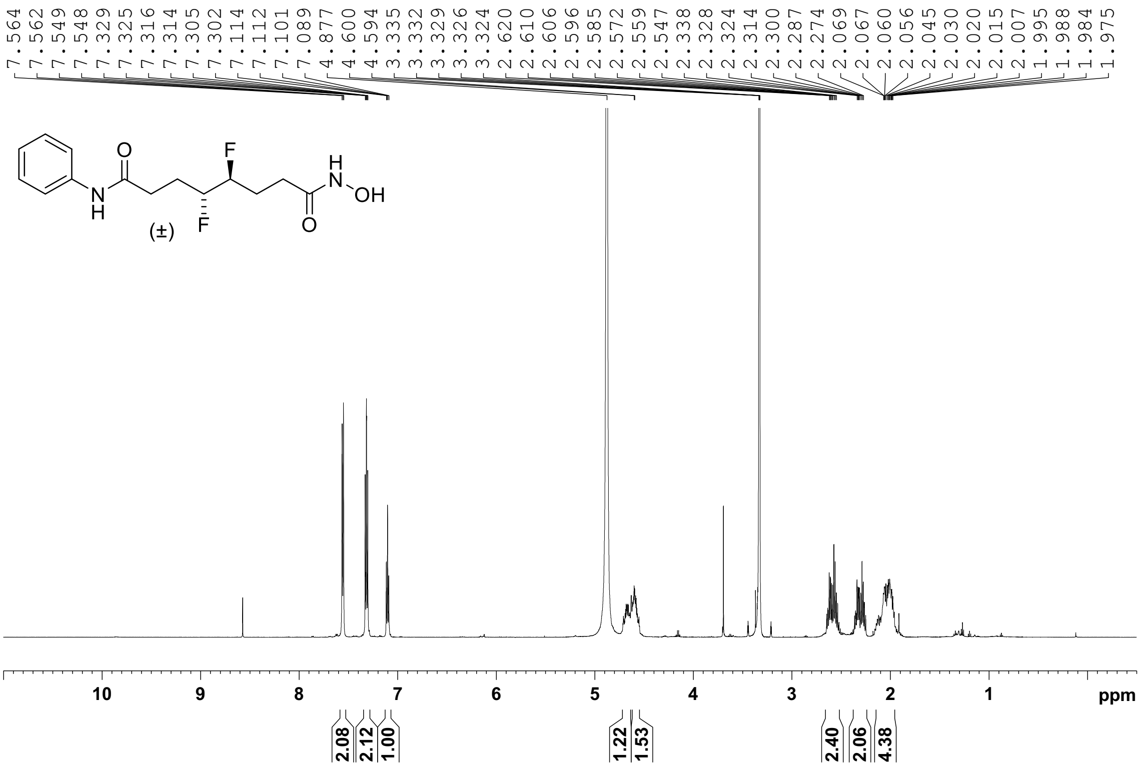

^{13}C{^{1}H} NMR (150 MHz, MeOD) of **4**

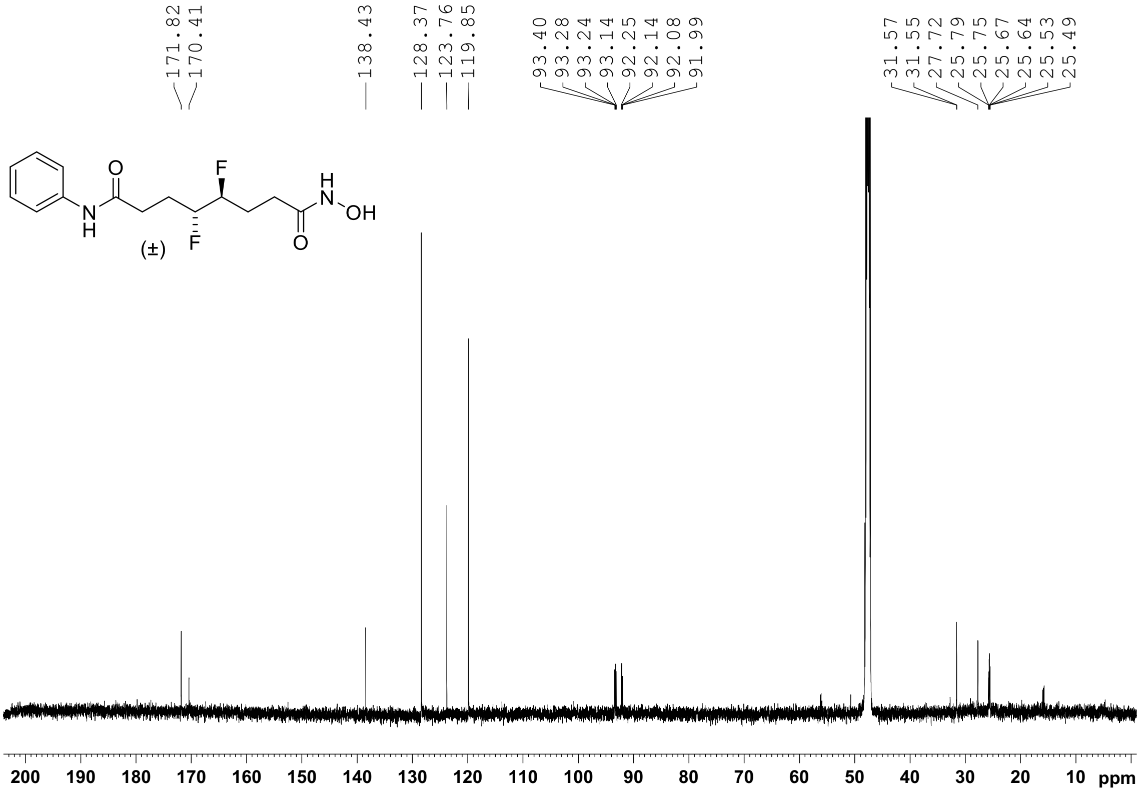

^{19}F NMR (376 MHz, MeOD) of **4**

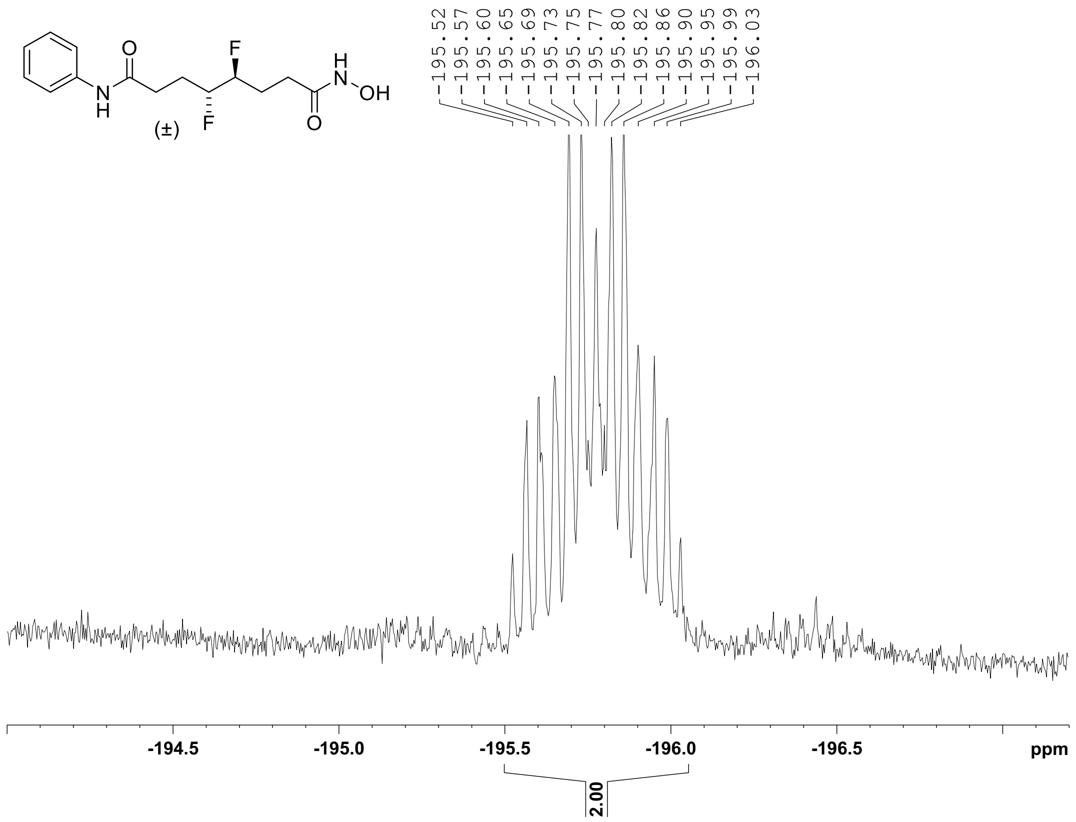

$^{19}F\{^{1}H\}$ NMR (376 MHz, MeOD) of **4**

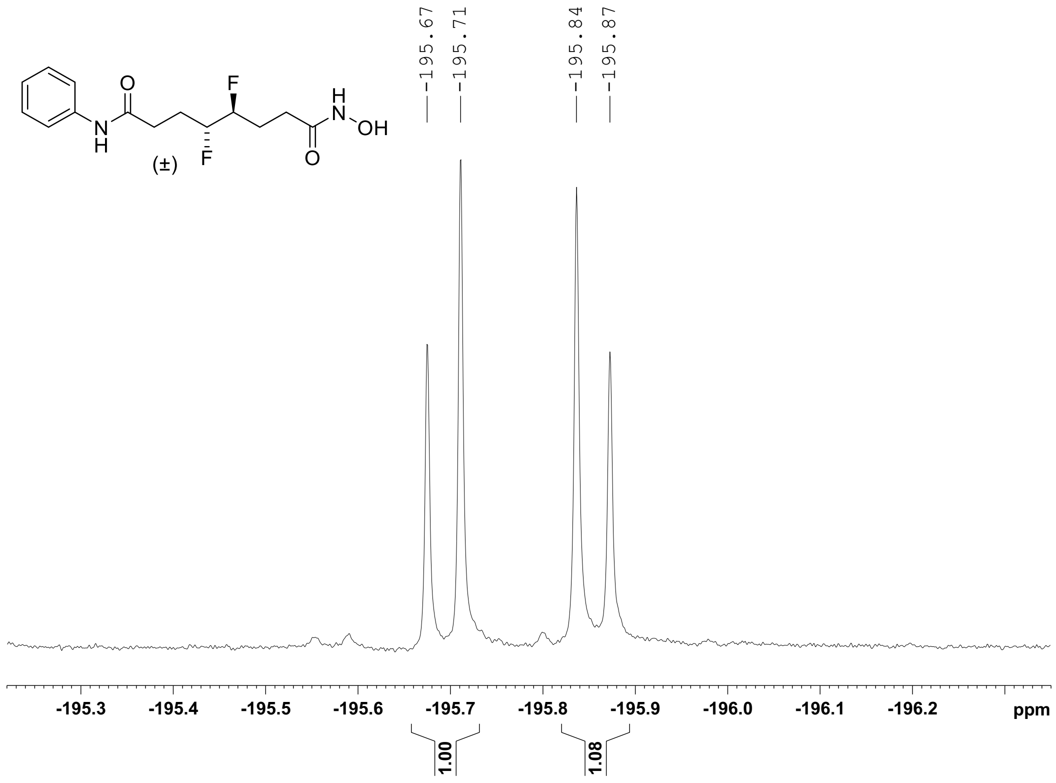

^{1}H NMR (600 MHz, MeOD) of **5**

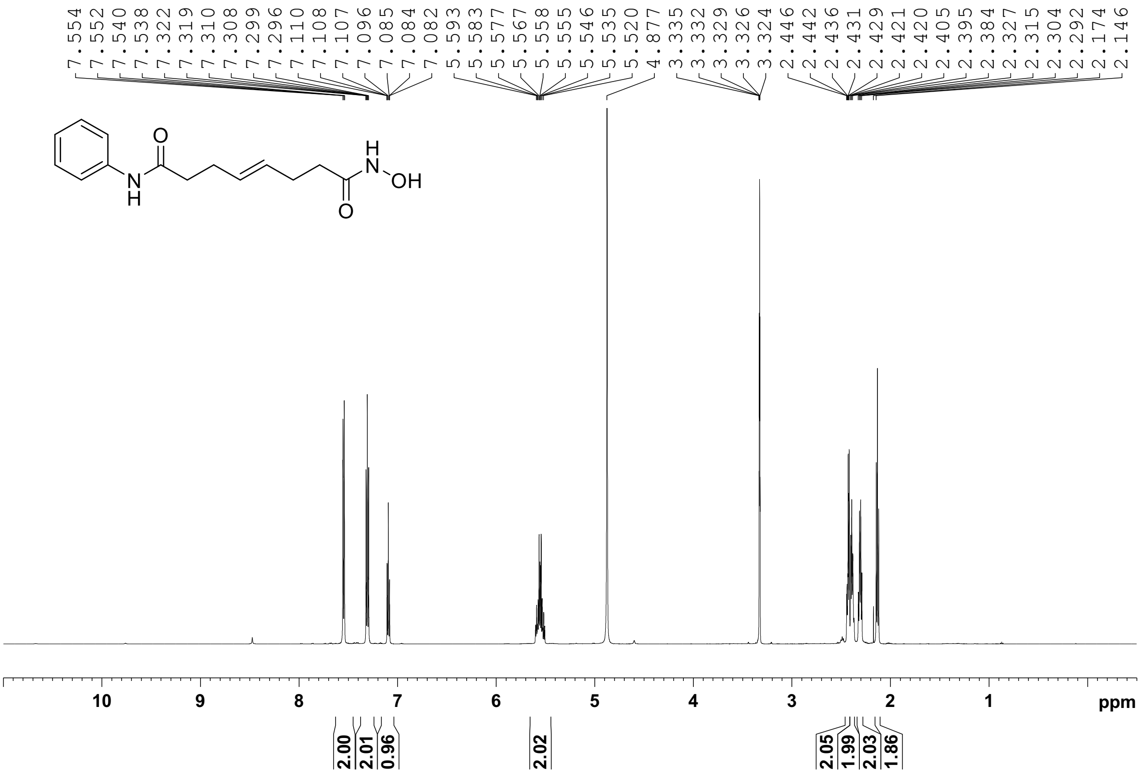

^{13}C{^{1}H} NMR (150 MHz, MeOD) of **5**

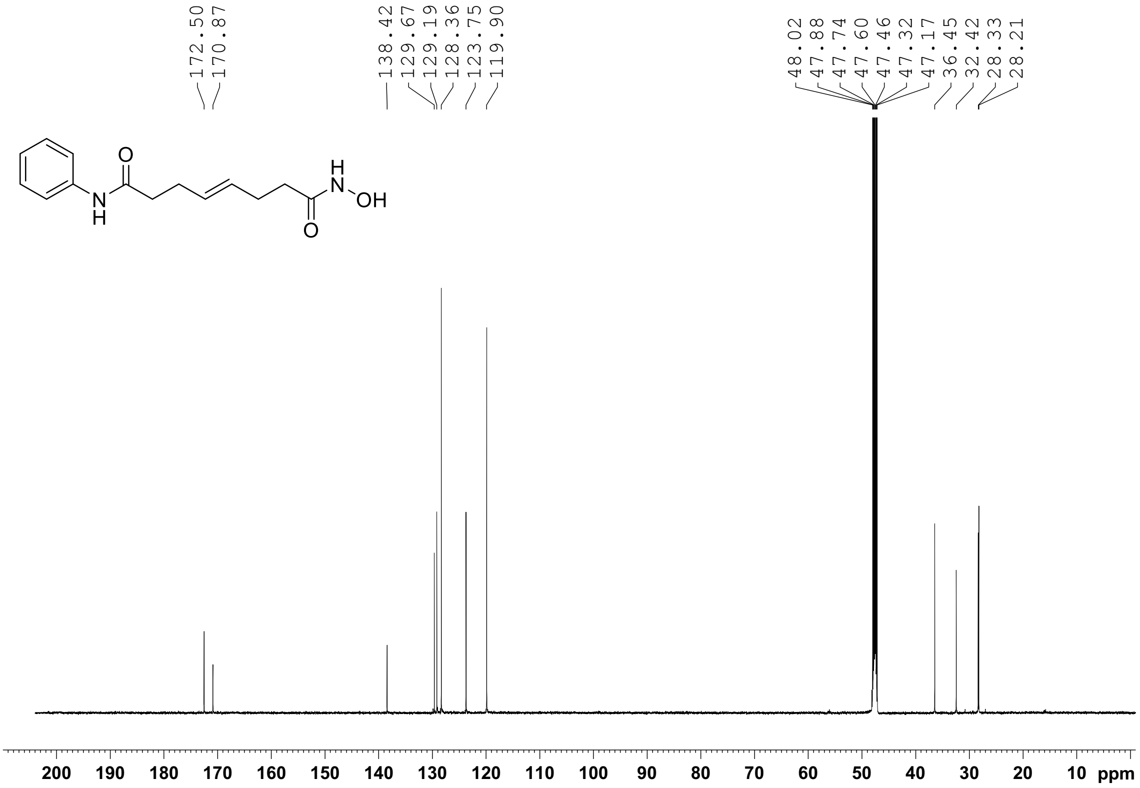

^{1}H NMR (400 MHz, MeOD) of **6**

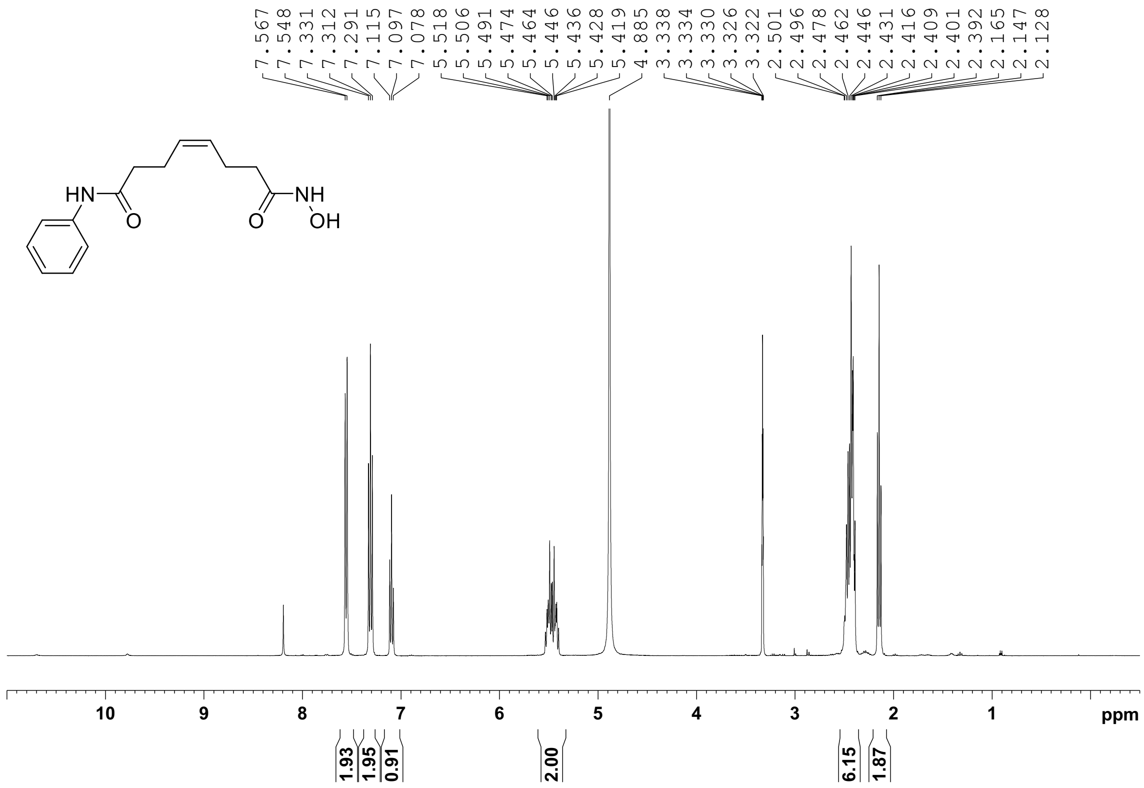

$^{13}C\{^{1}H\}$ NMR (100 MHz, MeOD) of **6**

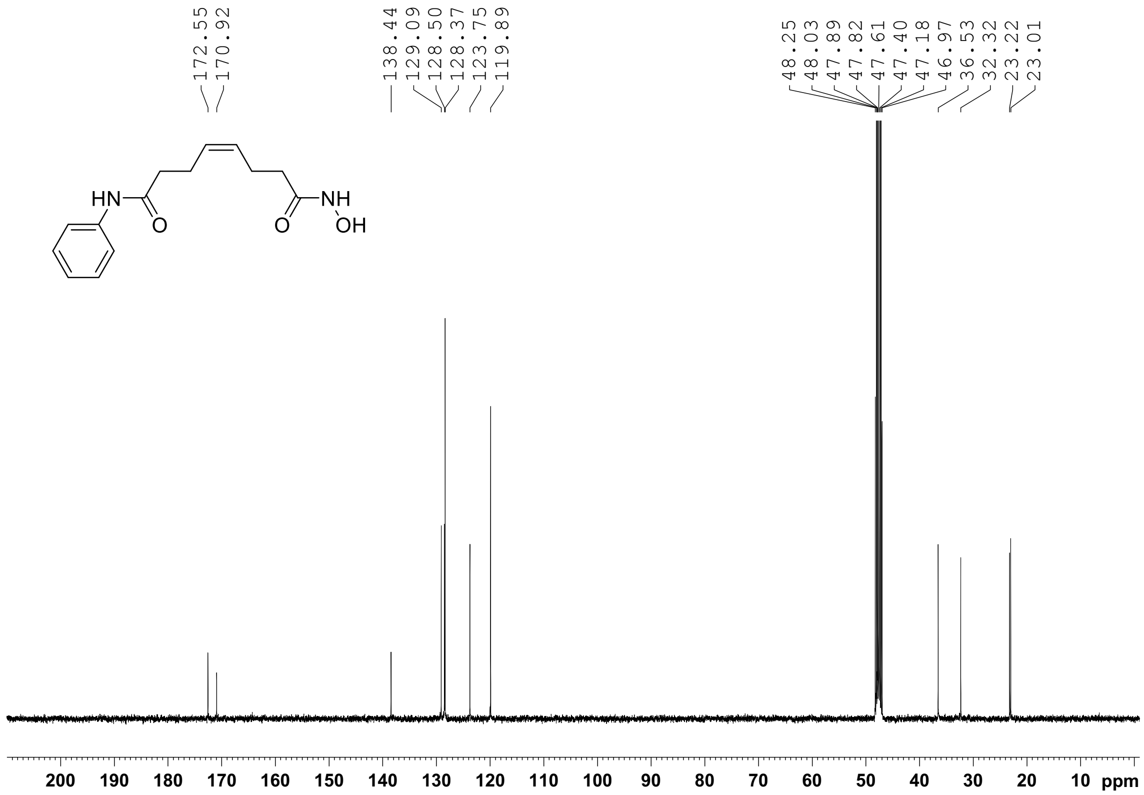

^{1}H NMR (600 MHz, DMSO-d_6) of **8**

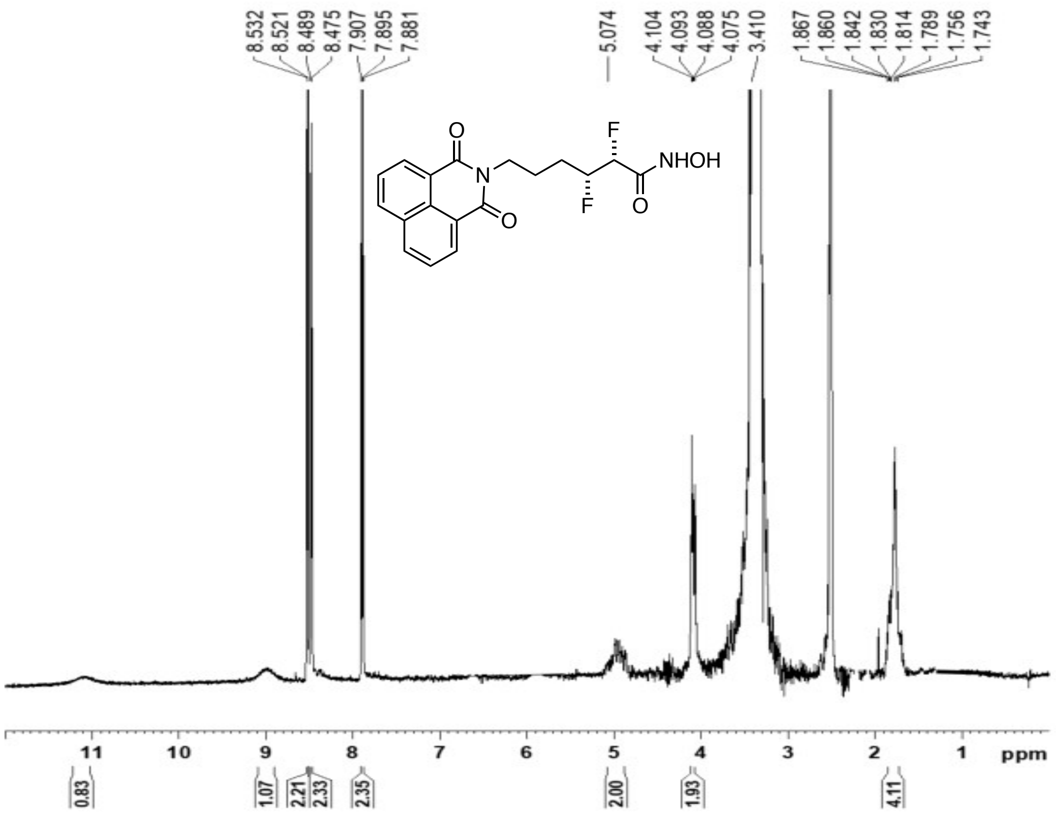

$^{13}C\{^{1}H\}$ NMR (75 MHz, DMSO-d_6) of **8**

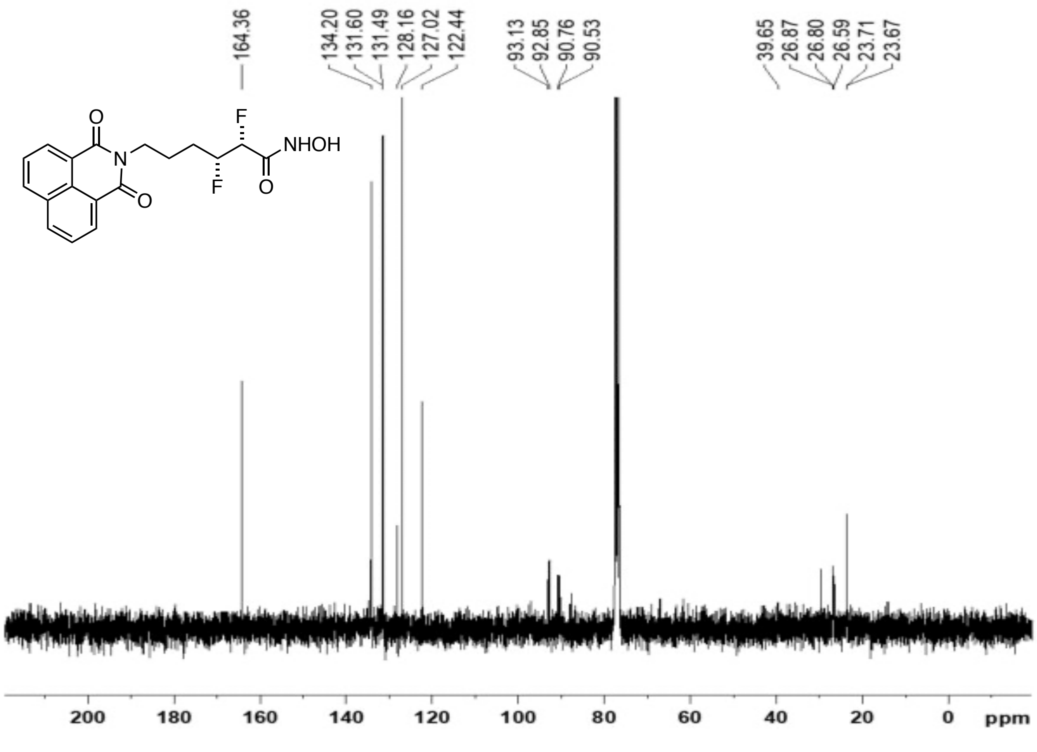

^{19}F NMR (282 MHz, DMSO-d$_6$) of **8**

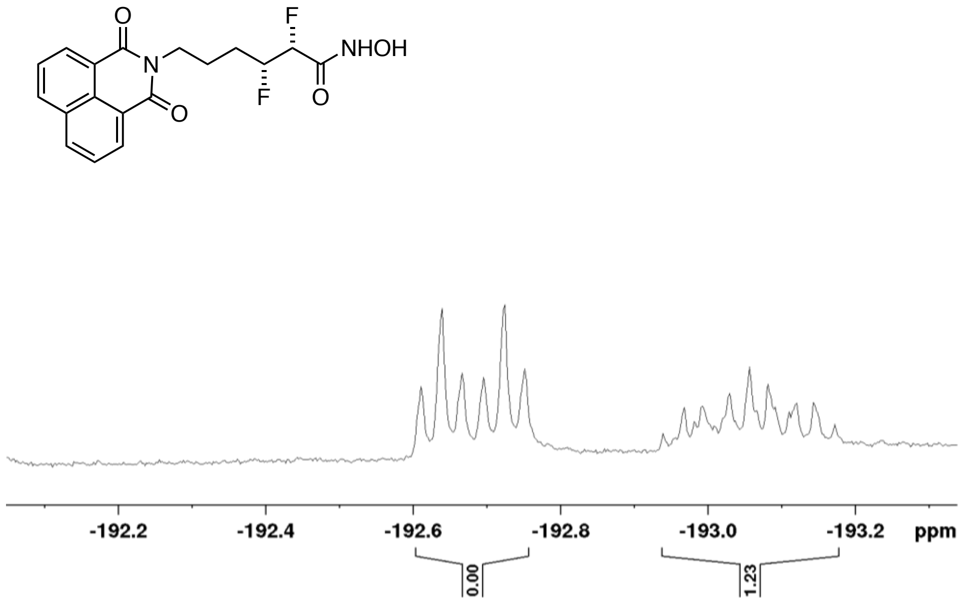

^{1}H NMR (600 MHz, DMSO-d$_6$) of **9**

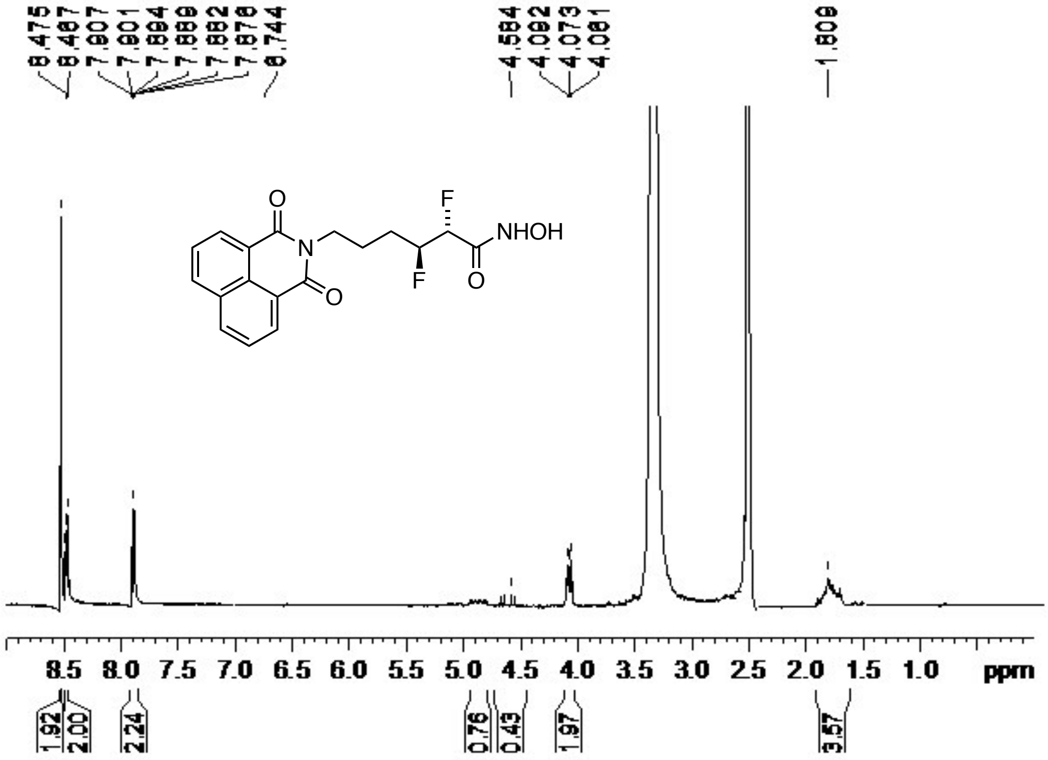

$^{13}C\{^{1}H\}$ NMR (75 MHz, DMSO-d_6) of **9**

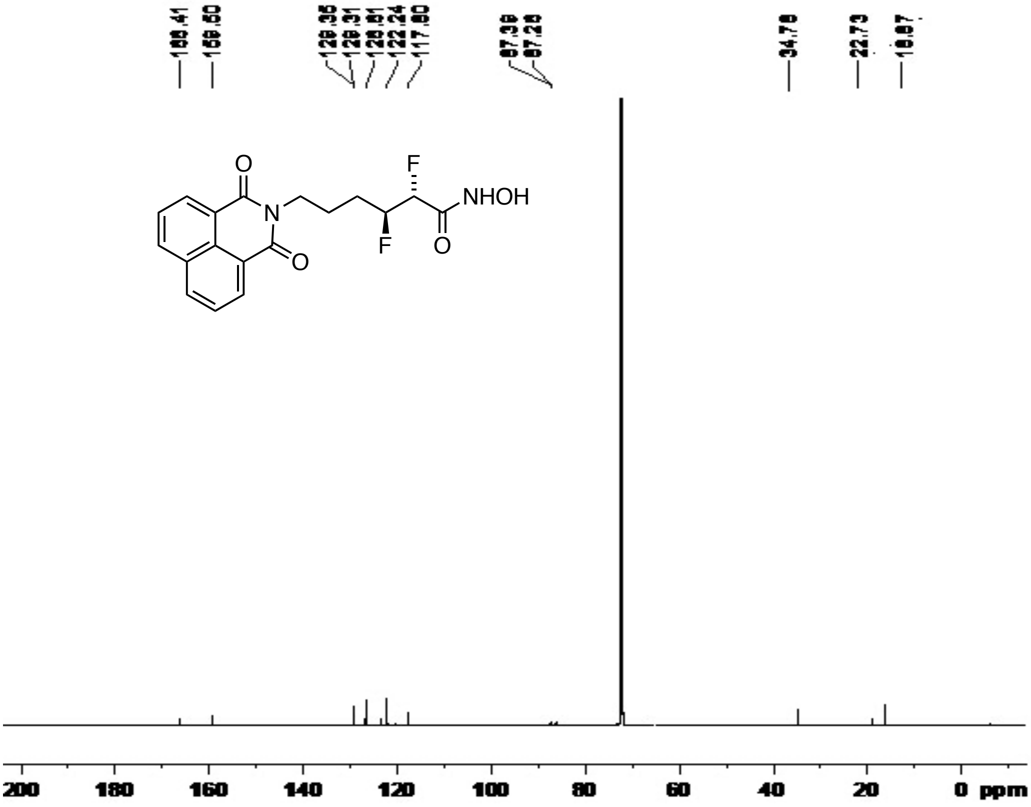

^{19}F NMR (282 MHz, DMSO-d_6) of **9**

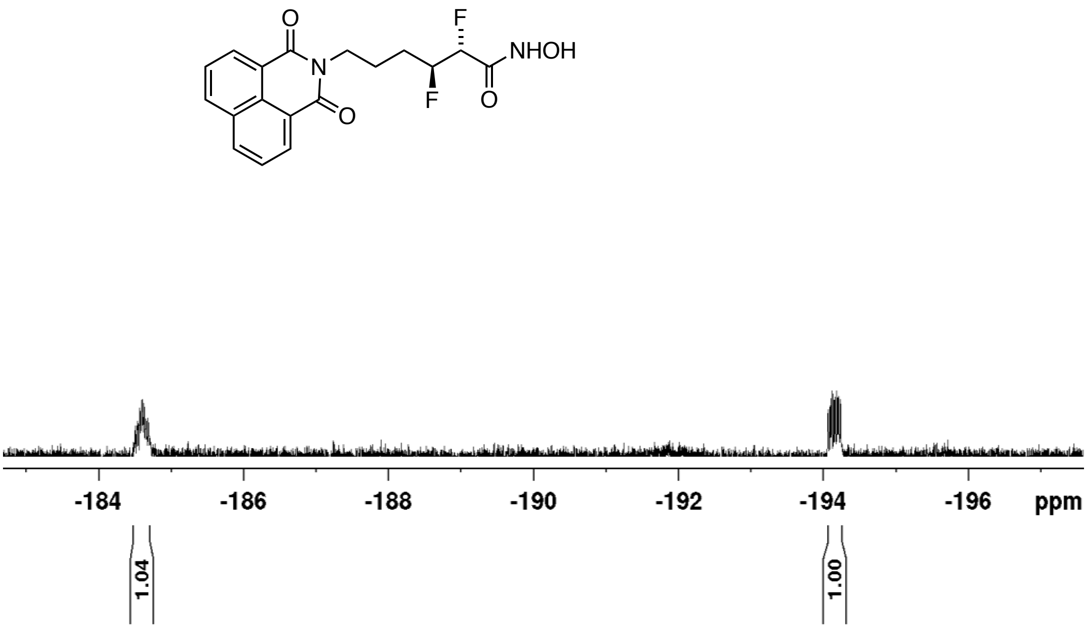

^{1}H NMR (400 MHz, $CDCl_3$) of **11**

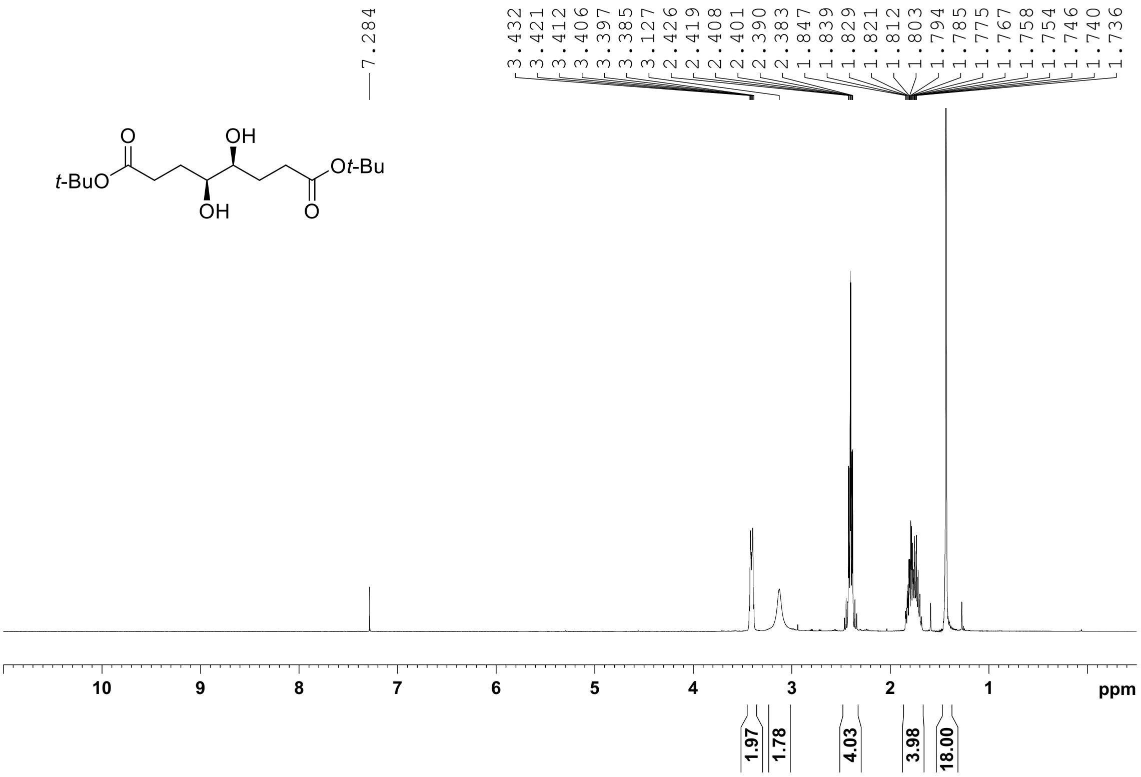

^{13}C{^{1}H} NMR (100 MHz, $CDCl_3$) of **11**

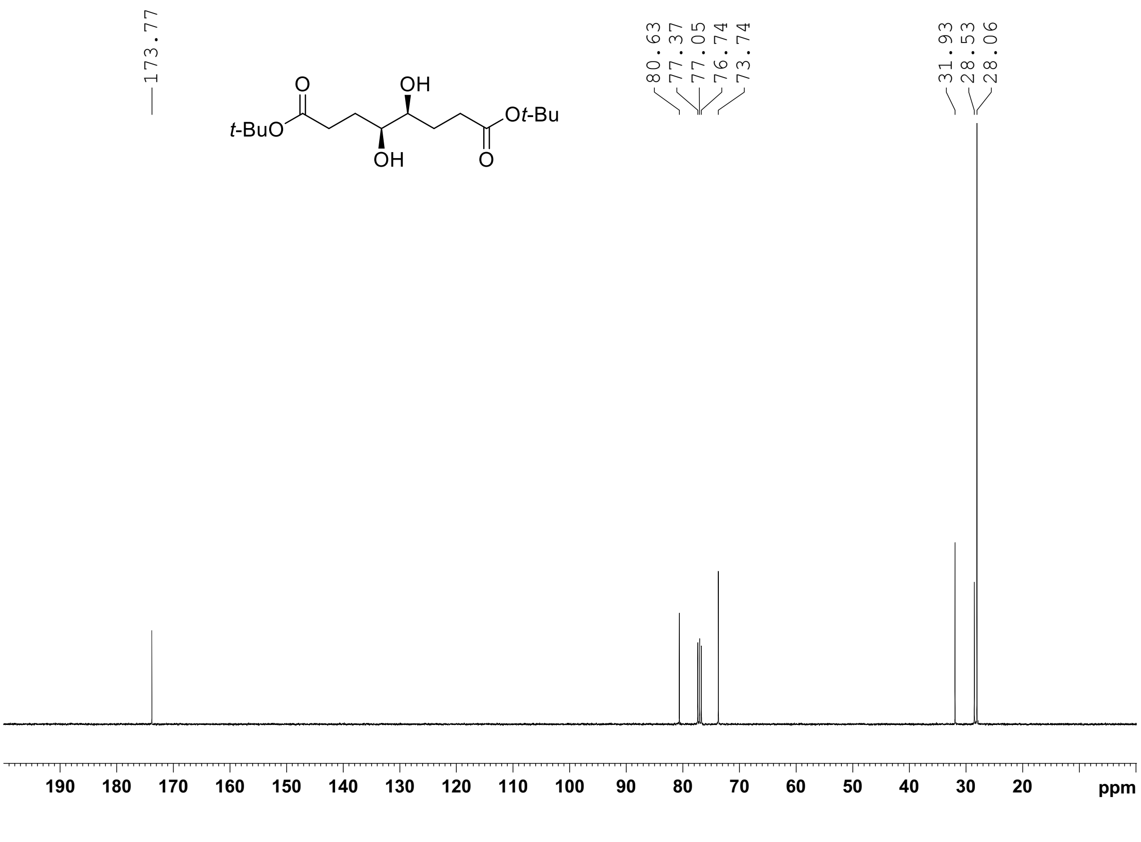

^{1}H NMR (400 MHz, $CDCl_3$) of **12**

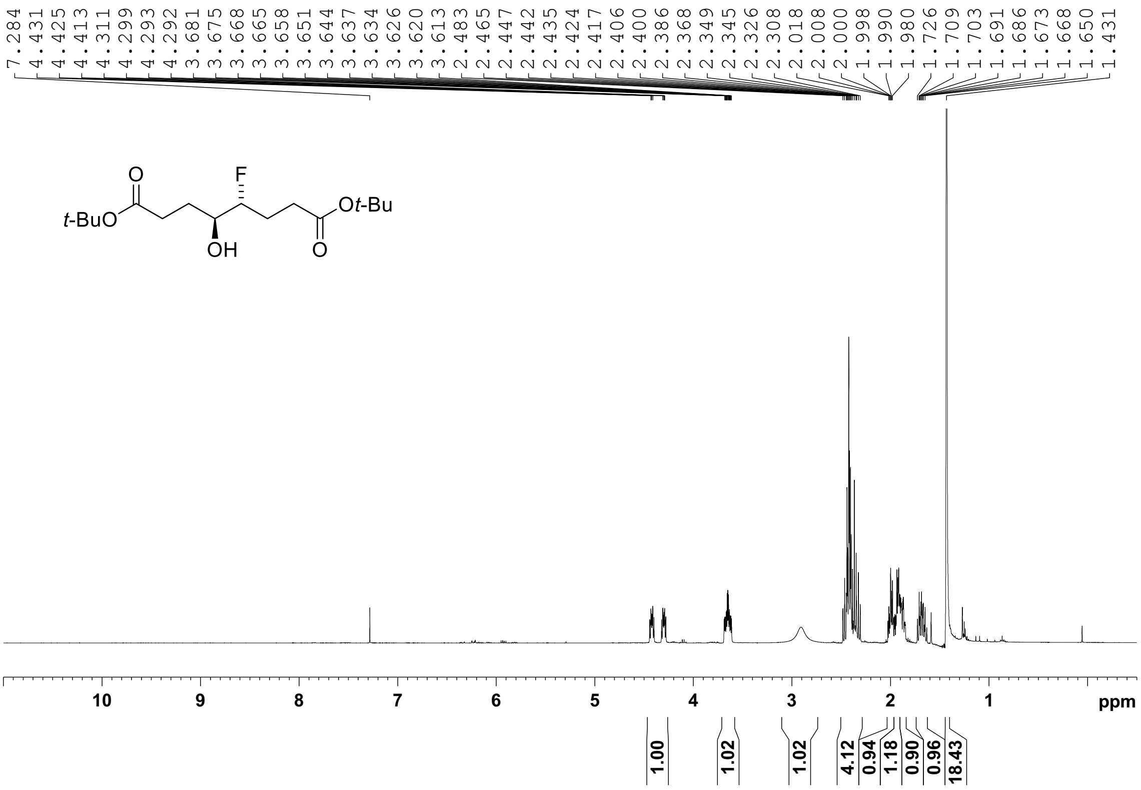

^{13}C{^{1}H} NMR (100 MHz, $CDCl_3$) of **12**

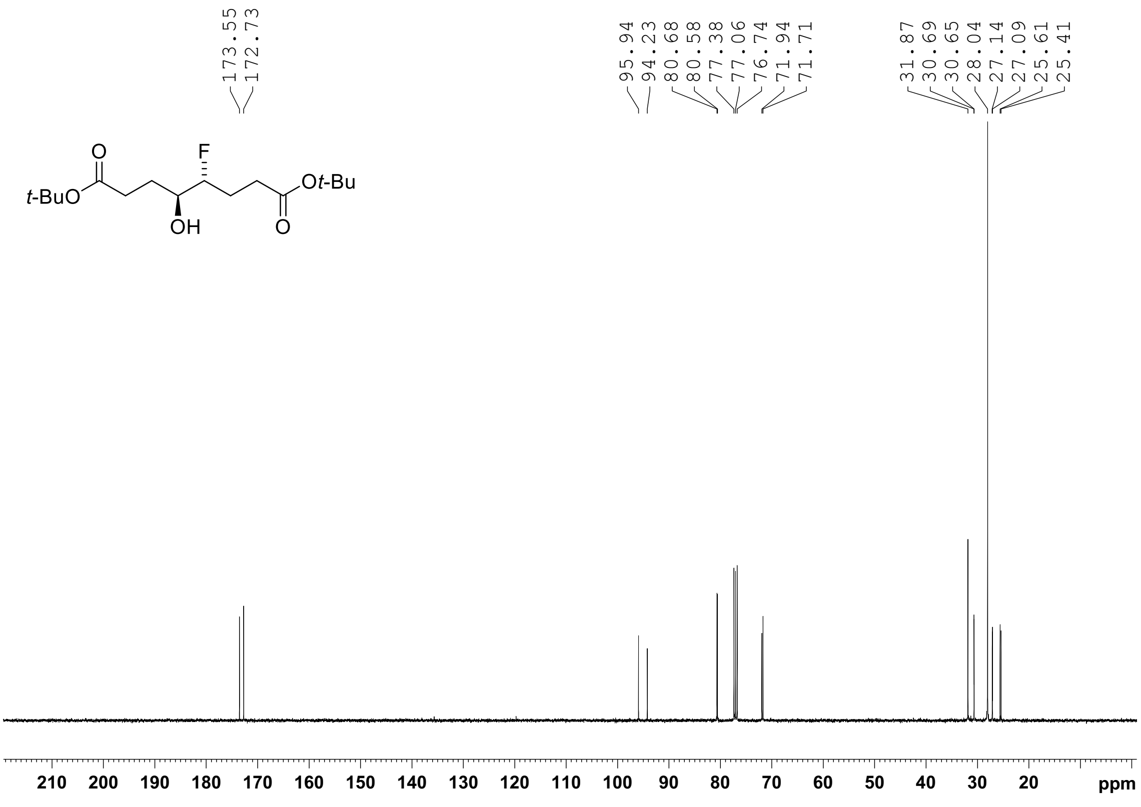

^{19}F NMR (376 MHz, $CDCl_3$) of **12**

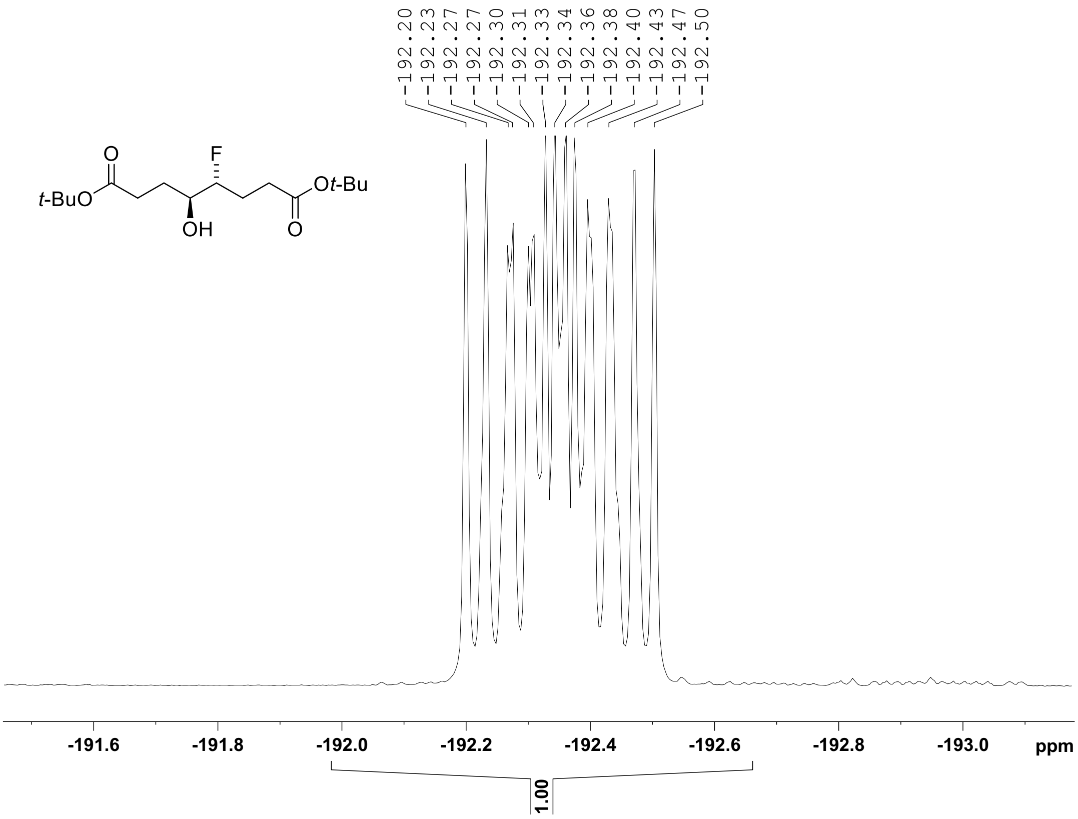

$^{19}F\{^1H\}$ NMR (376 MHz, $CDCl_3$) of **12**

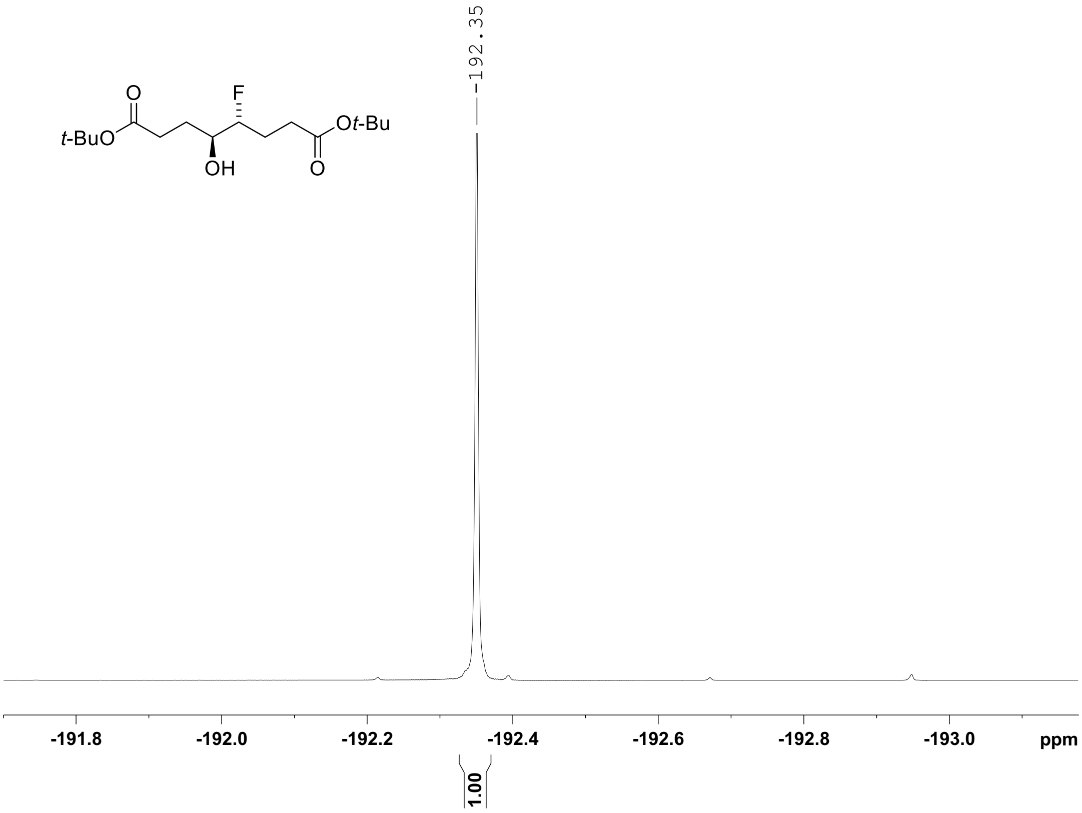

^{1}H NMR (400 MHz, $CDCl_3$) of **13**

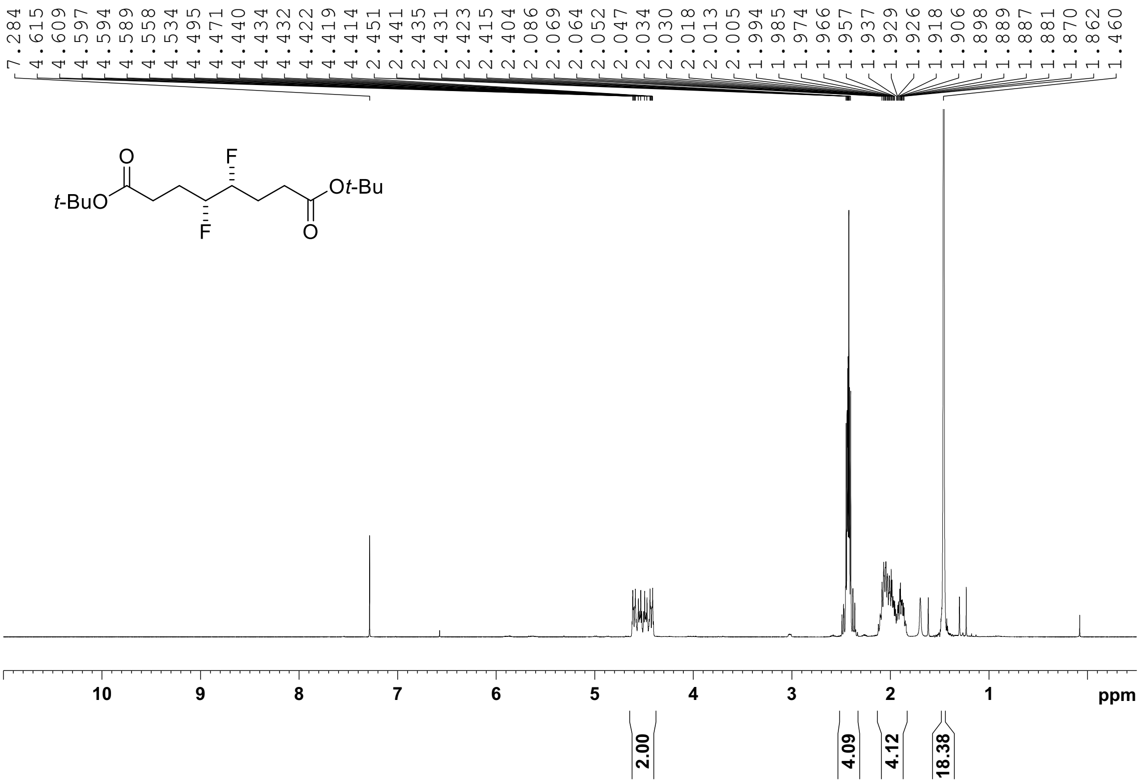

$^{13}C\{^{1}H\}$ NMR (100 MHz, $CDCl_3$) of **13**

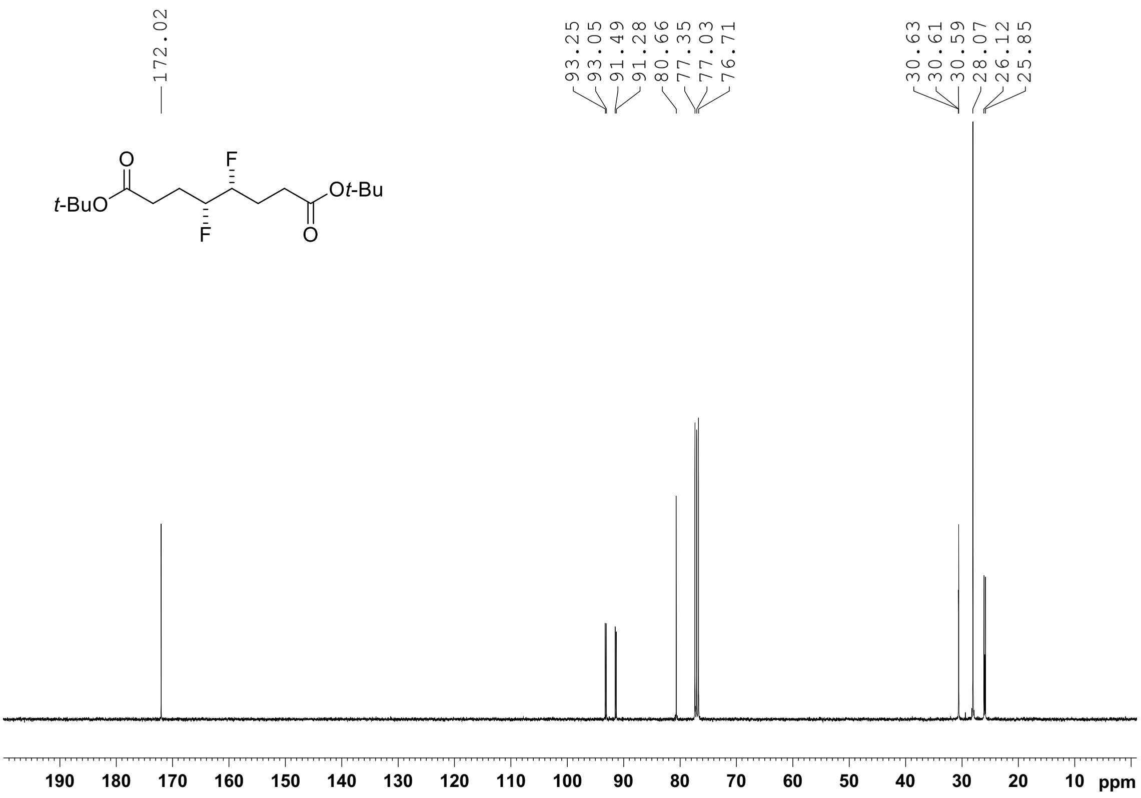

^{19}F NMR (376 MHz, $CDCl_3$) of **13**

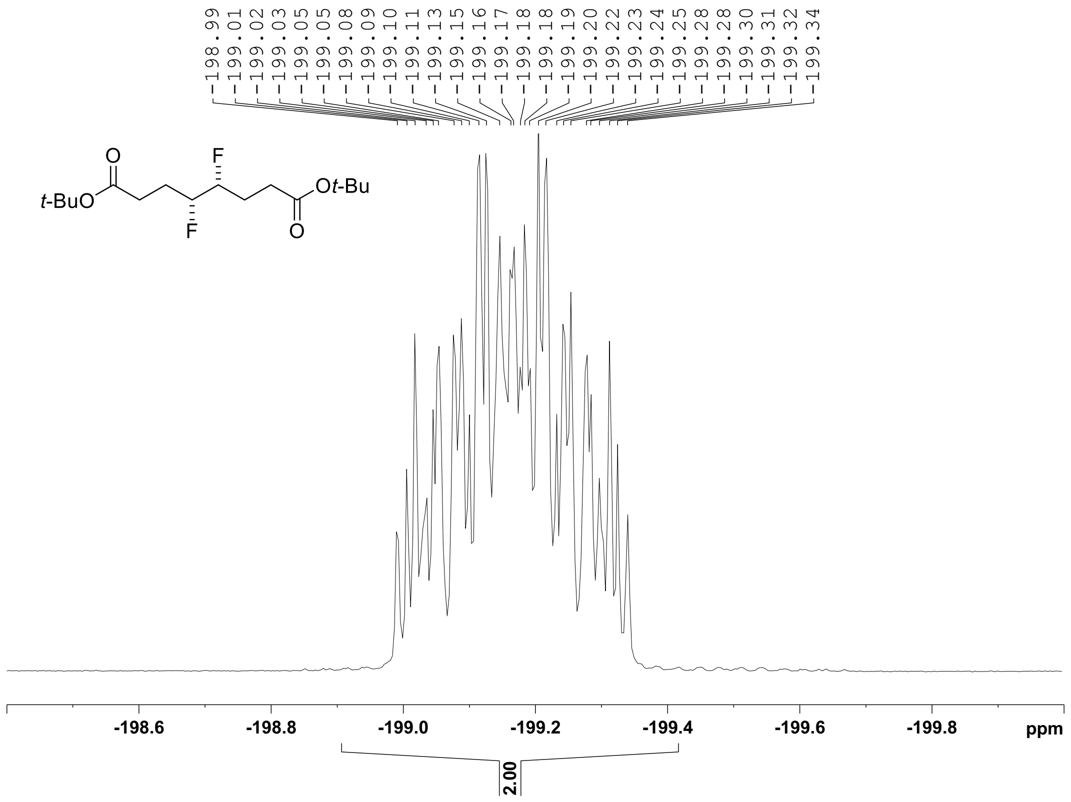

^{19}F{^{1}H} NMR (376 MHz, $CDCl_3$) of **13**

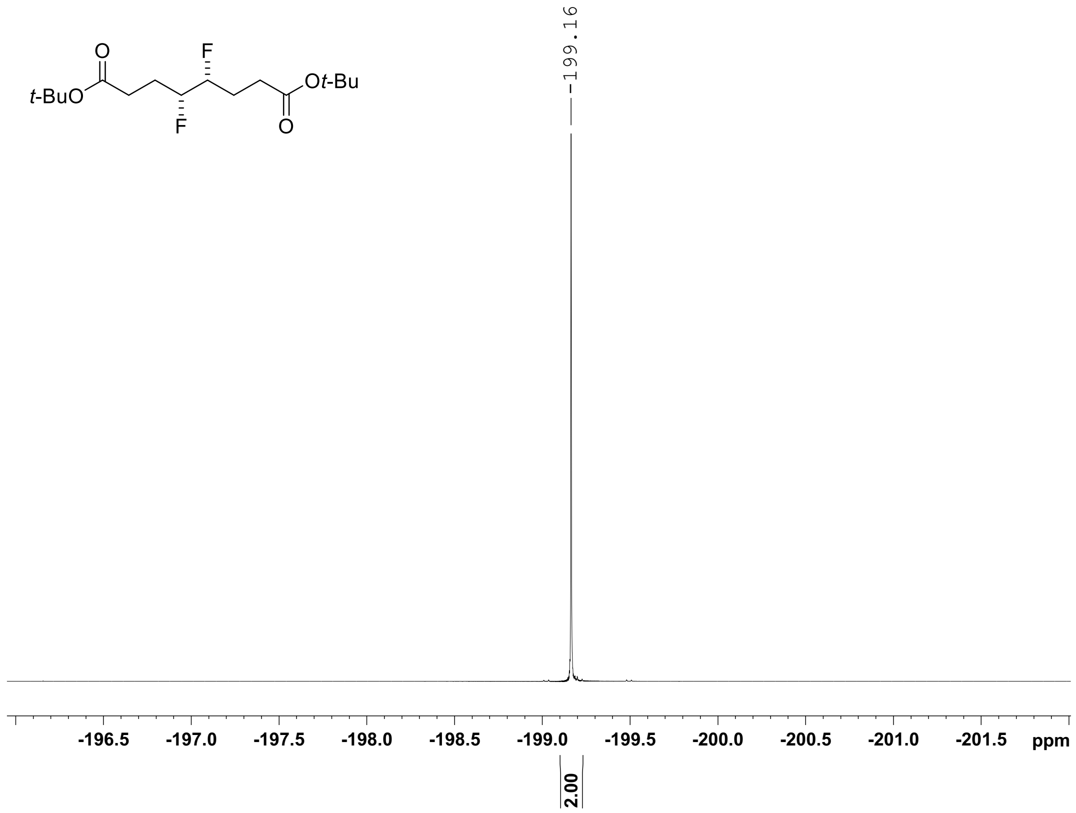

^{1}H NMR (600 MHz, MeOD) of **14**

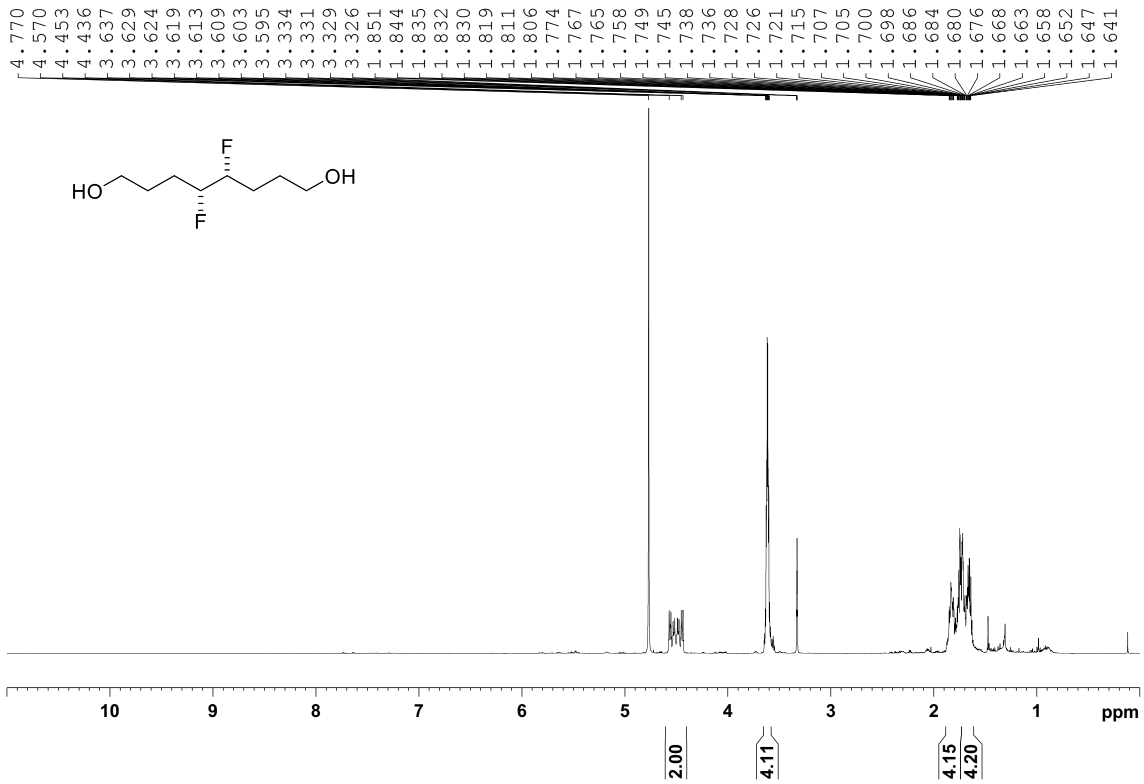

^{13}C{^{1}H} NMR (150 MHz, MeOD) of **14**

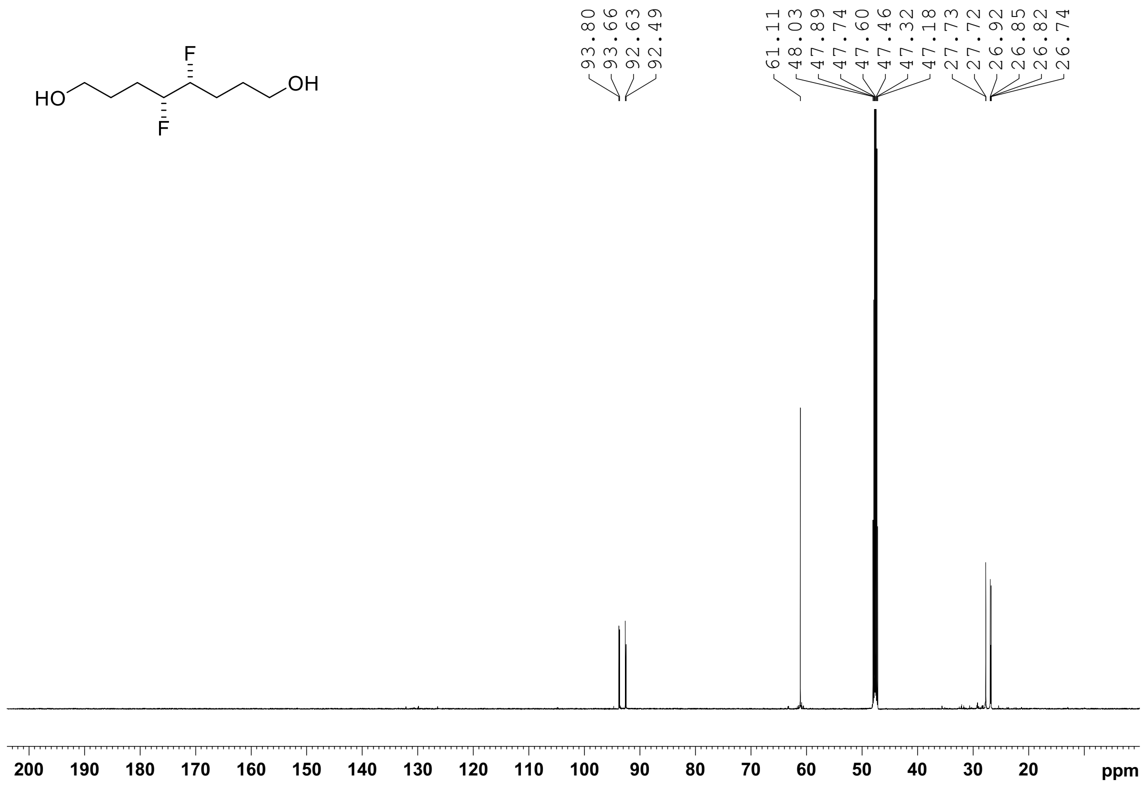

^{19}F NMR (376 MHz, MeOD) of **14**

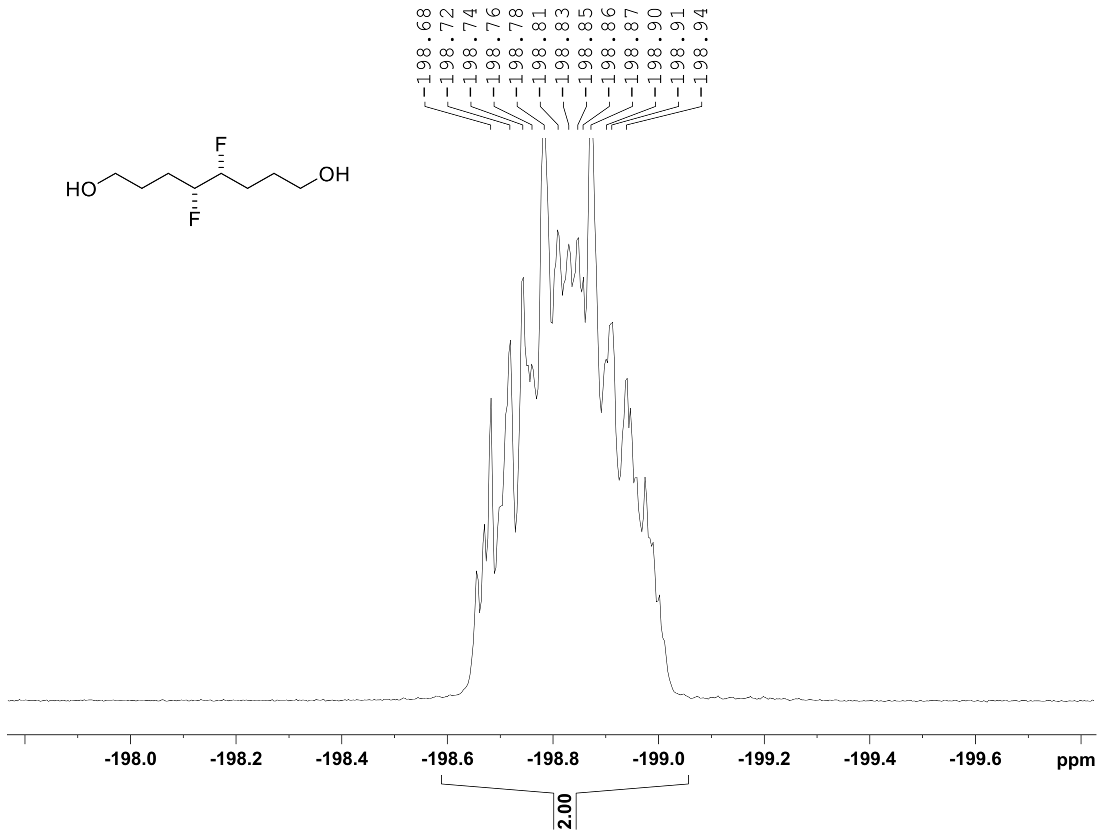

$^{19}F\{^{1}H\}$ NMR (376 MHz, MeOD) of **14**

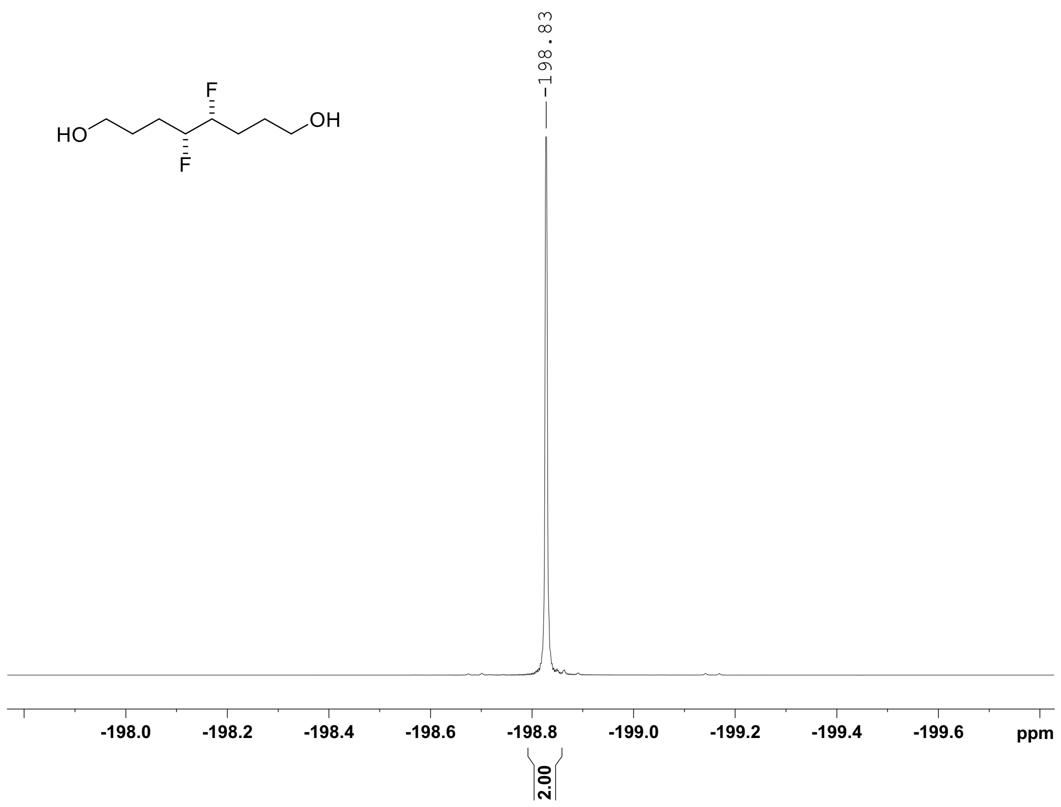

^{1}H NMR (400 MHz, MeOD) of **15**

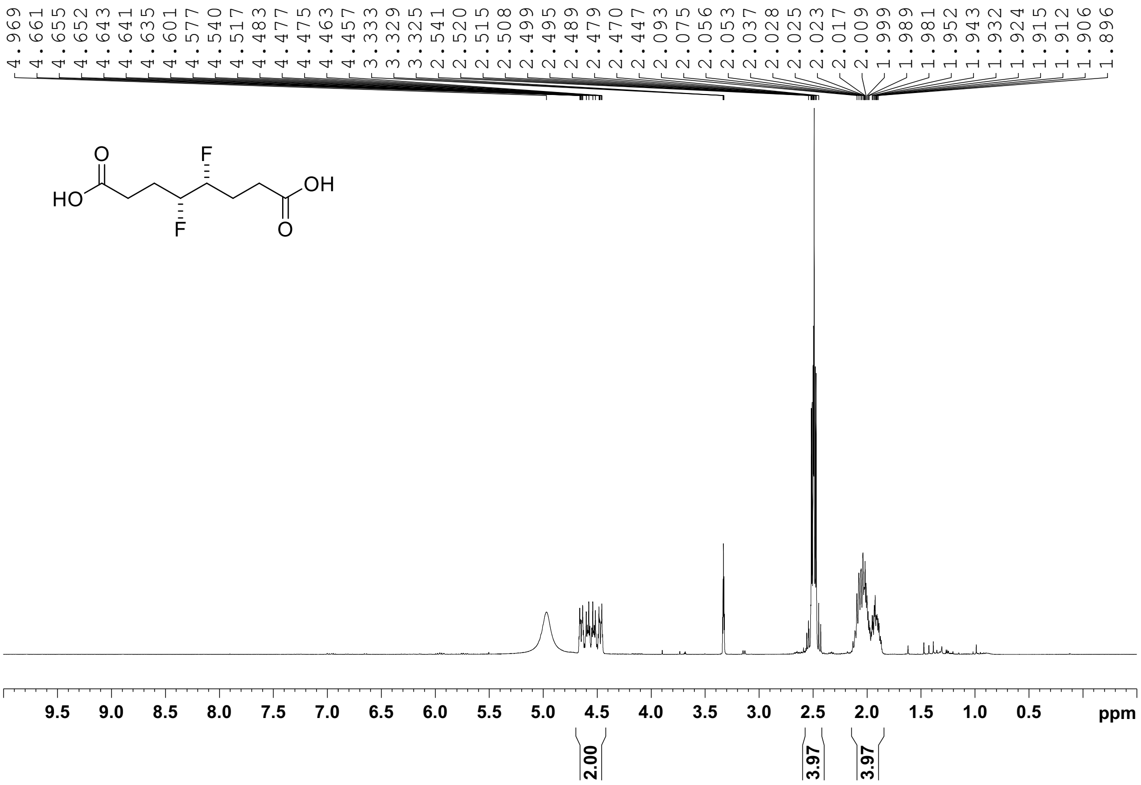

^{13}C{^{1}H} NMR (100 MHz, MeOD) of **15**

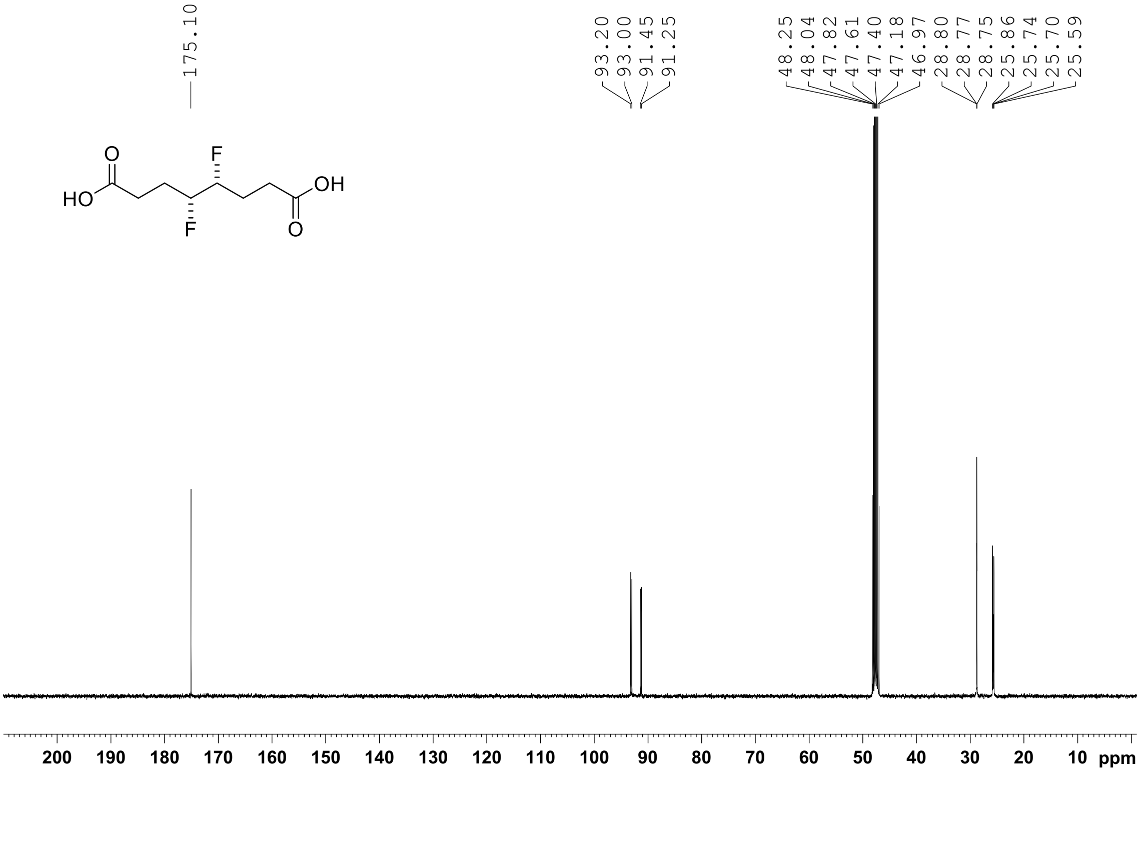

^{19}F NMR (376 MHz, MeOD) of **15**

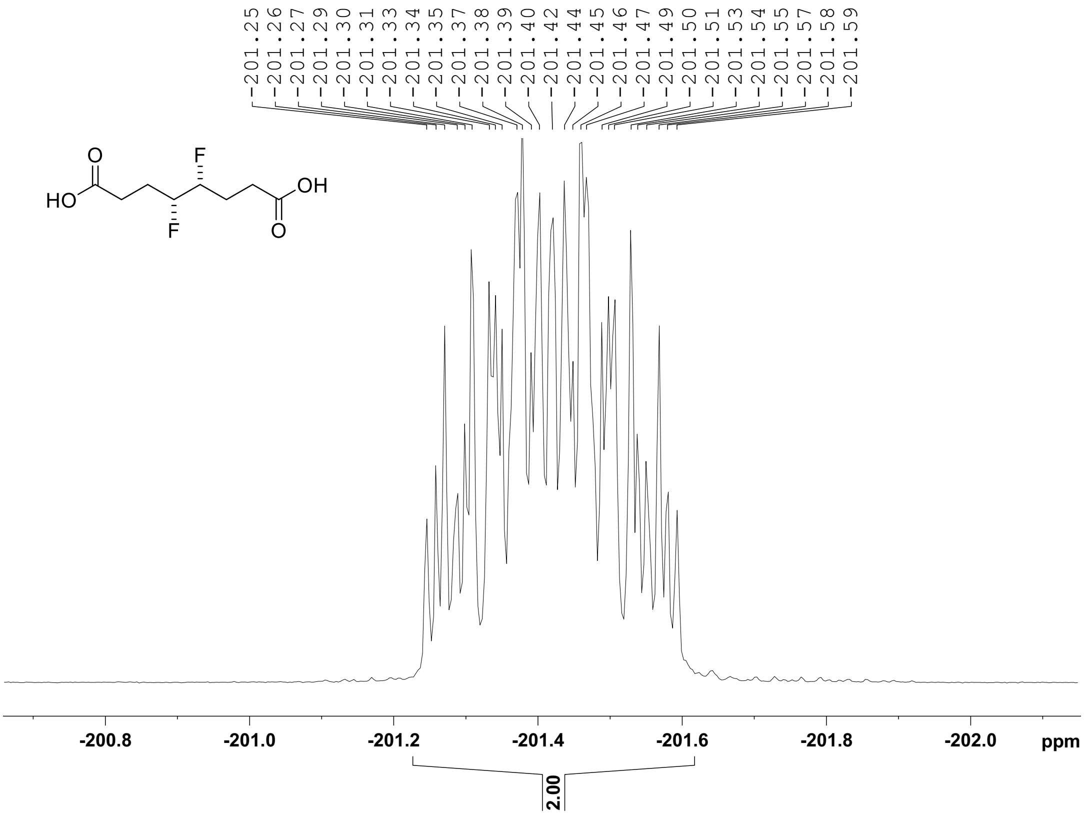

^{19}F{^{1}H} NMR (376 MHz, MeOD) of **15**

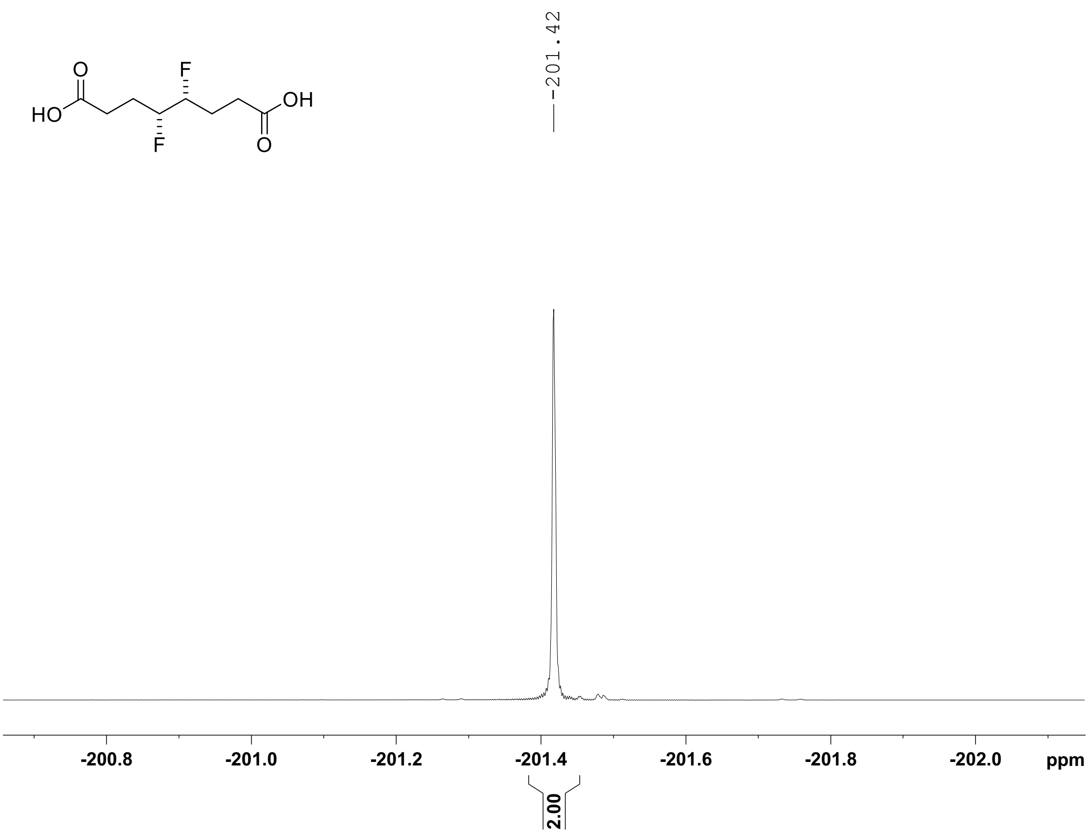

^{1}H NMR (400 MHz, $CDCl_3$) of **17**

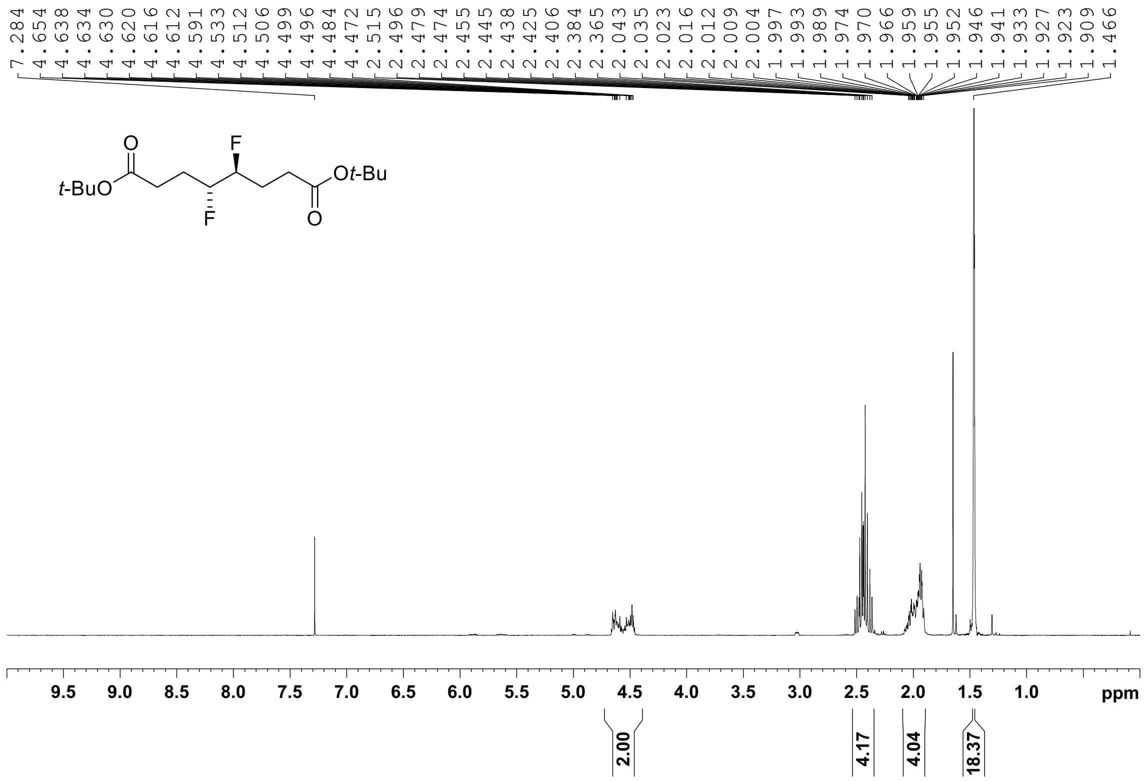

^{13}C{^{1}H} NMR (100 MHz, $CDCl_3$) of **17**

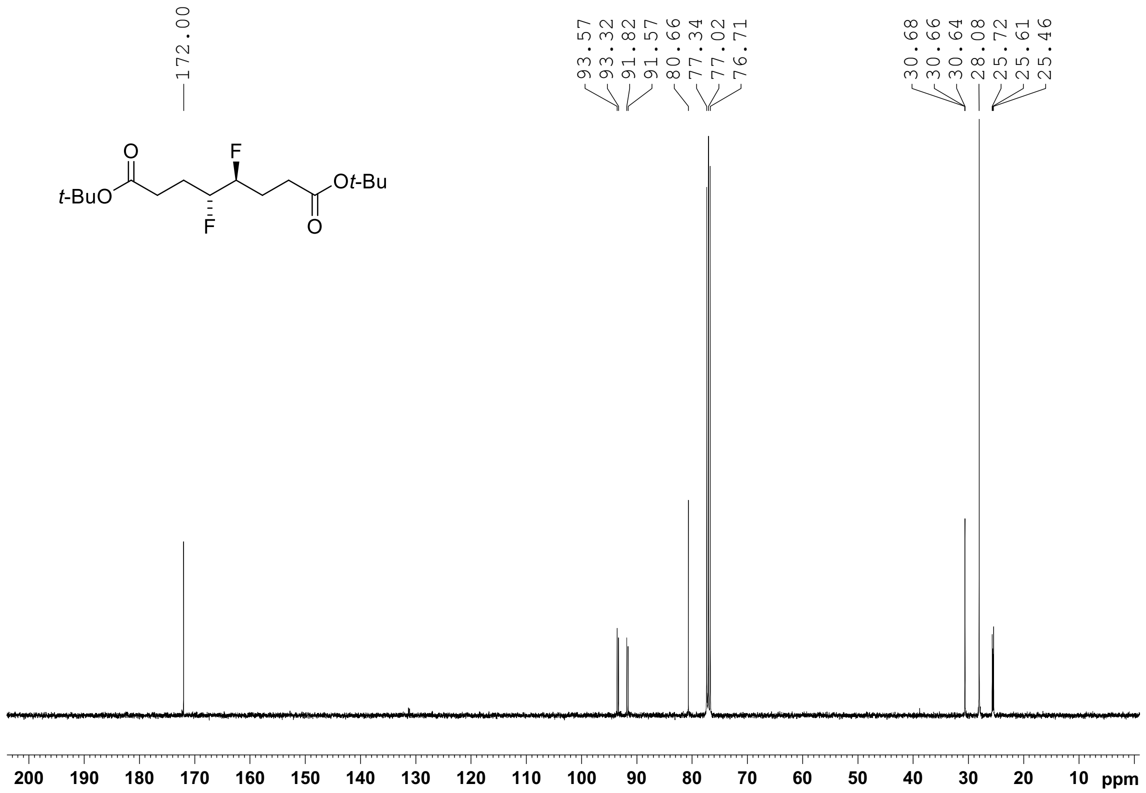

^{19}F NMR (376 MHz, CDCl$_3$) of **17**

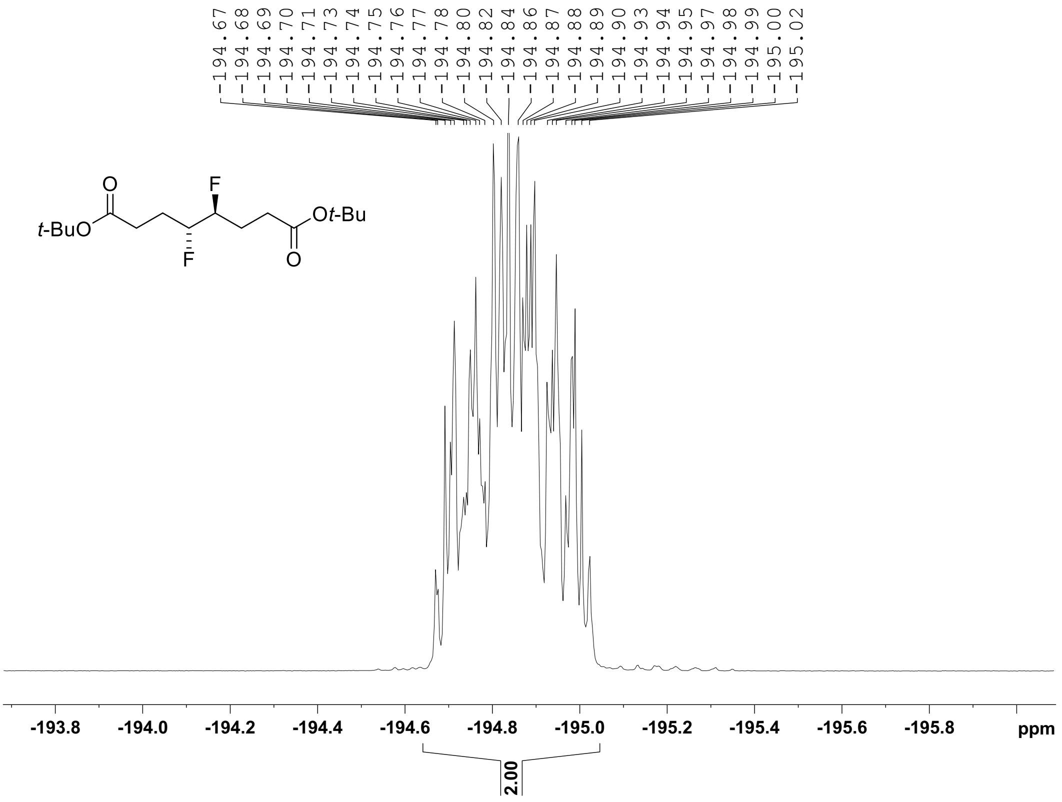

^{19}F{^{1}H} NMR (376 MHz, CDCl$_3$) of **17**

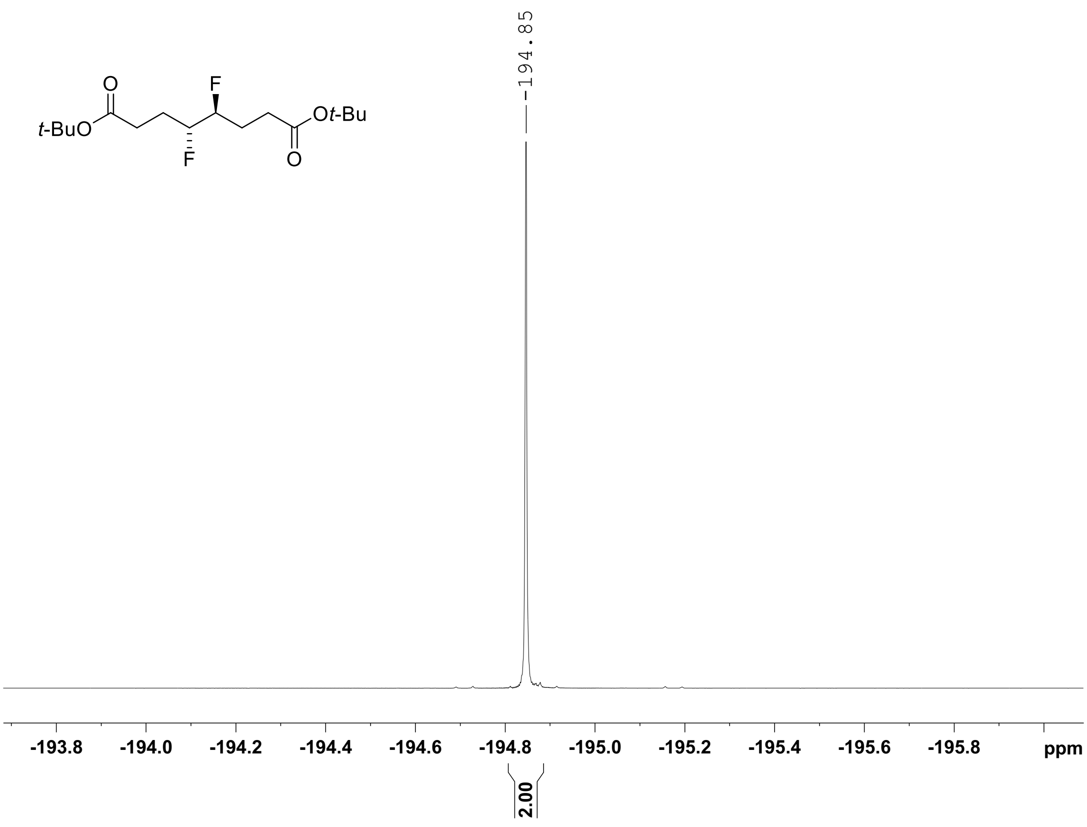

^{1}H NMR (600 MHz, MeOD) of **18**

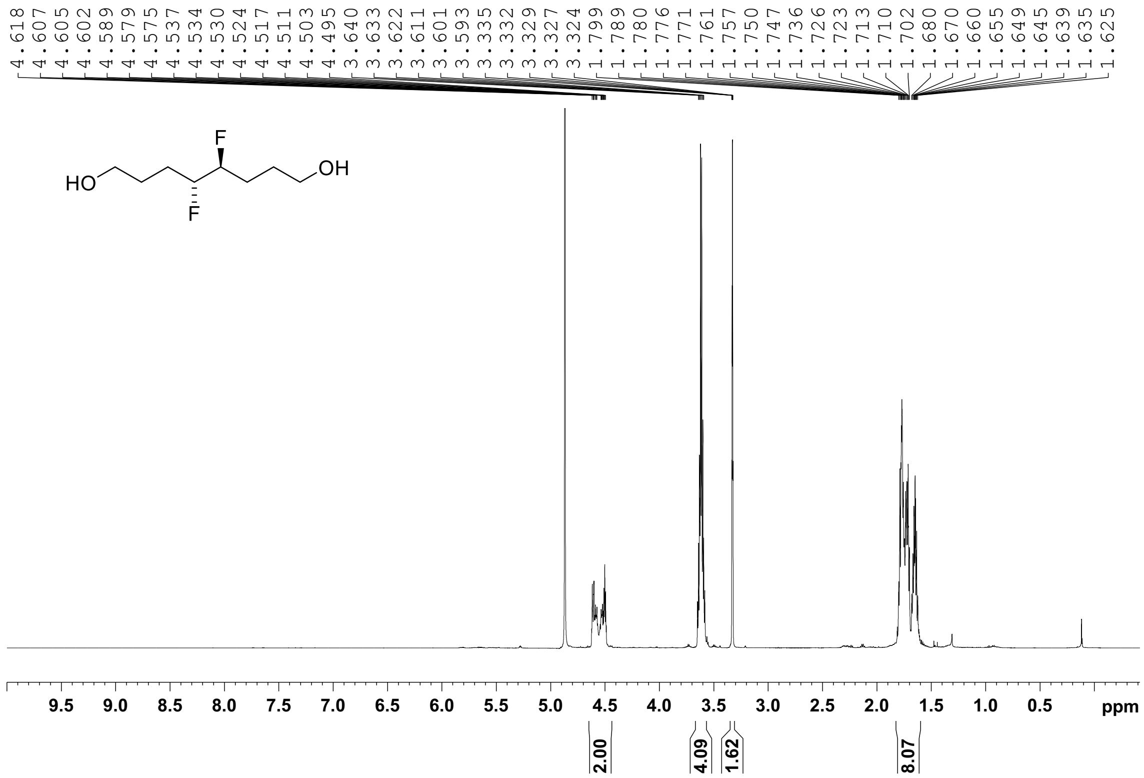

^{13}C{^{1}H} NMR (150 MHz, MeOD) of **18**

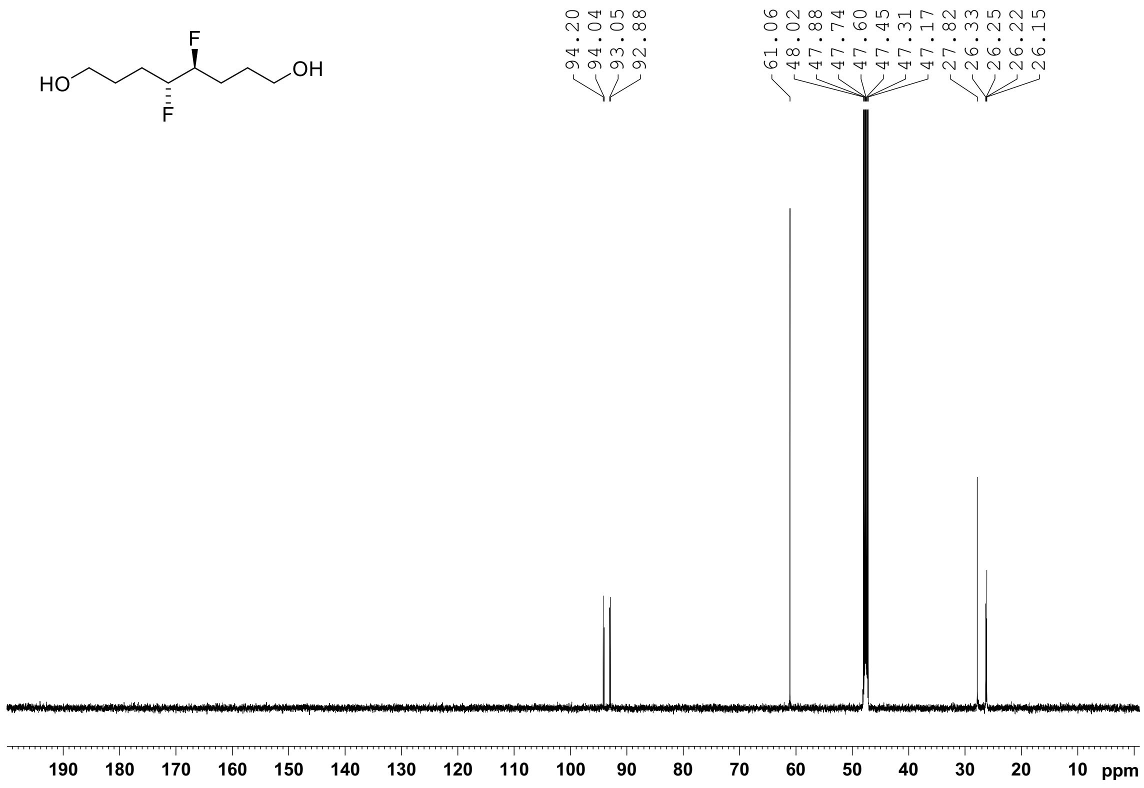

^{19}F NMR (376 MHz, MeOD) of **18**

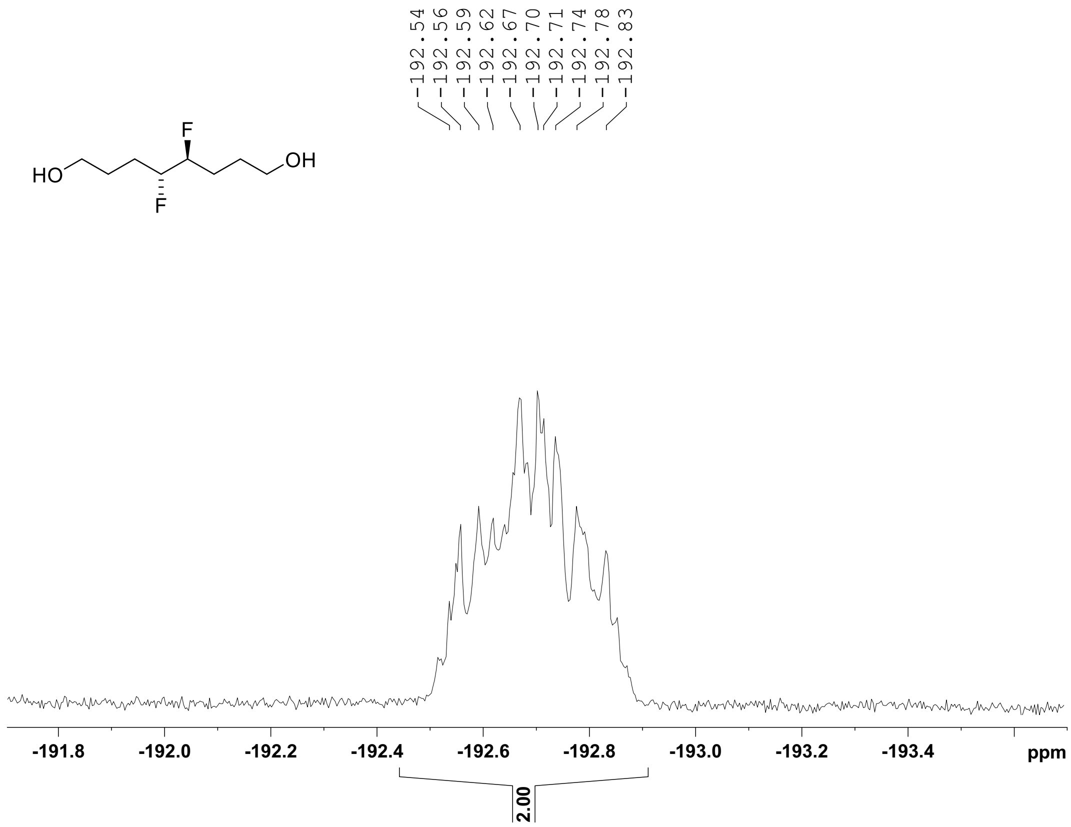

$^{19}F\{^1H\}$ NMR (376 MHz, MeOD) of **18**

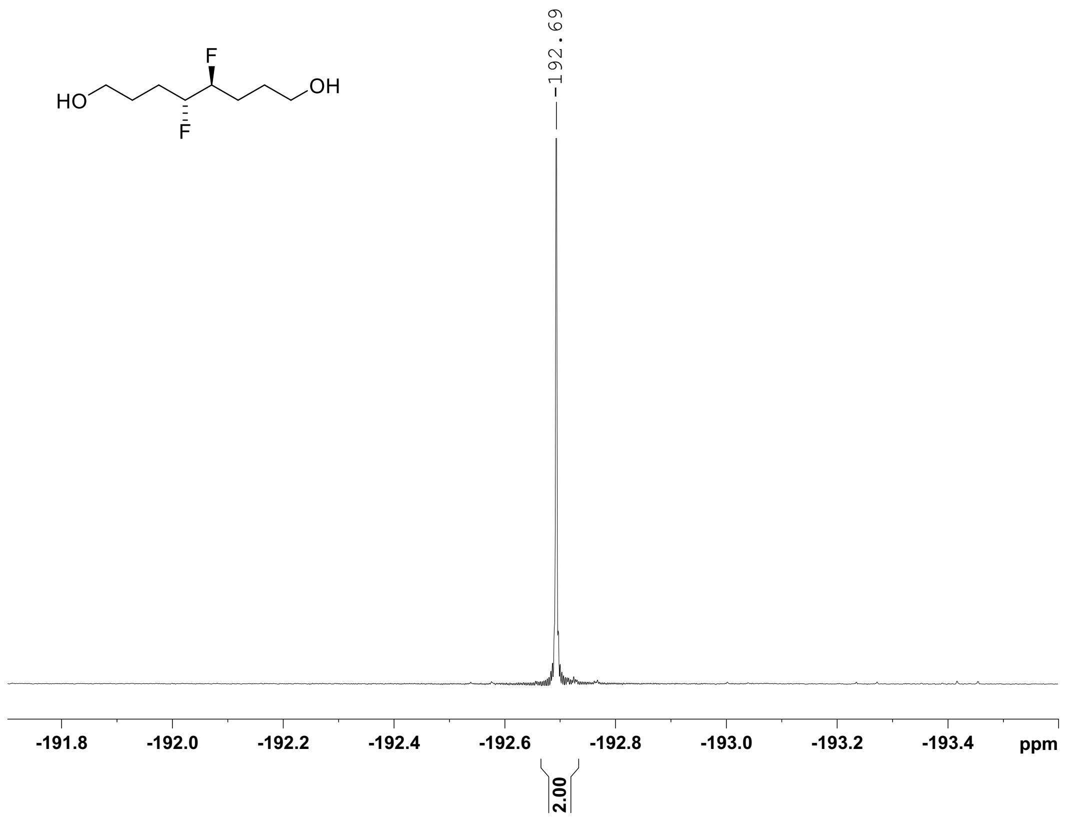

^{1}H NMR (400 MHz, $CDCl_3$) of **20**

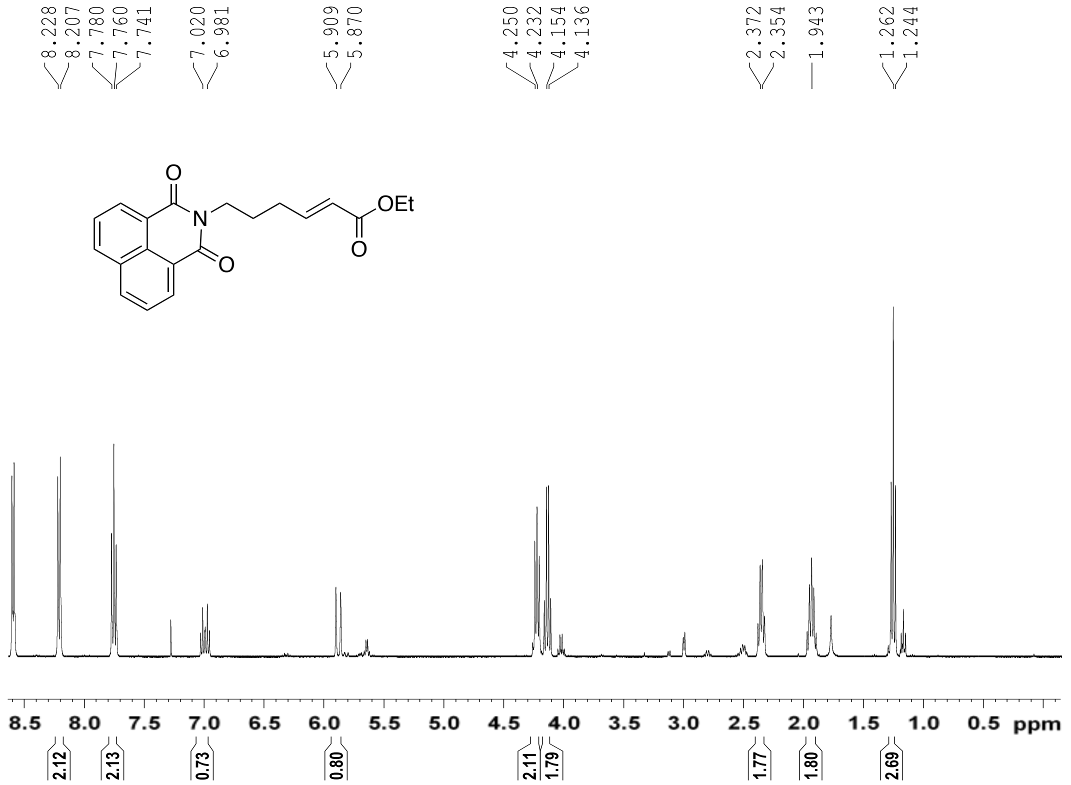

$^{13}C\{^{1}H\}$ NMR (100 MHz, $CDCl_3$) of **20**

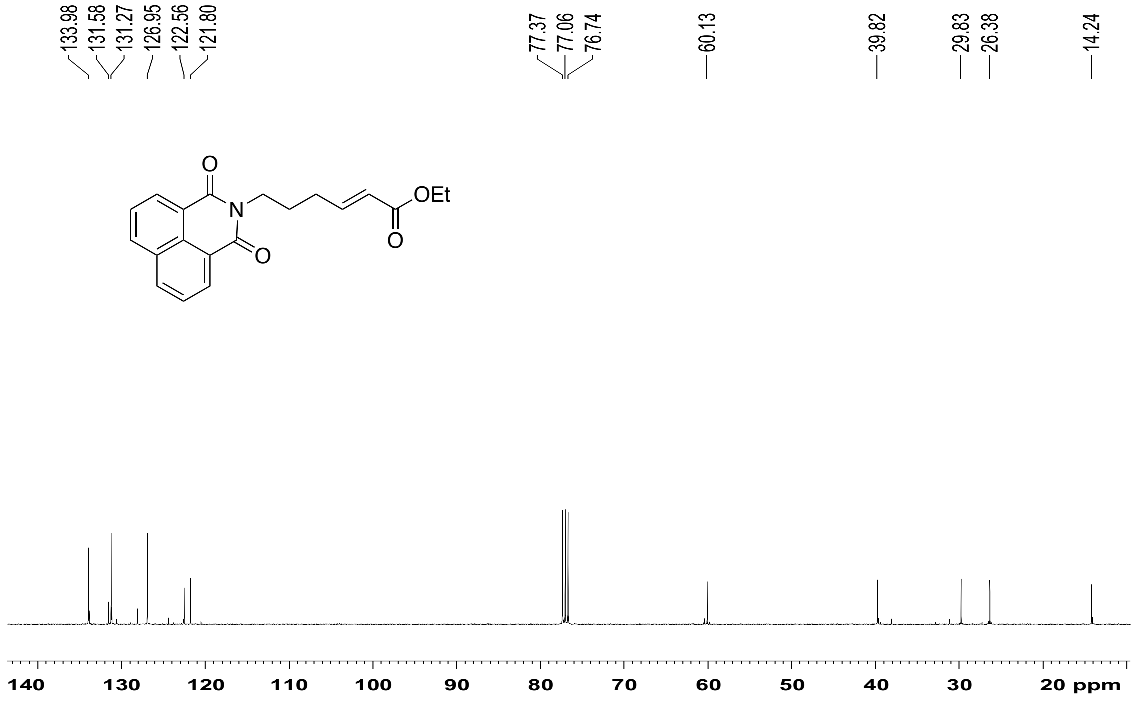

^{1}H NMR (400 MHz, $CDCl_3$) of **21**

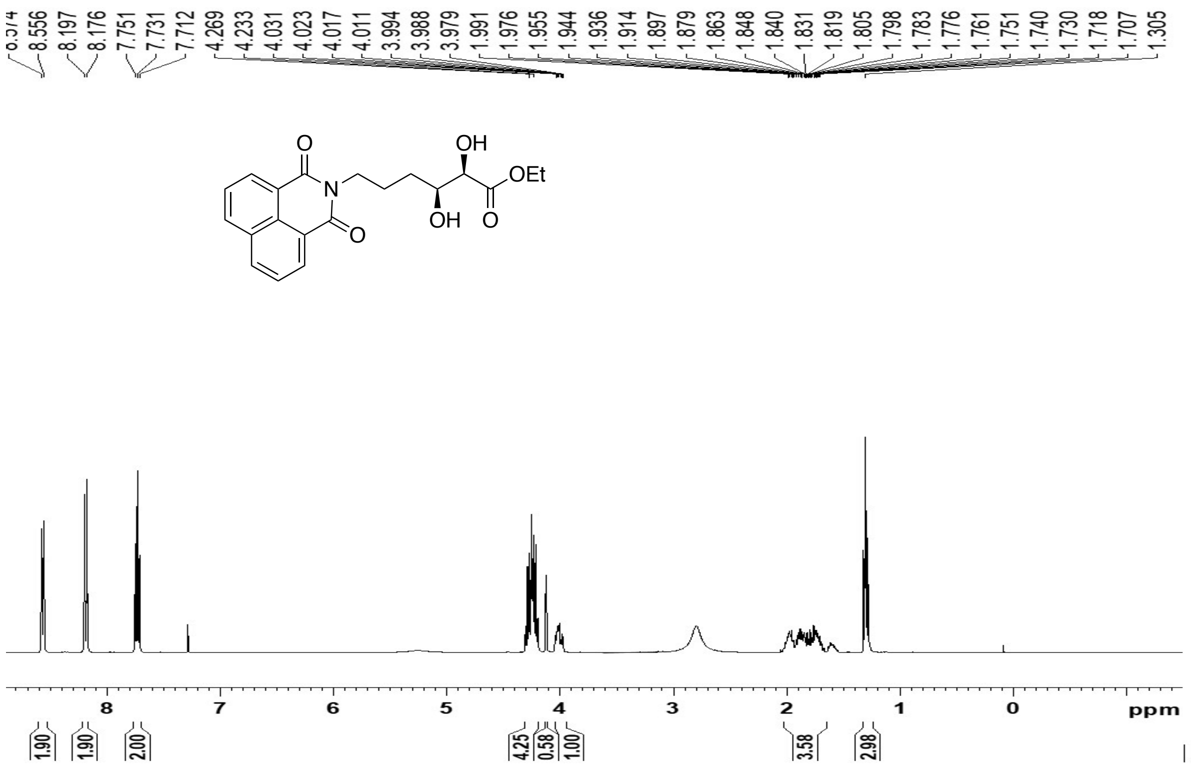

$^{13}C\{^{1}H\}$ NMR (100 MHz, $CDCl_3$) of **21**

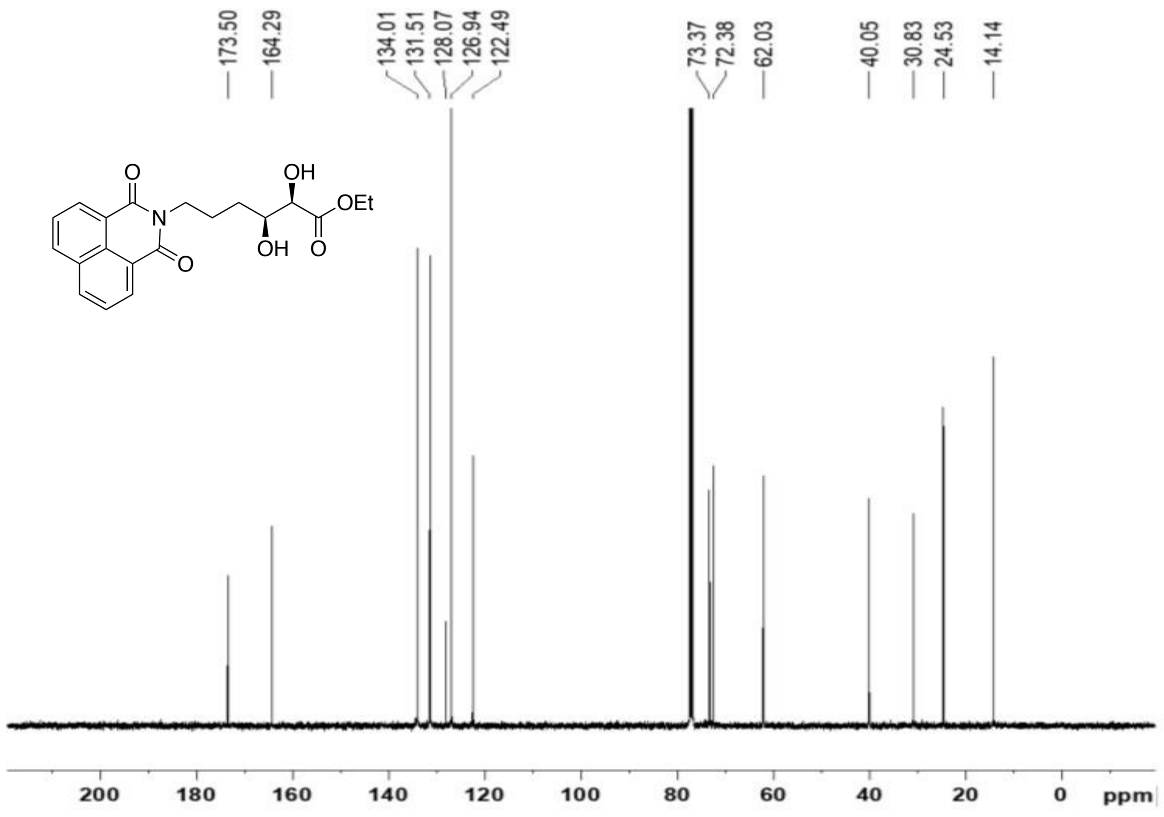

^{1}H NMR (400 MHz, $CDCl_3$) of **22**

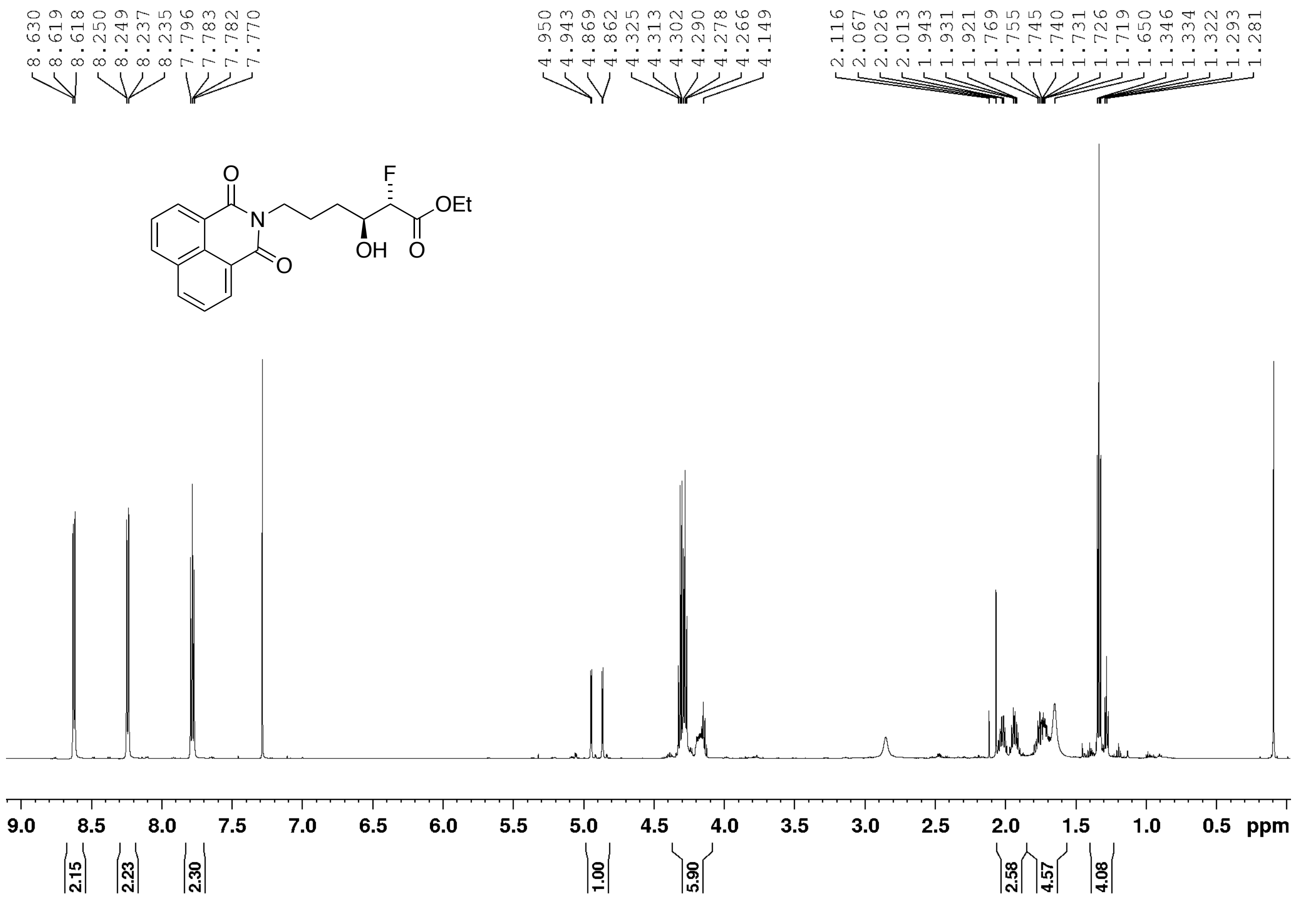

^{19}F NMR (282 MHz, $CDCl_3$) of **22**

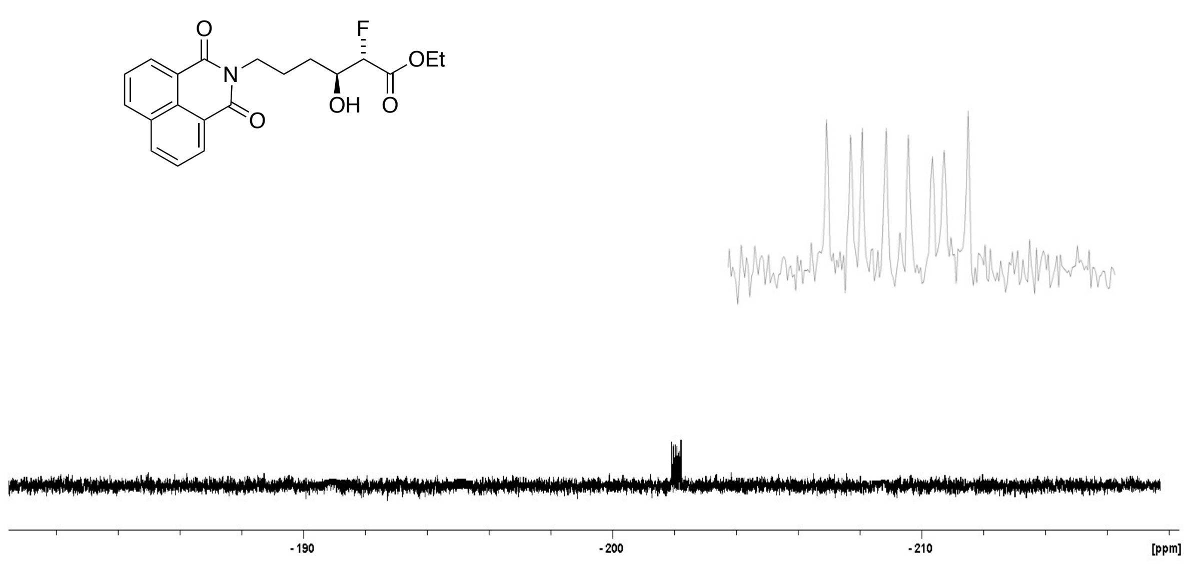

^{1}H NMR (600 MHz, $CDCl_3$) of **23**

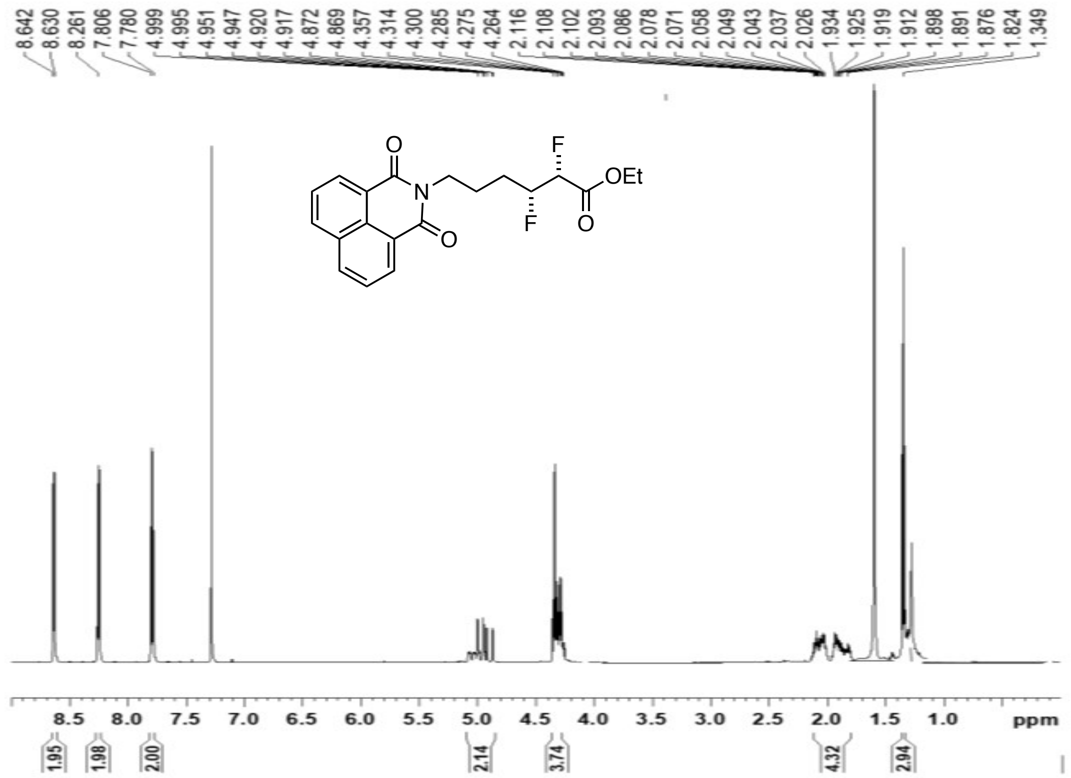

$^{13}C\{^{1}H\}$ NMR (75 MHz, $CDCl_3$) of **23**

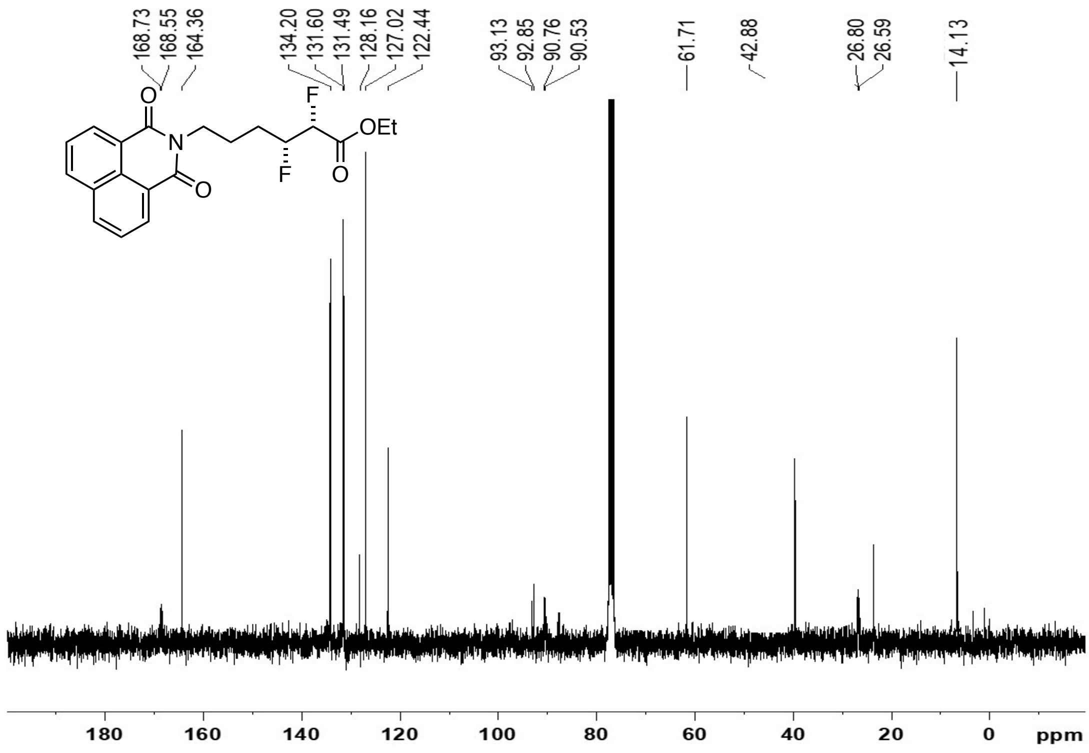

^{19}F NMR (282 MHz, CDCl$_3$) of **23**

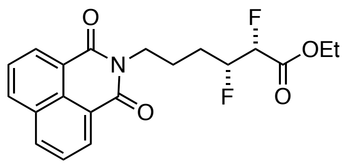

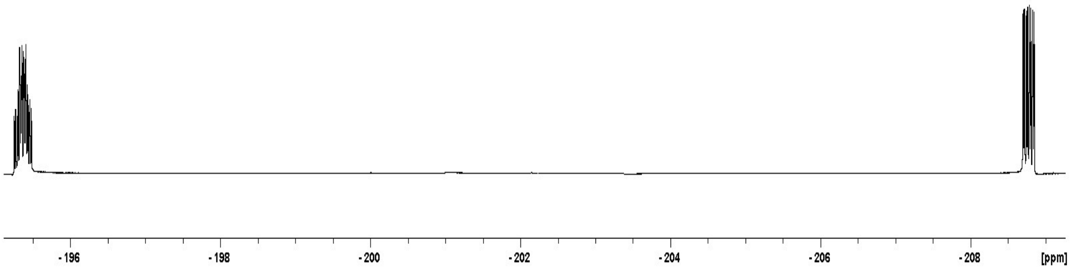

^{1}H NMR (600 MHz, CDCl$_3$) of **24**

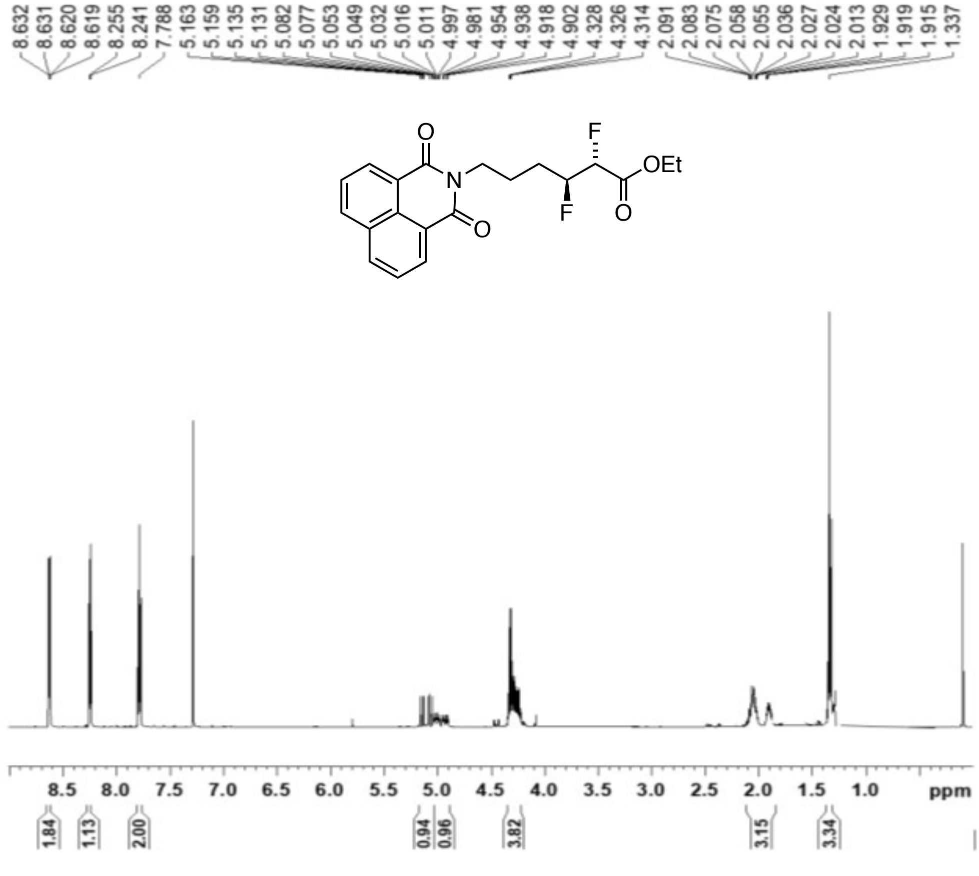

$^{13}C\{^{1}H\}$ NMR (75 MHz, $CDCl_3$) of **24**

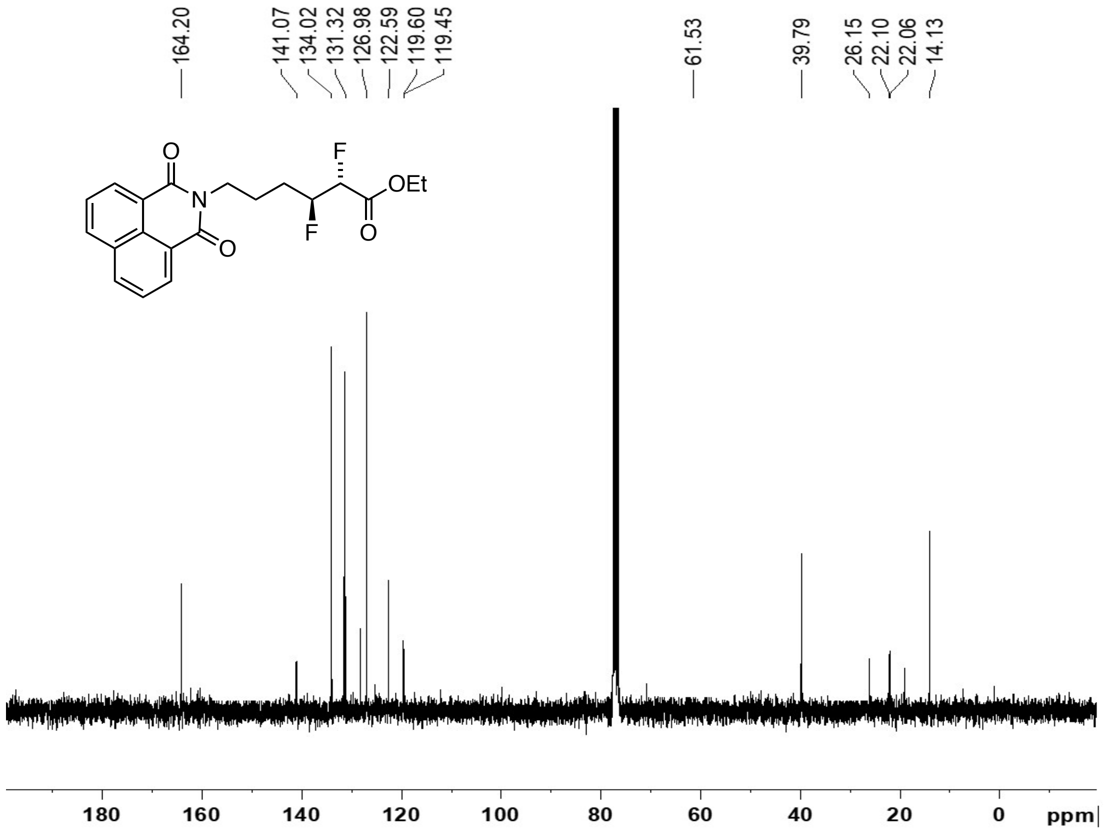

^{19}F NMR (282 MHz, $CDCl_3$) of **24**

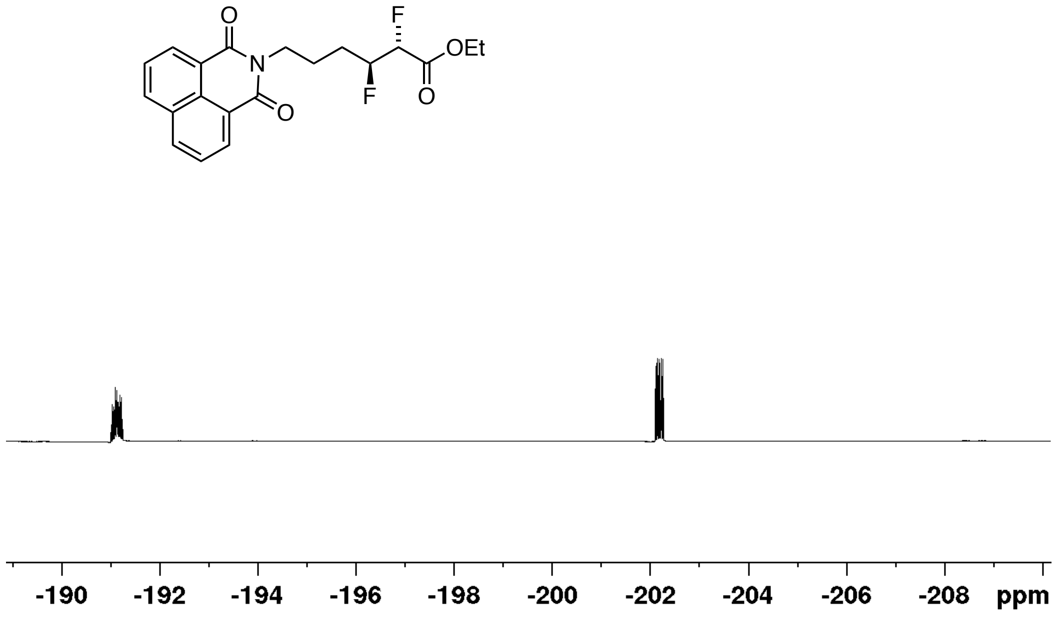

^{1}H NMR (400 MHz, MeOD) of **25**

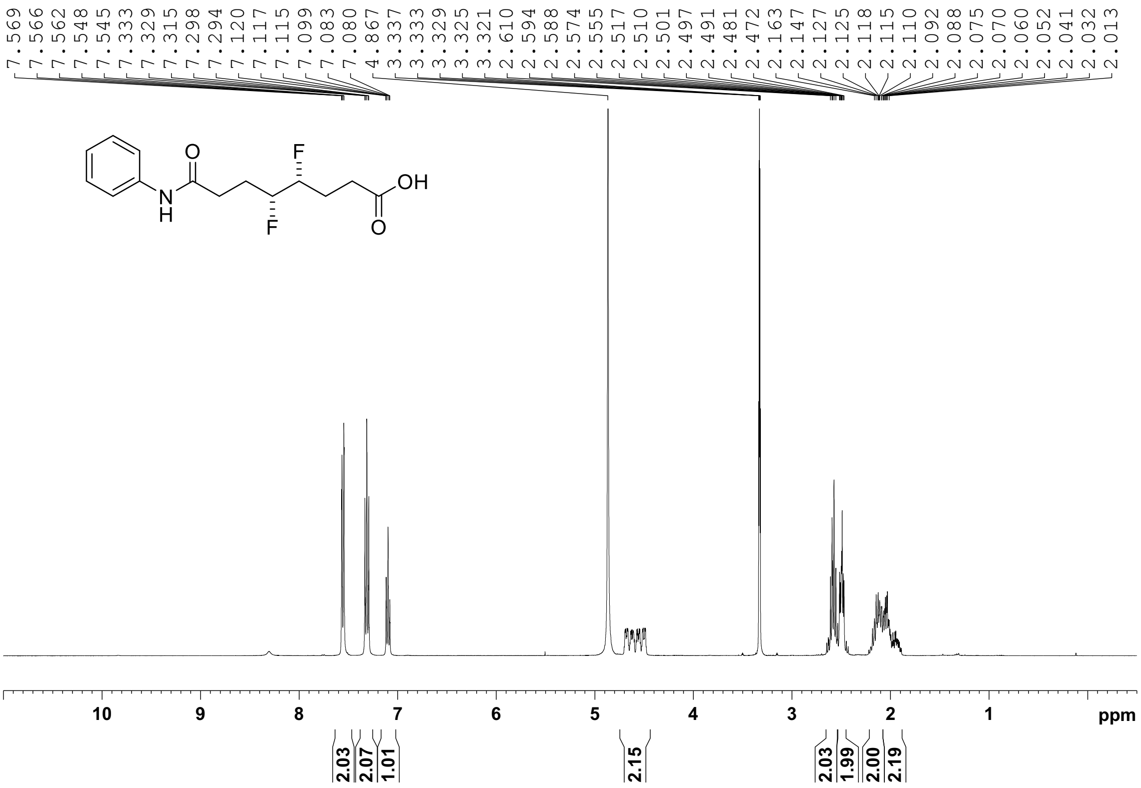

$^{13}C\{^{1}H\}$ NMR (100 MHz, MeOD) of **25**

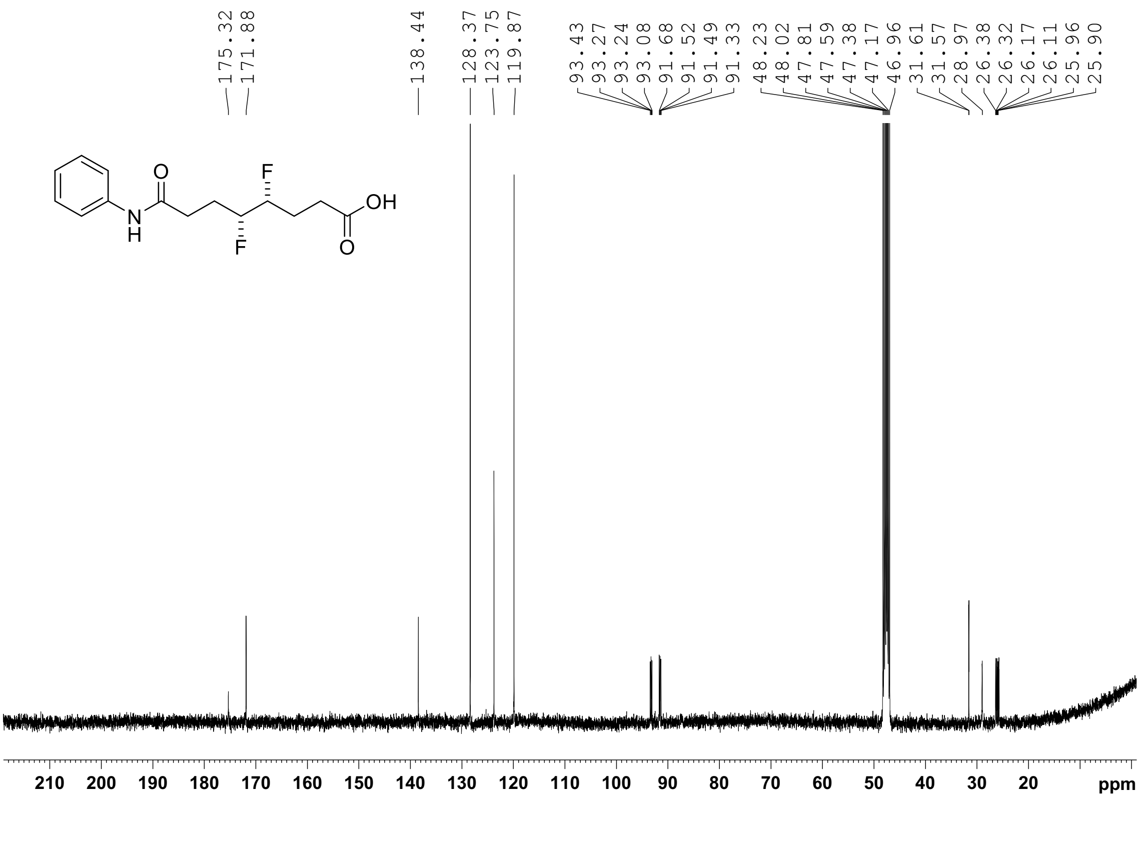

^{19}F NMR (376 MHz, MeOD) of **25**

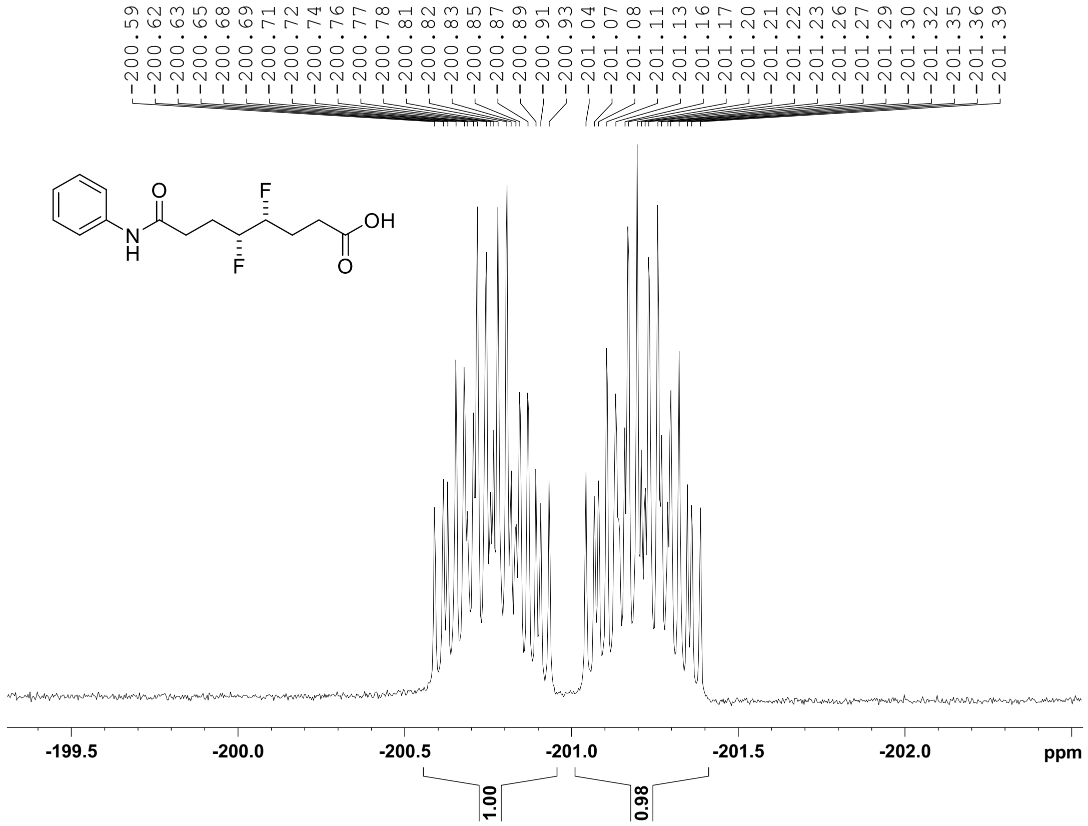

^{19}F{^{1}H} NMR (376 MHz, MeOD) of **25**

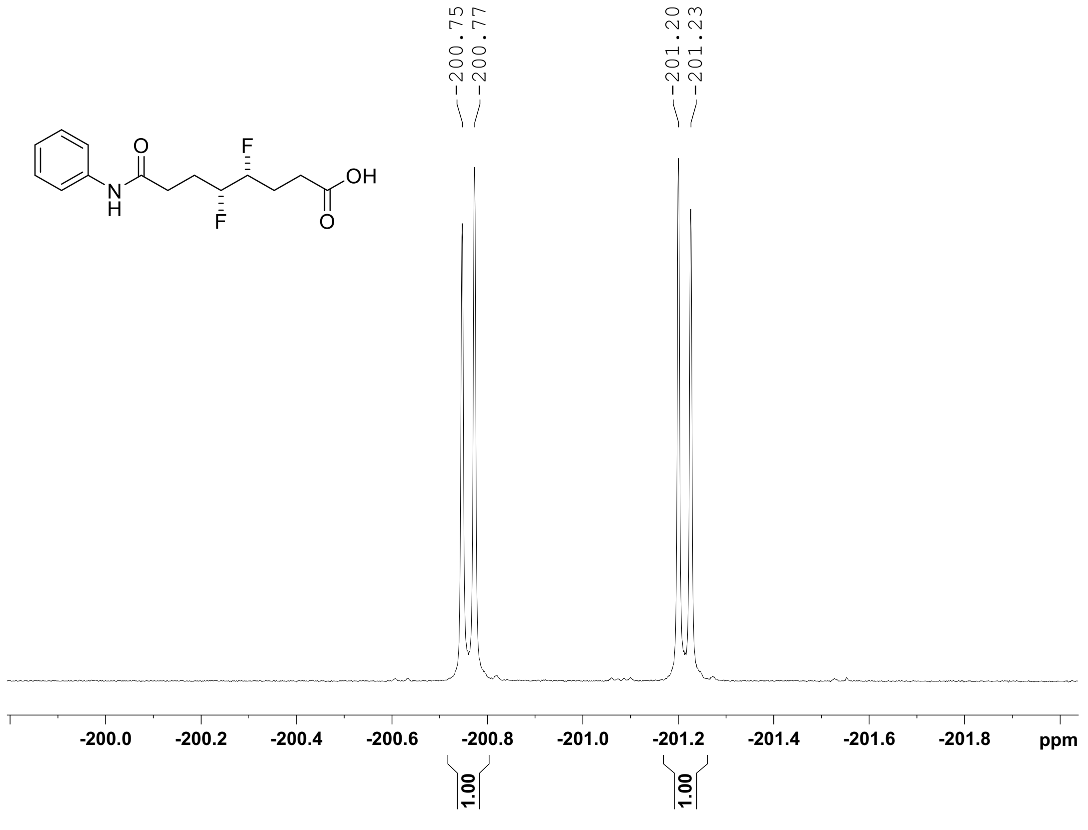

^{1}H NMR (400 MHz, $CDCl_3$) of **26**

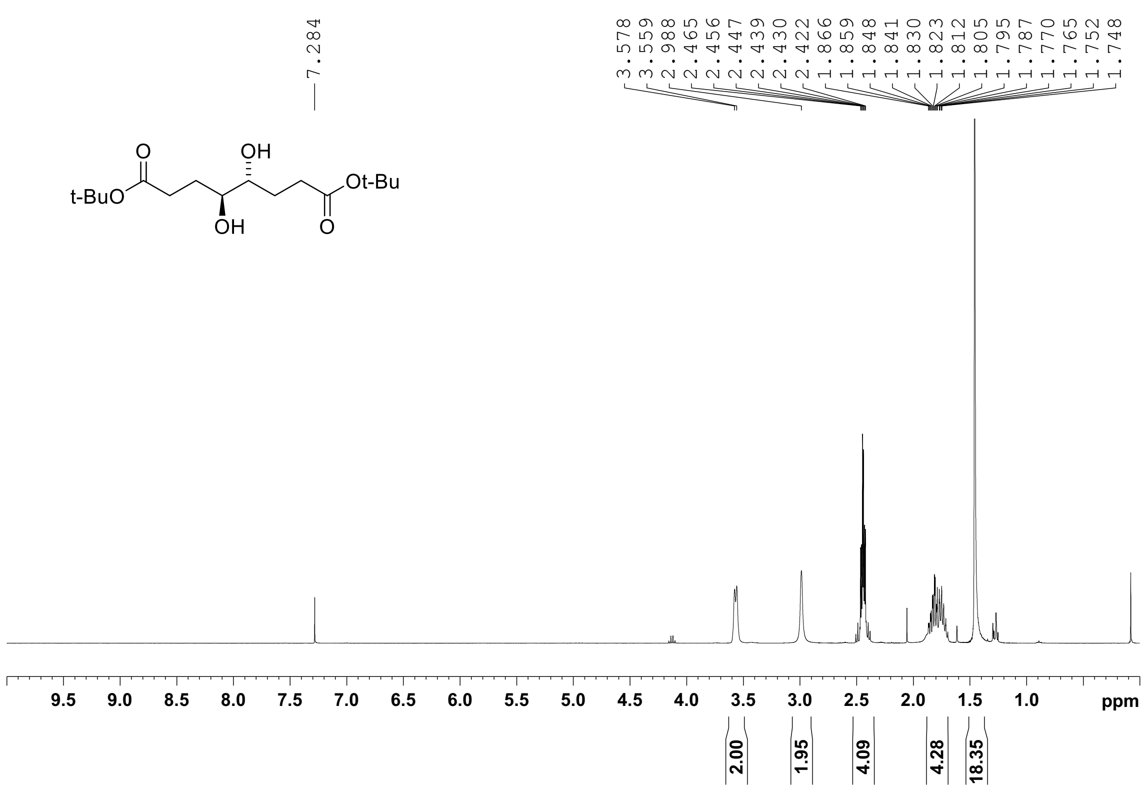

$^{13}C\{^{1}H\}$ NMR (100 MHz, $CDCl_3$) of **26**

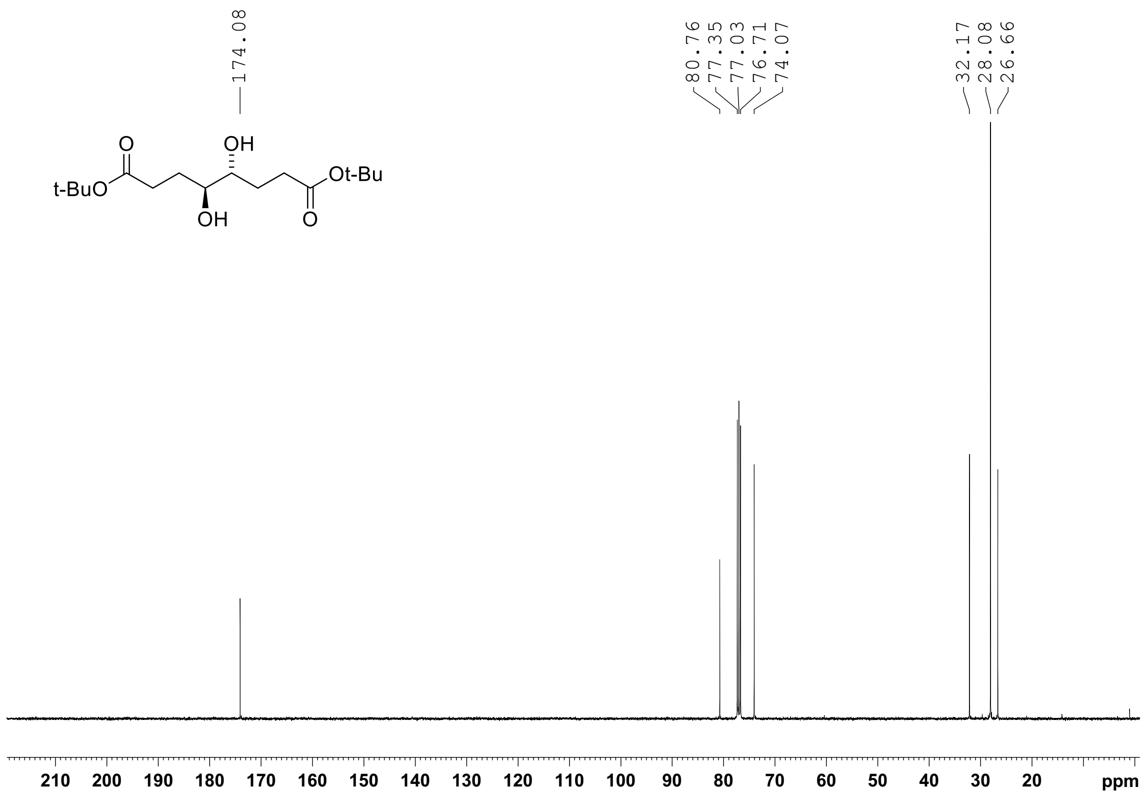

^{1}H NMR (600 MHz, $CDCl_3$) of **27**

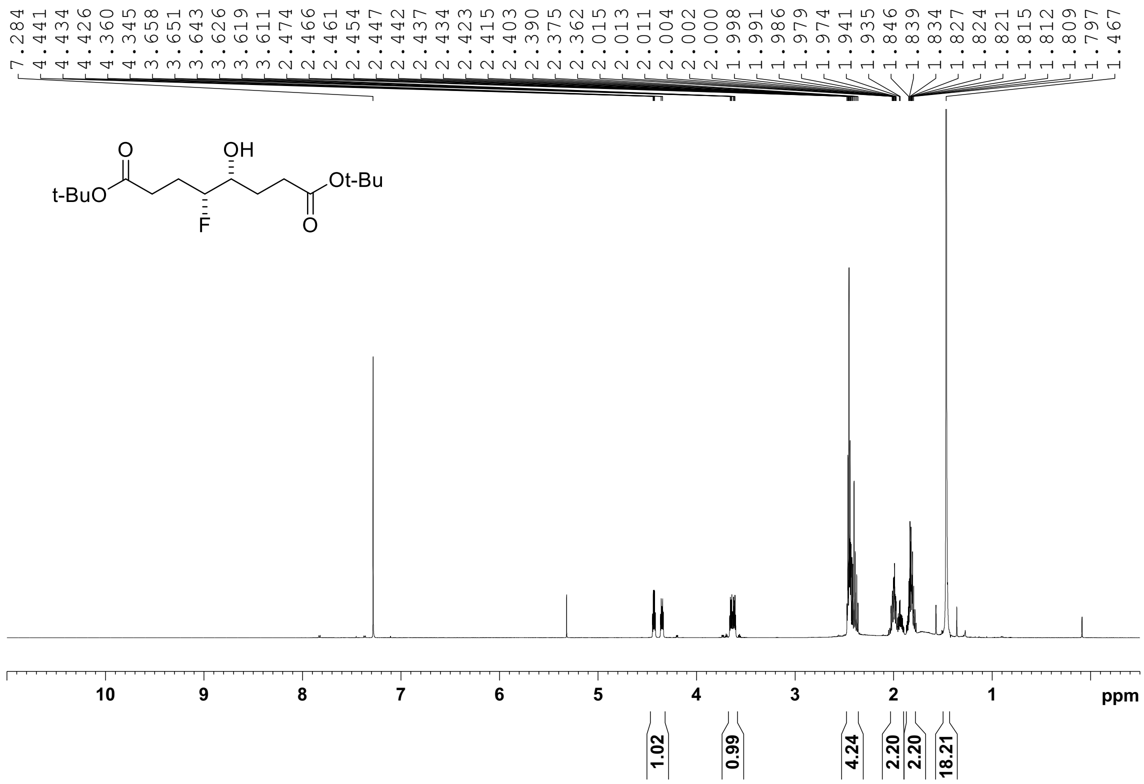

$^{13}C\{^1H\}$ NMR (150 MHz, $CDCl_3$) of **27**

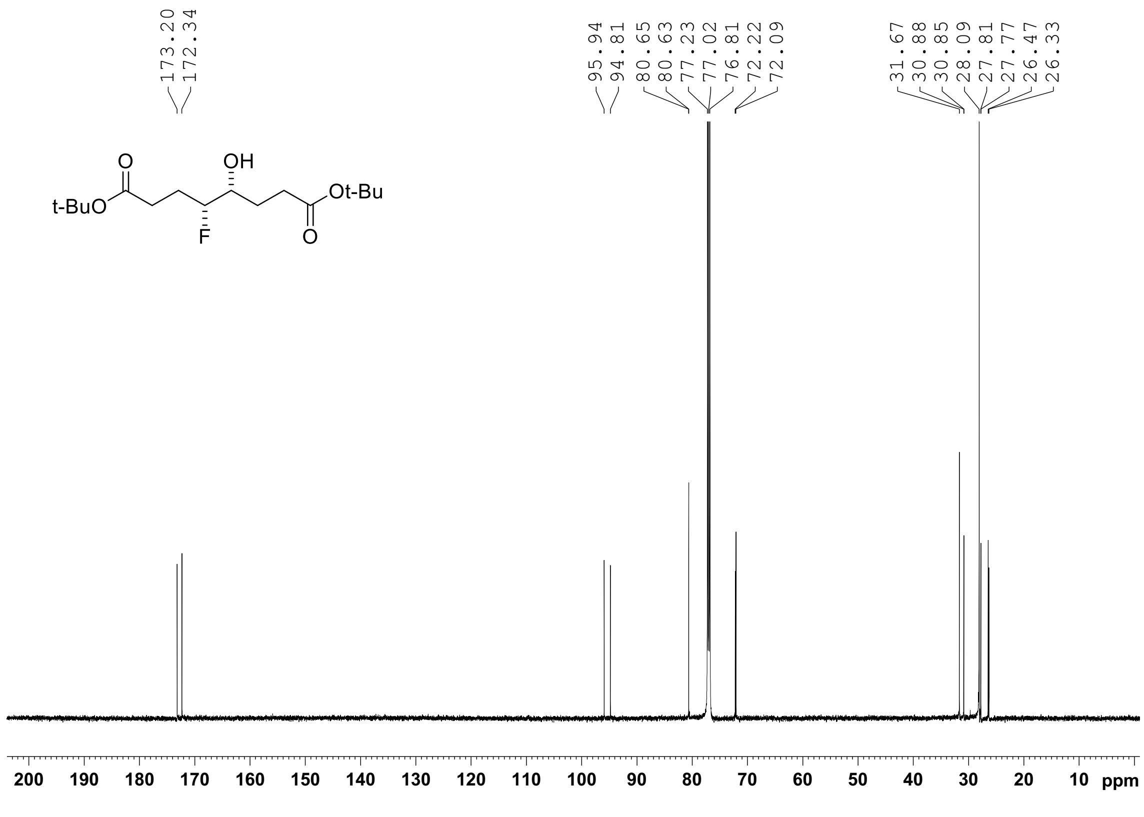

^{19}F NMR (376 MHz, $CDCl_3$) of **27**

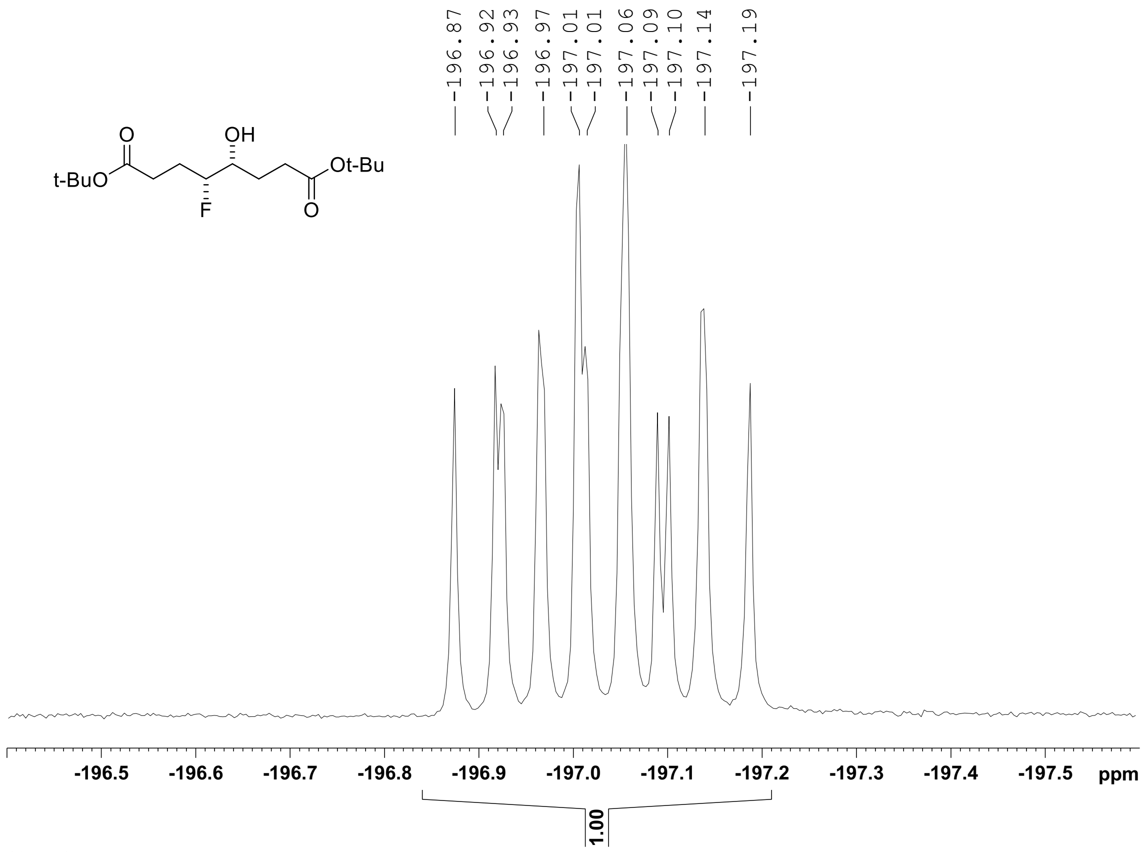

$^{19}F\{^1H\}$ NMR (376 MHz, $CDCl_3$) of **27**

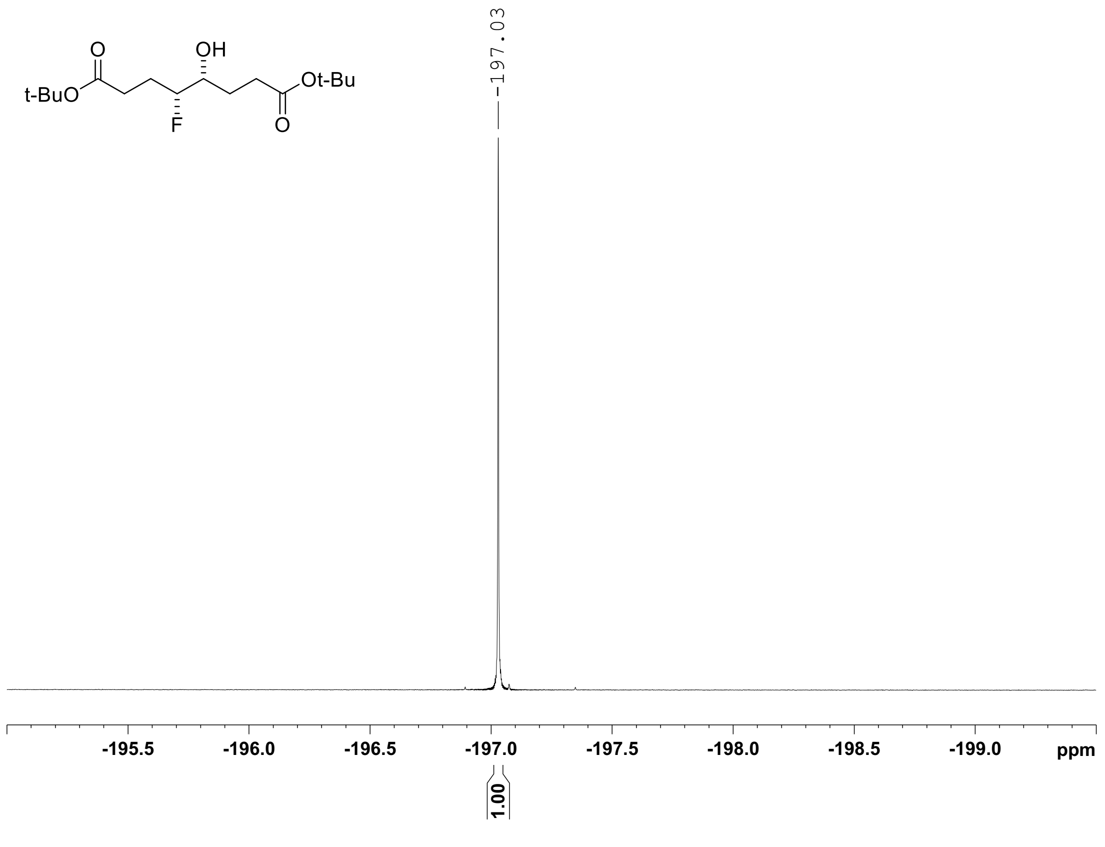

^{1}H NMR (400 MHz, MeOD) of **28**

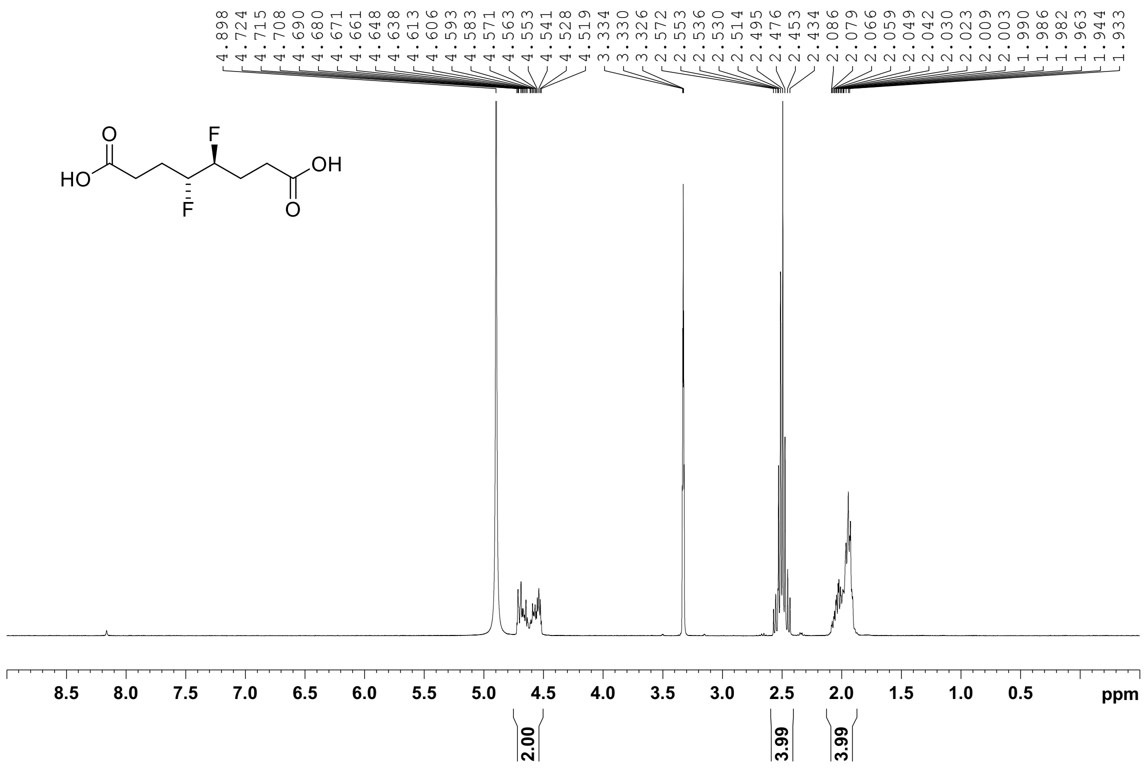

$^{13}C\{^{1}H\}$ NMR (100 MHz, MeOD) of **28**

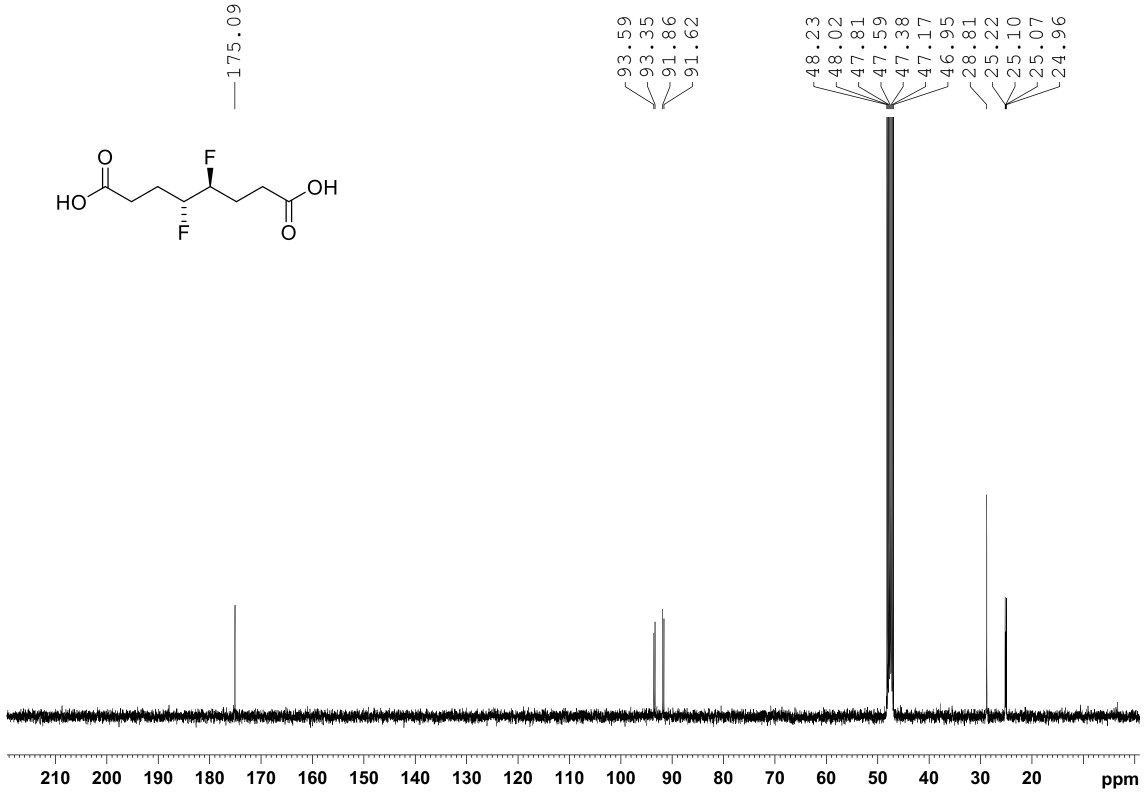

^{19}F NMR (376 MHz, MeOD) of **28**

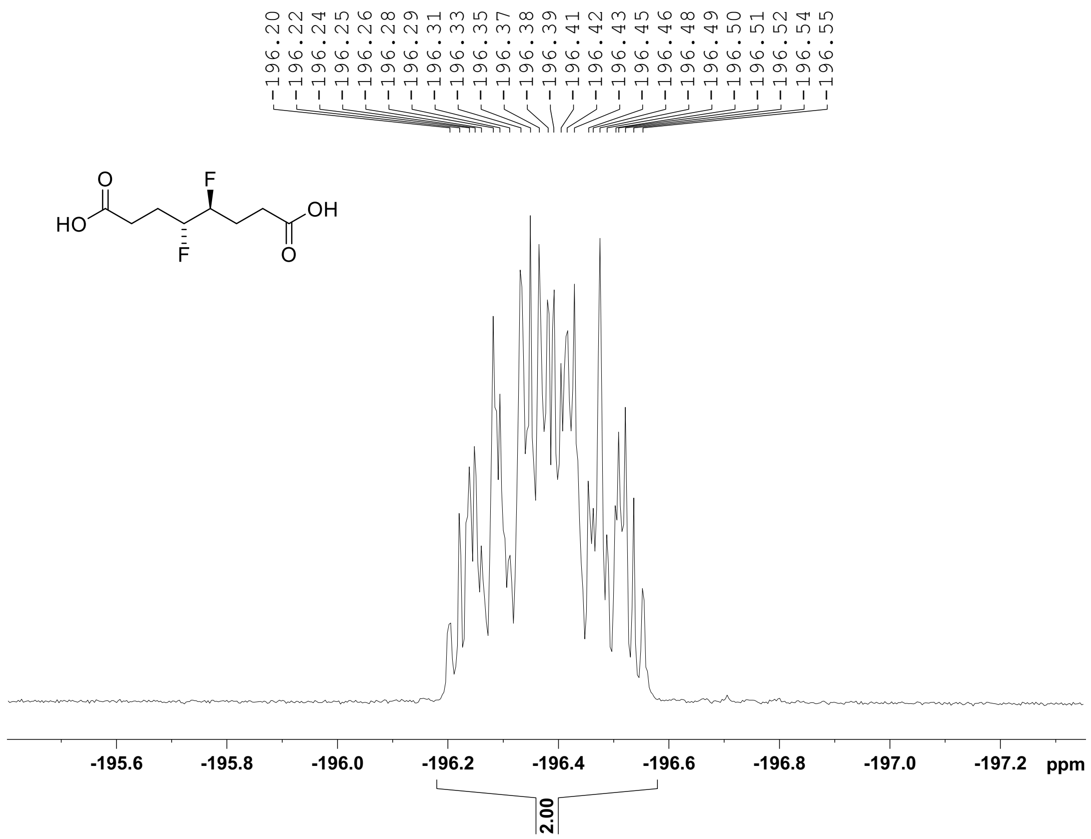

^{19}F{^{1}H} NMR (376 MHz, MeOD) of **28**

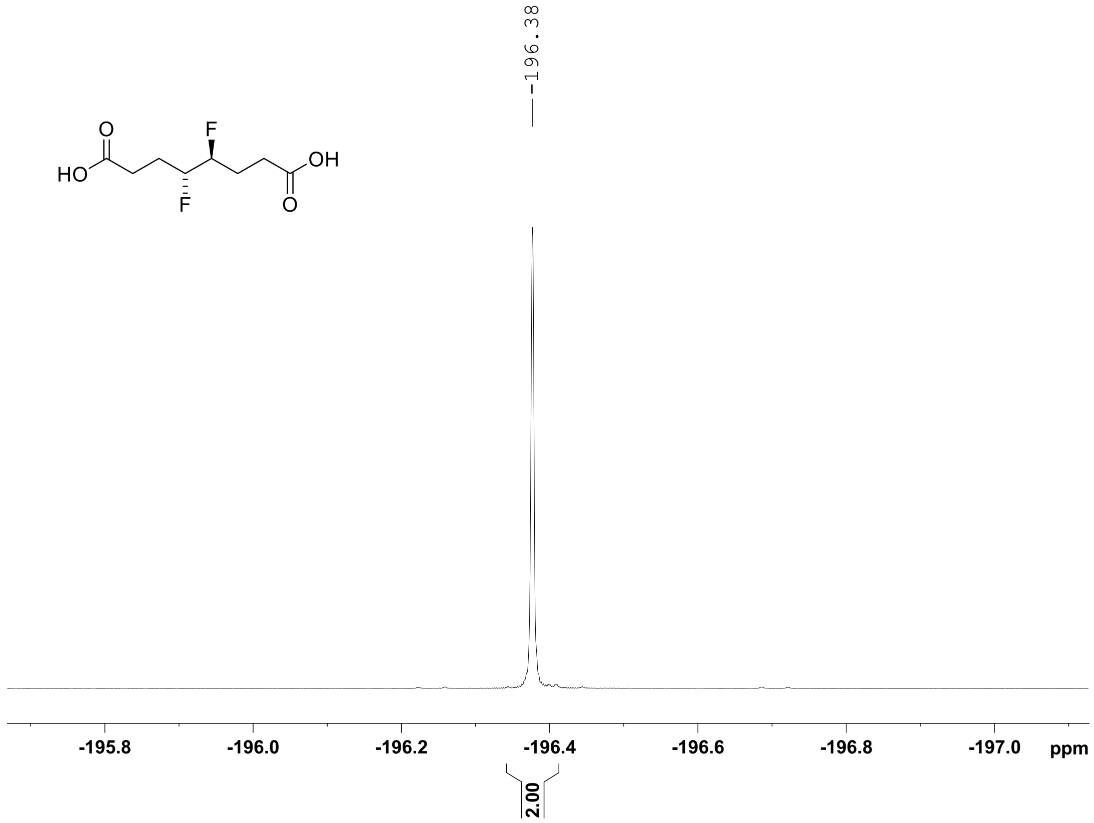

^{1}H NMR (400 MHz, MeOD) of **29**

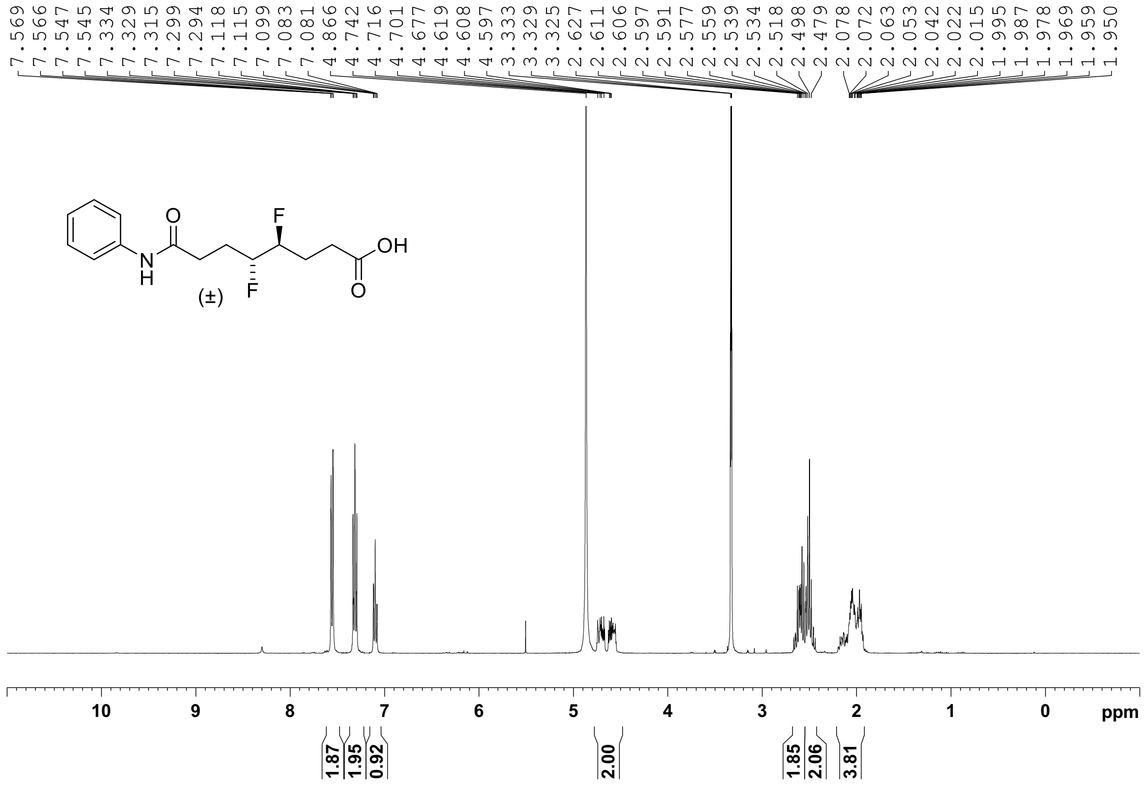

$^{13}C\{^{1}H\}$ NMR (100 MHz, MeOD) of **29**

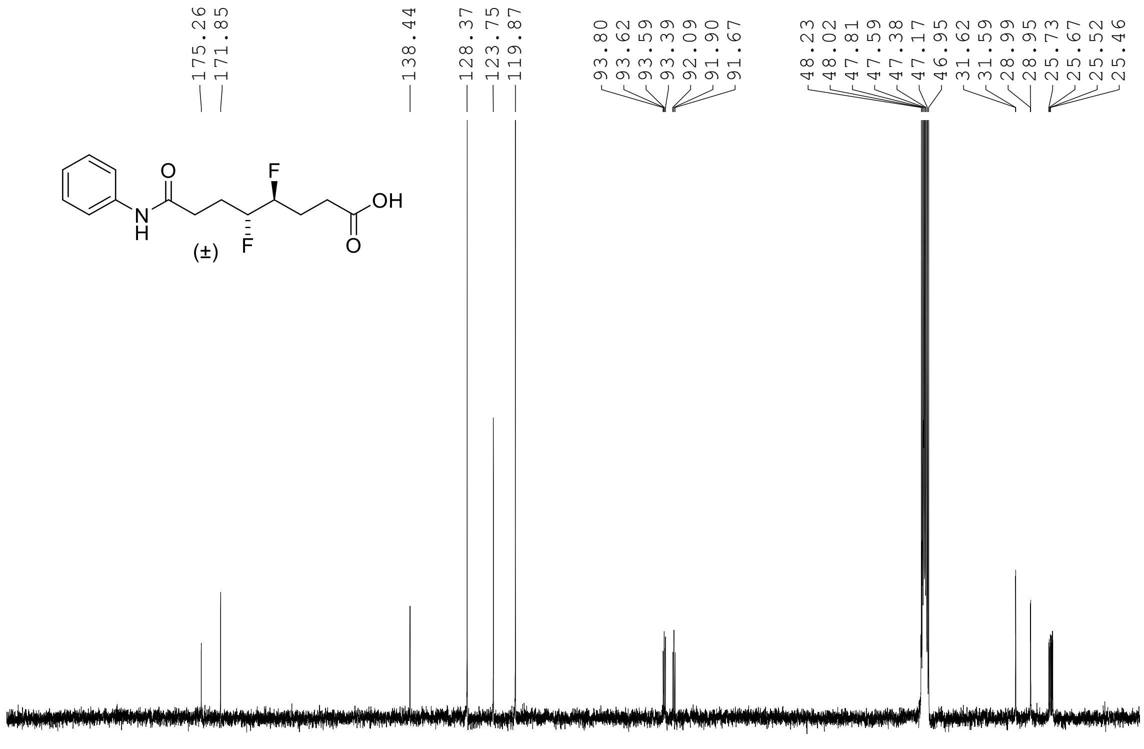

^{19}F NMR (376 MHz, MeOD) of **29**

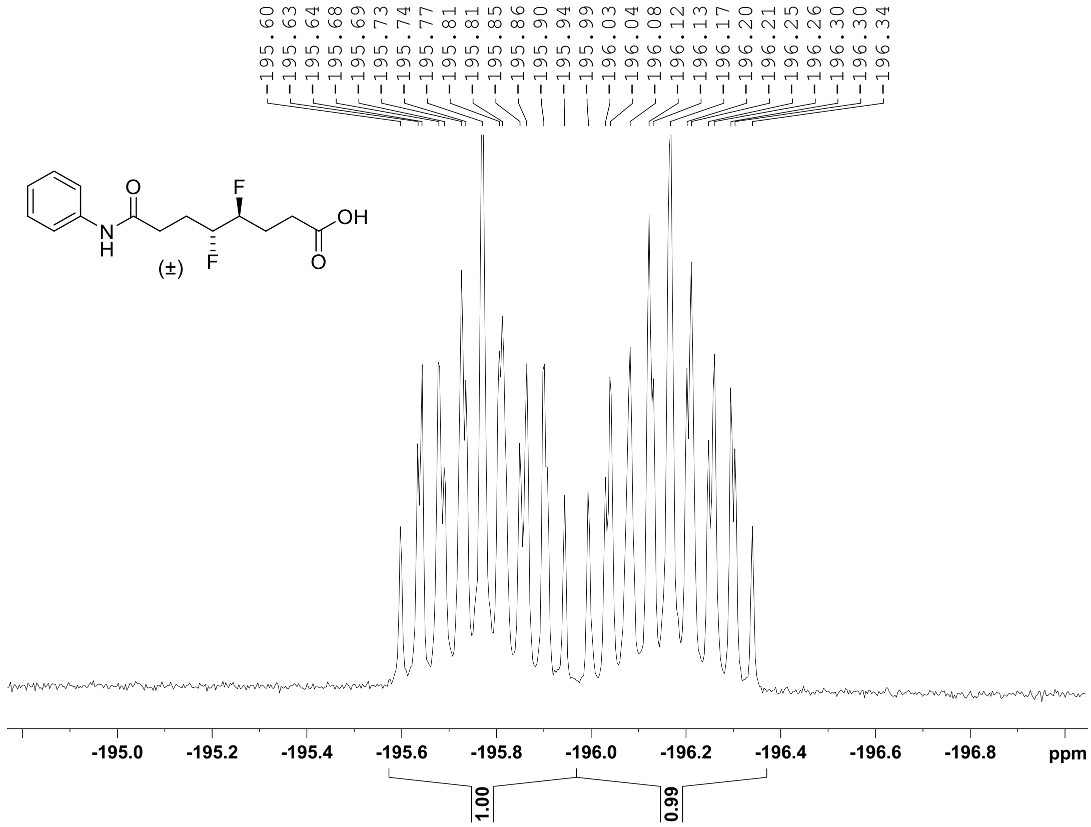

^{19}F{^{1}H} NMR (376 MHz, MeOD) of **29**

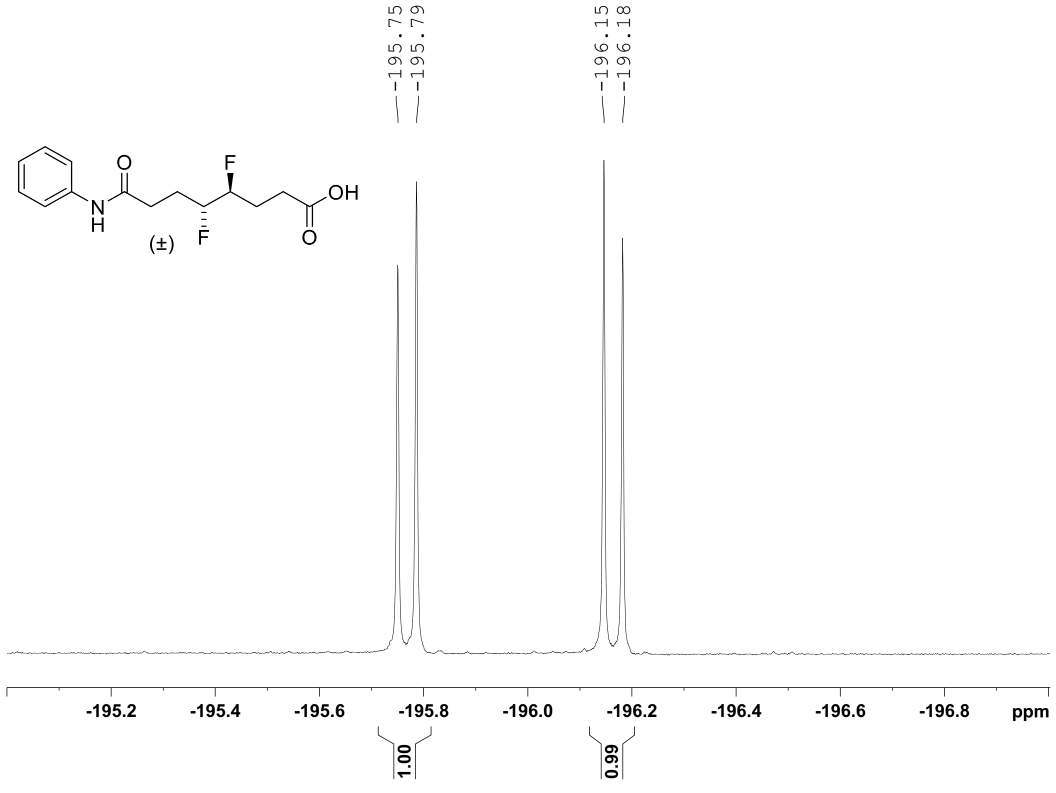

^{1}H NMR (400 MHz, MeOD) of **31**

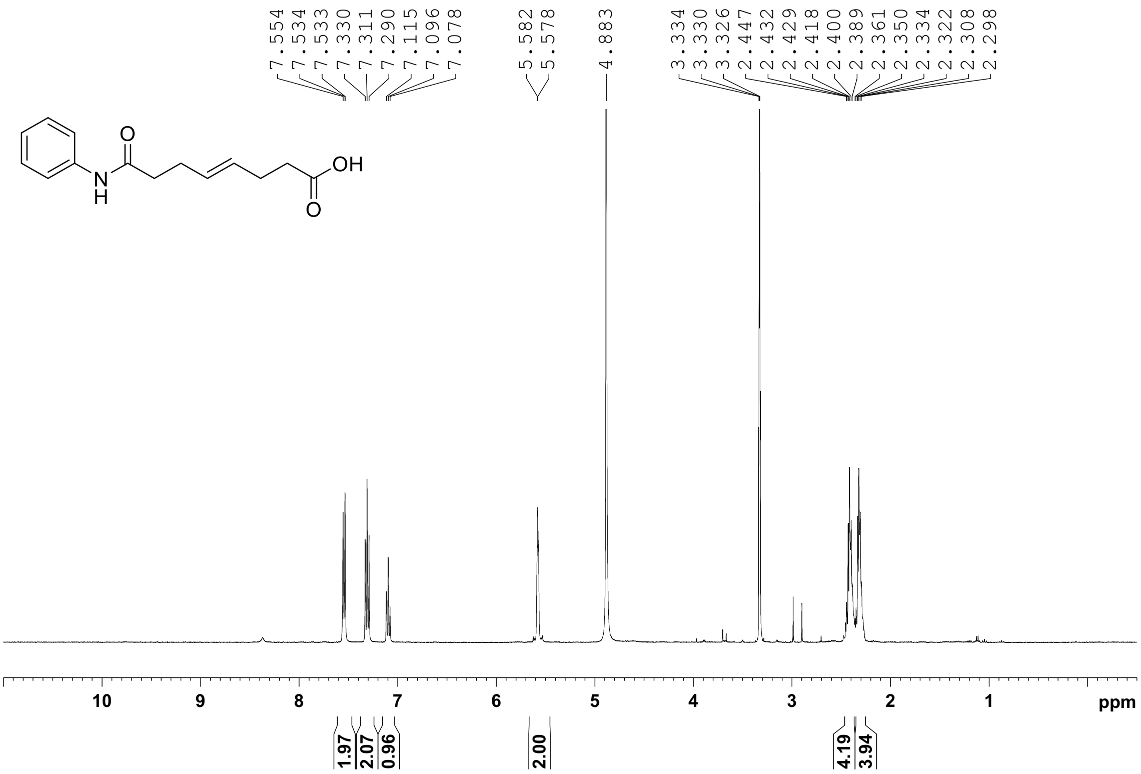

$^{13}C\{^{1}H\}$ NMR (100 MHz, MeOD) of **31**

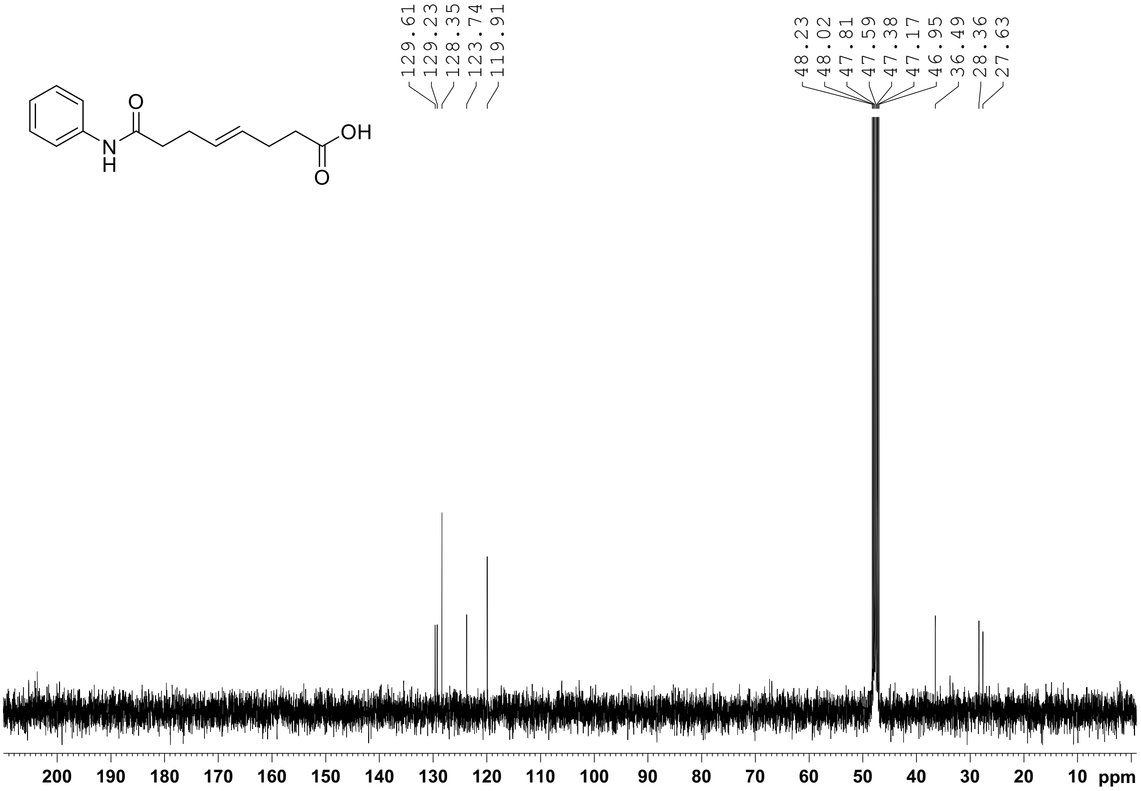

^{1}H NMR (400 MHz, MeOD) of **33**

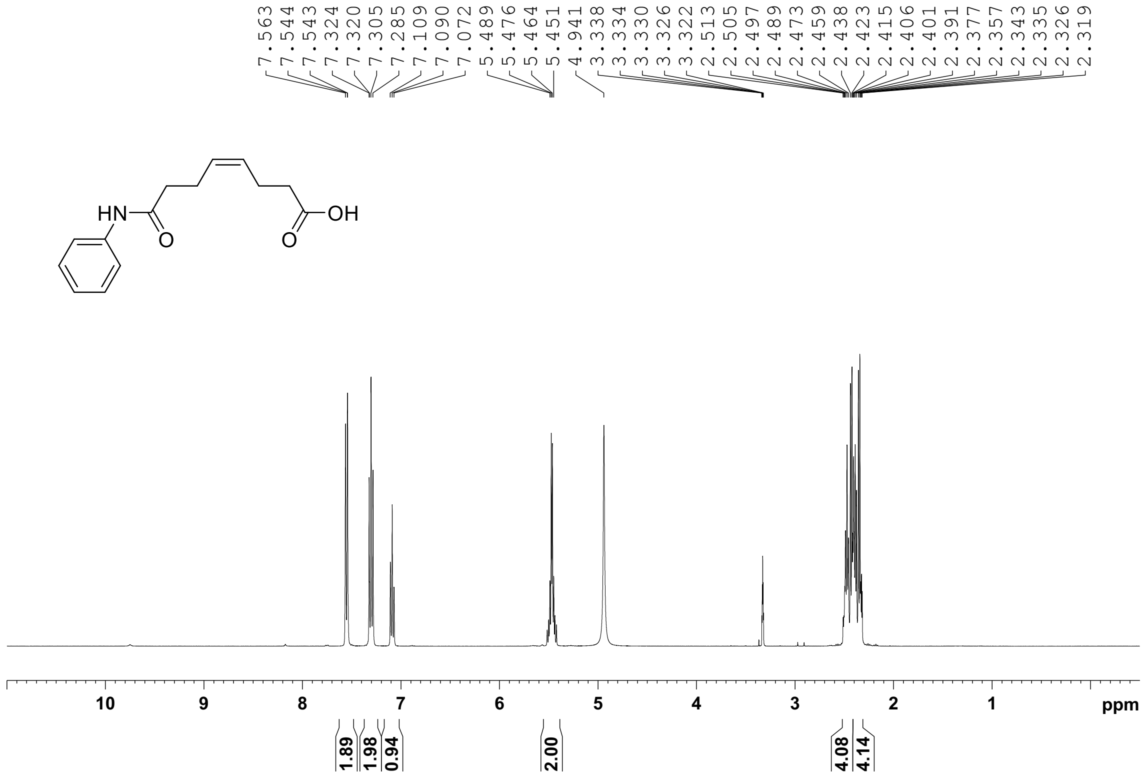

^{13}C{^{1}H} NMR (100 MHz, MeOD) of **33**

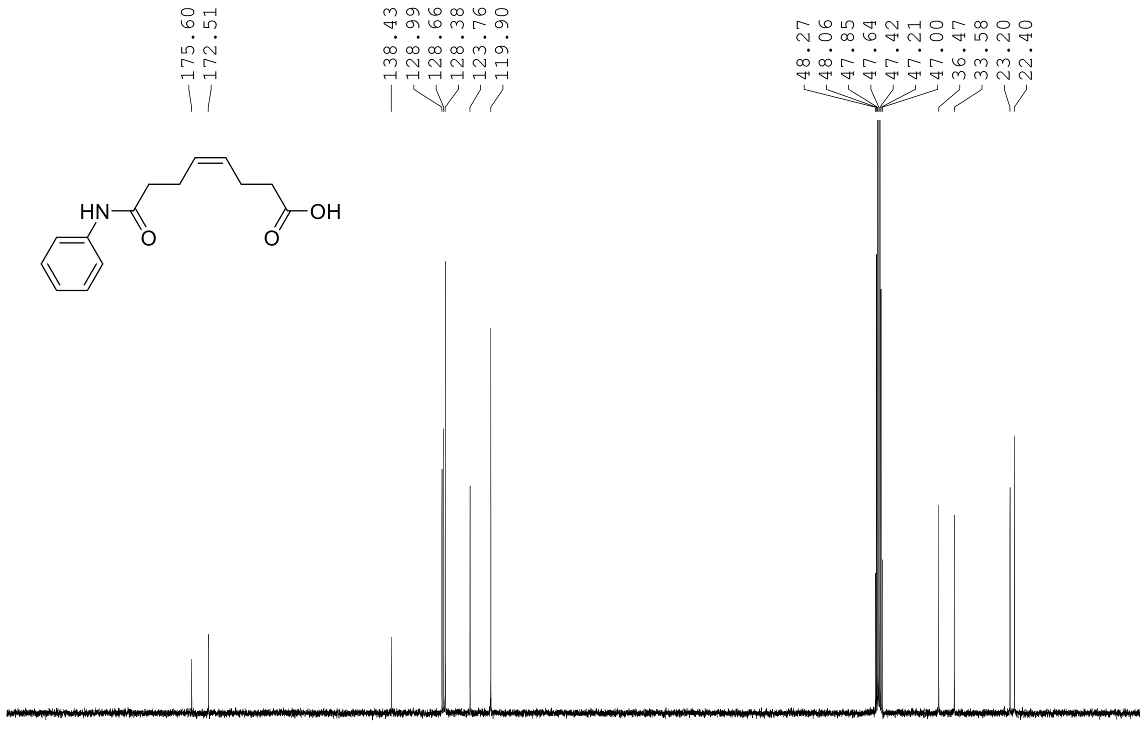

5. Solid-state structures of 14 and 18

X-ray crystal structures were obtained for each of the difluorinated diols **14** and **18** (Figure 1). In the solid state, both compounds adopt a fully extended zigzag conformation. The *threo*-difluorinated diol **14**, which lacks a centre of symmetry, crystallises in the monoclinic $P2_1$ space group; the F–C–C–F dihedral angle is 66.5°. The *erythro*-difluorinated diol **18** crystallises in the monoclinic $P2_1/c$ space group with a centre of symmetry at the midpoint of the central C–C bond; the F–C–C–F dihedral angle is exactly 180°.

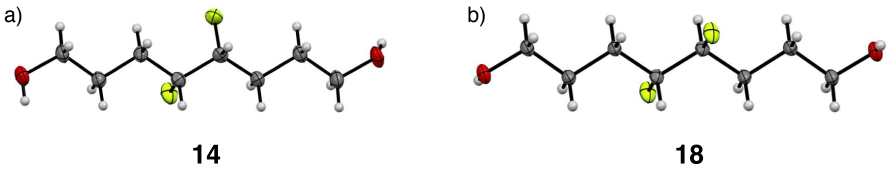

Figure 1. Crystal structures of a) *threo*-difluoroalkane **14** and b) *erythro*-difluoroalkane **18**.

In both crystal structures, the crystal packing arrangement is remarkably similar (Figures 2 and 3). The fluorinated and hydroxylated domains are quite separate in each case. The major intermolecular interactions in each case are C–F···H and OH···H dipolar interactions / hydrogen bonds.

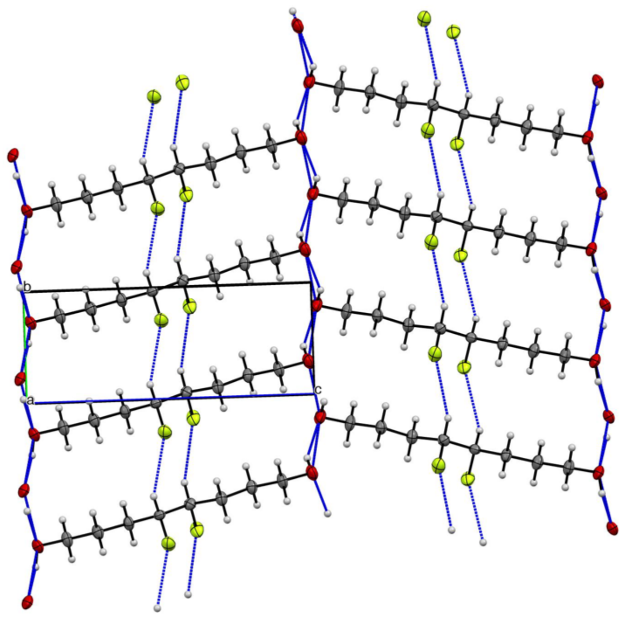

Figure 2. Crystal packing of *threo*-difluoro diol **14**. Intermolecular hydrogen bonding and short contacts are shown with blue lines.

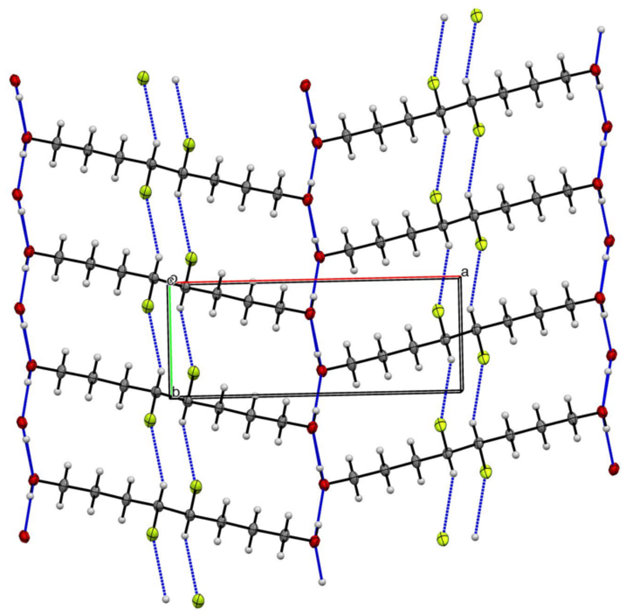

Figure 3. Crystal packing of *erythro*-difluoro diol **18**. Intermolecular hydrogen bonding and short contacts are shown with blue lines.

6. Solution-state conformations of compounds 14 and 18

Molecular modelling calculations were carried out in Gaussian 09 using a Linux computational cluster (Katana) supported by the Faculty of Science, UNSW. SMD (methanol solvent) was employed. For each compound of interest, an initial structure was built in a fully extended conformation. A set of three starting conformers was then generated by rotating the F–C–C–F bond in increments of 120°. Then, the starting conformers were subjected to Gaussian minimisation employing the M06-2X level of theory with the 6-311+G(d,p) basis set. Finally, for the low-energy conformers, NMR coupling constants were calculated using the GIAO method with B3LYP/6-311+G(d,p).

Threo-difluorinated diol 14

The conformer energies of the *threo*-difluorinated diol **14** are shown in Figure 4. This analysis suggests a relatively small amount of conformational disorder about the central F–C–C–F moiety. In both of the two significant conformers (**14a** and **14b**), the two C–F bonds align *gauche* as expected. The dominant conformer (**14a**) has an extended zigzag backbone.

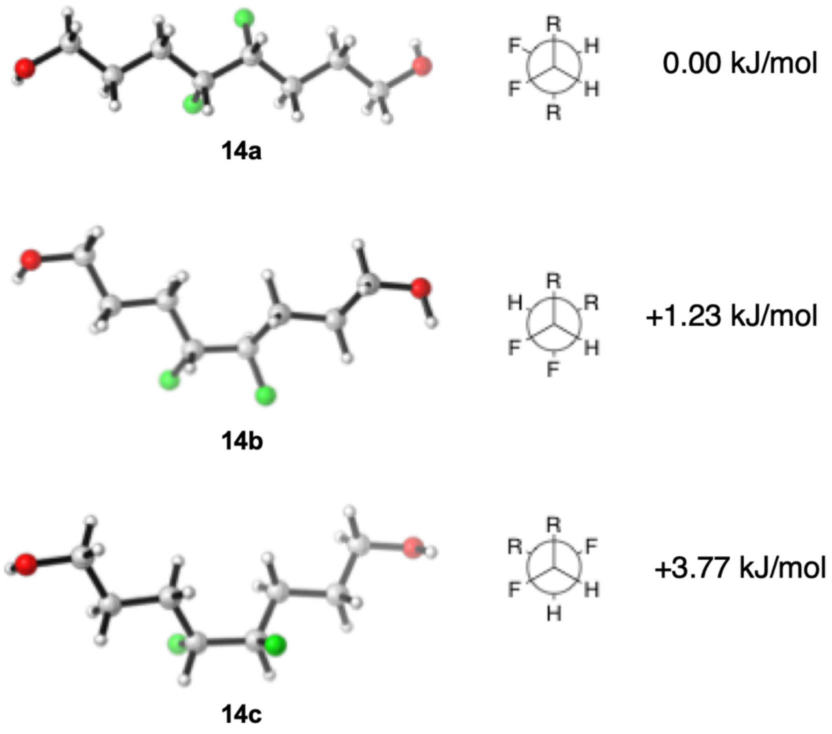

Figure 4. Conformer energies for *threo*-difluorinated diol **14**. 3D structures were generated with CYLview. R = $CH_2CH_2CH_2OH$.

Experimental NMR *J*-values for compound **14** were determined from the ^1H spectrum in methanol solution. Due to the complexity of the NMR spectrum, the *J*-values were determined through a simulation-iteration sequence using DAISY module of the Bruker TopSpin software (Table 1).

Table 1. Experimental NMR *J*-values for **14**.

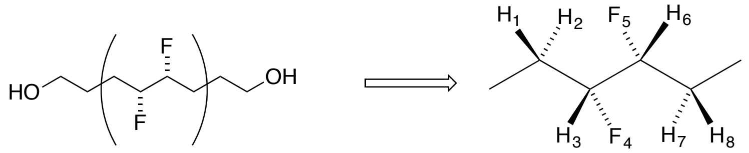

^{1}H NMR of H_3 and H_6

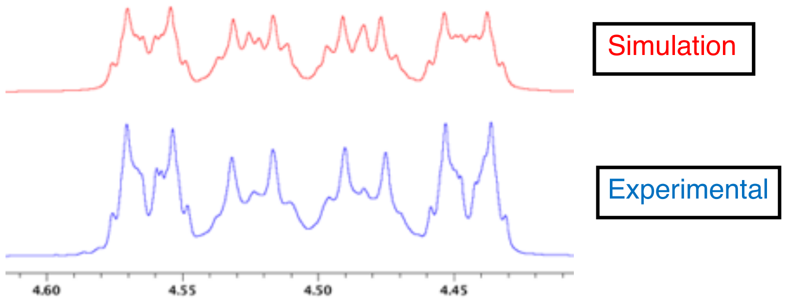

J (Hz)	H_1	H_2	H_3	F_4	F_5	H_6	H_7
H_2	14.00						
H_3	3.10	9.00					
F_4	41.30	10.42	47.00				
F_5	0.14	5.51	23.00	5.10			
H_6	0.81	0.12	3.20	23.00	47.00		
H_7	0.16	0.08	0.06	4.60	10.42	9.00	
H_8	0.12	0.20	0.86	0.34	38.00	3.10	10.0

Key $^3J_{HF}$ and $^3J_{HH}$ values of **14** that were obtained from theory *vs.* experiment were then compared. Averaging the calculated *J*-values for the two dominant conformers (**14a** and **14b**) according to a Boltzman distribution at 298 K gave a reasonable match with the experimental *J*-values (Figure 5). However the calculated $^3J_{HF}$ values are somewhat smaller than the experimental $^3J_{HF}$ values, suggesting that the extended conformer (**14a**) might actually be more dominant in solution.

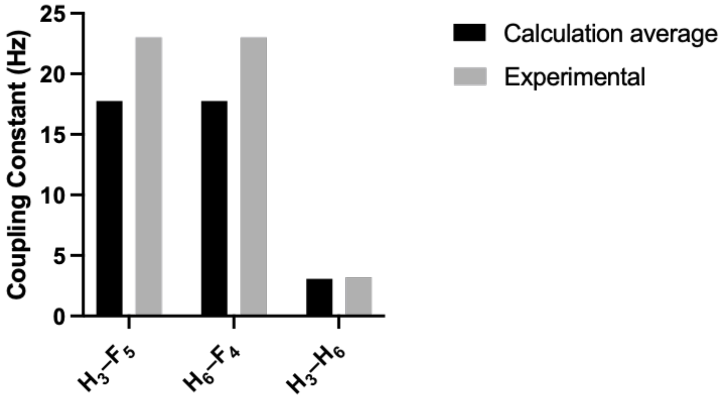

Figure 5. Comparison between calculated and experimental *J*-values of **14**.

***Erythro*-difluorinated diol 18**

The conformer energies of the *erythro*-difluorinated diol **18** are shown in Figure 6. This analysis suggests that there is significant conformational disorder about the central F–C–C–F moiety. The two lowest energy conformers (**18a** and **18b**) both have a *gauche* alignment of the two C–F bonds, while the *anti* conformer (**18c**) is only marginally higher in energy.

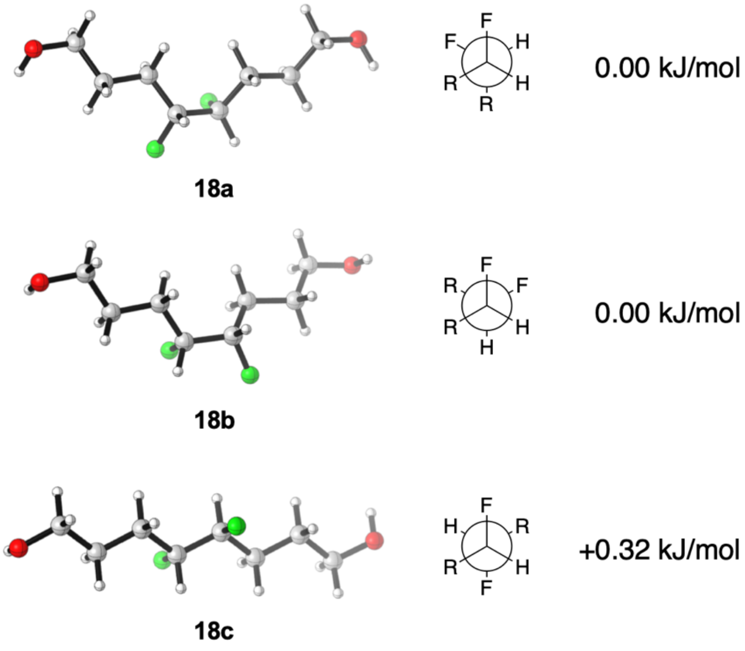

Figure 6. Conformer energies for *erythro*-difluorinated diol **18**. 3D structures were generated with CYLview. R = $CH_2CH_2CH_2OH$.

Experimental NMR *J*-values for compound **18** were determined from the ^{1}H spectrum in methanol solution. Due to the complexity of the NMR spectrum, the *J*-values were determined through a simulation-iteration sequence using DAISY module of the Bruker TopSpin software (Table 2).

Table 2. Experimental NMR *J*-values for **18**.

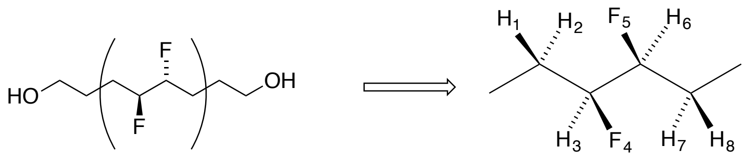

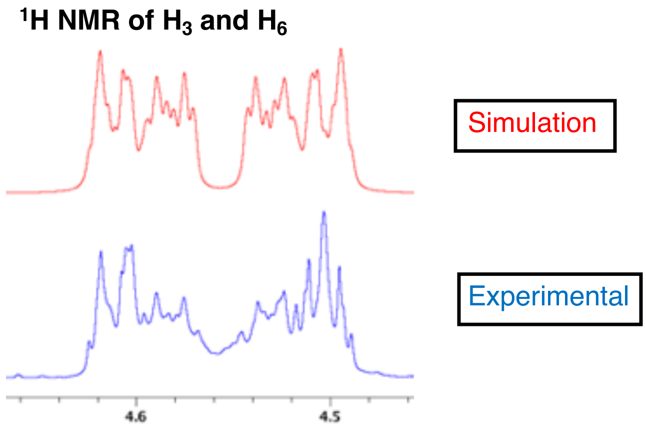

J (Hz)	H$_1$	H$_2$	H$_3$	F$_4$	F$_5$	H$_6$	H$_7$
**H$_2$**	14.10						
**H$_3$**	2.40	9.50					
**F$_4$**	39.00	8.79	48.00				
**F$_5$**	2.39	1.29	17.10	12.00			
**H$_6$**	0.18	0.20	3.60	18.40	49.10		
**H$_7$**	0.00	0.50	0.20	2.05	24.50	7.80	
**H$_8$**	0.00	0.00	0.36	2.20	49.41	2.20	12.0

Key $^3J_{HF}$ and $^3J_{HH}$ values of **18** that were obtained from theory *vs.* experiment were then compared. Averaging the calculated *J*-values for **18a**, **18b** and **18c** according to a Boltzman distribution at 298 K gave a reasonable match with the experimental *J*-values (Figure 7).

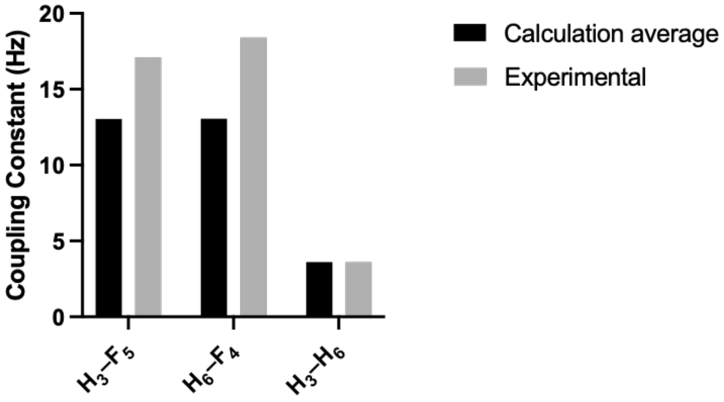

Figure 7. Comparison between calculated and experimental *J*-values of **18**.

7. HDAC inhibition assays

HDAC inhibition assays were performed by Reaction Biology Corporation on a fee-for-service basis.

Compounds **1–9** were tested in singlet 10-dose IC_{50} mode with 3-fold serial dilutions starting at 100 μM against HDAC1–11. HDAC inhibitor reference compounds Trichostatin A and TMP269 were tested in a 10-dose IC_{50} with 3-fold serial dilutions starting at 10 μM.

HDAC enzyme was added into the reaction plate, except for the control where a buffer solution consisting of Tris-HCl (50 mM, pH 8.0), NaCl (137 mM), KCl (2.7 mM), and $MgCl_2$ (1 mM) was added instead. Then, a solution of test compound in DMSO was added to the enzyme mixture. The resulting solution was spun down and preincubated. Fluorogenic HDAC substrate was then added to all reaction wells to initiate the reaction and the mixture was incubated for 2 h at 30 °C. Substrate for HDAC1,2,3,6,10: Fluorogenic peptide from p53 residues 379-382 (RHKK(Ac)AMC). Substrate for HDAC4,5,7,9, and 11: Fluorogenic HDAC Class2a Substrate (Trifluoroacetyl Lysine). Developer solution was added to generate fluorescent colour. Kinetic measurement was carried out for 1.5 h with Envision with 15 min interval (Ex/Em = 360/460 nm). Inhibitory values (Table 3) were calculated using Graphpad Prism 4 program based on a sigmoidal dose-response equation.

Table 3: IC_{50} values (μM) for compounds **1**–**9** against HDAC1–11.[a]

Compound	HDAC1	HDAC2	HDAC3	HDAC4	HDAC5	HDAC6	HDAC7	HDAC8	HDAC9	HDAC10	HDAC11
**1**	0.174	0.304	0.261	>100	19.7	0.00109	59.9	0.803	>100	0.445	62.6
**2**	0.987	1.38	0.823	-[b]	> 100	0.0736	>100	1.55	-[b]	3.88	>100
**3**	9.49	9.79	16.7	-[b]	> 100	0.704	-[b]	2.73	-[b]	27.6	-[b]
**(±)-4**	5.37	7.39	10.7	-[b]	89.6	0.0946	-[b]	4.37	-[b]	18.1	-[b]
**5**	1.24	1.12	1.71	>100	33.1	0.0411	-[b]	0.782	-[b]	0.974	-[b]
**6**	10.9	12.4	21.7	-[b]	53.4	0.194	-[b]	1.72	-[b]	30.3	-[b]
**7**	0.135	0.403	0.187	10.6	3.36	0.00187	8.33	0.433	19.4	0.540	2.61
**8**	9.69	16.4	10.7	>100	81.0	0.272	91.7	13.1	>100	27.3	44.1
**9**	13.3	19.4	18.7	>100	> 100	0.375	>100	11.0	-[b]	36.7	18.3

[a] The blank (DMSO) value was entered as 1.00×10^{-12} of concentration for curve fitting.
[b] No inhibition.

An alternative representation of the pIC_{50} data is shown below, where the data is grouped by compound (instead of by HDAC target as in the main manuscript). This alternative representation shows that the selectivity between HDAC3 *vs.* HDAC8 changes in going from one compound to another.

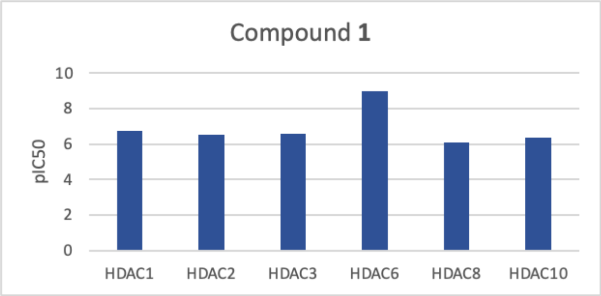

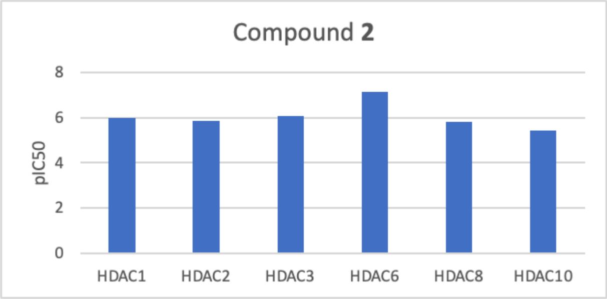

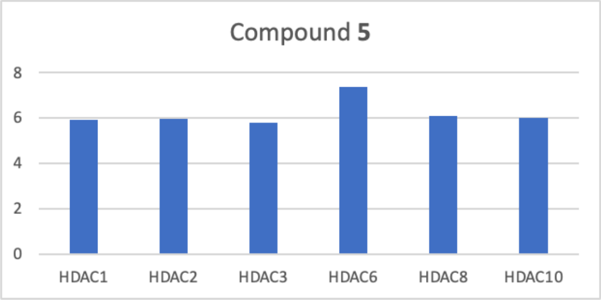

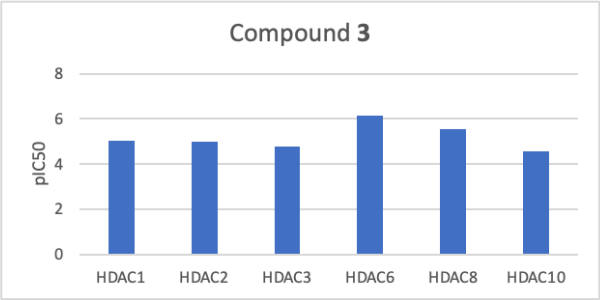

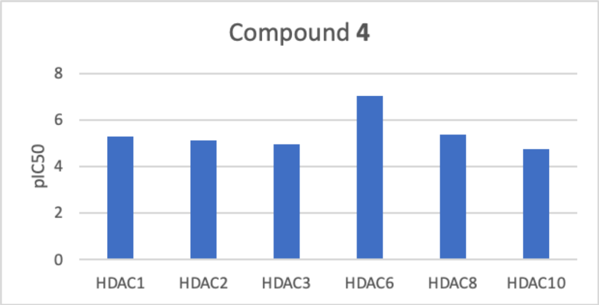

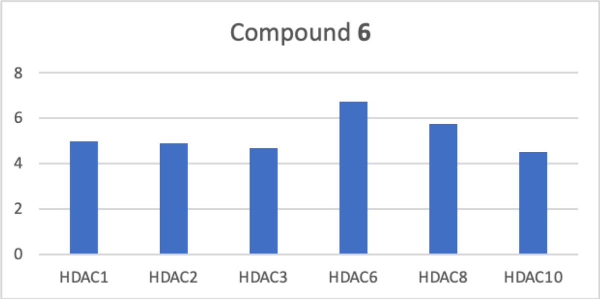

8. Docking studies

Programs used: Discovery Studio Client v17.2.0.16349 (Dassault Systèmes Biovia Corp, San Diego, CA, USA); Gold Version 5.3.0 (Cambridge Crystallographic Data Centre).

General minimization procedure: Energy minimisations were performed in Discovery Studio after applying the CHARMm force field. Default settings were used except the maximum steps was changed to 10,000. Minimizations were considered converged with gradient tolerance (0.1000000 kcal/mol $Å^{-1}$) satisfied.

Crystal structure preparation: Crystal structures were prepared by first downloading the pdb file from the Protein Data Bank and deleting the bonds associated with the metal cation. The corrected file was then imported into DS and water molecules were removed and hydrogens added to account for any missing in the crystal structure. A three-part minimisation was performed with different components of the crystal structure constrained at each part. The first minimisation was performed on the hydrogens with all atoms fixed excluding hydrogen. The second was performed on the side chains with the backbone and bound ligand fixed. The final minimisation was performed on all amino acids outside of the active site, where the active site is a defined sphere around the ligand that encompasses the interacting amino acid residues. The ligand was then removed from the structure and the receptor was ready for docking.

HDAC2 docking: Docking was performed using GOLD docking software through Discovery Studio. The ligands docked were first minimized following *General minimization procedure.* Structures were docked into the crystal structure 4LXZ (PDB ID) using the fitness function ChemPLP and the default settings were used except the number of dockings was changed to 100, detect cavity and early termination were turned off and the flexibility parameters 'flip amide bonds' and 'flipping pyramidal nitrogens' were set to true. The 100 poses were clustered, and the top pose in the largest cluster at 2 Å was selected as the final result. Docking scores are displayed in the Table below. The best-performing scoring function was "ChemPLP fitness," which correctly predicted that the non-fluorinated lead compounds (*i.e.* **1**, **7**) would be more potent than the fluorinated analogs (*i.e.* **2**–**4**, **8**–**9**). However, even this scoring function did not successfully predict the rank order of potency within each series of fluorinated analogs.

Compound	“ChemPLP fitness” (higher = better)	“ChemPLP score” (lower = better)
**1**	85.88	–63.45
**2**	78.64	–62.00
**3**	83.19	–65.01
**4**	79.07	–66.29
**7**	83.89	–67.55
**8**	64.40	–53.61
**9**	73.42	–58.80

9. References

[1] Donohoe, T.J.; Lipiński, R.M. Interplay of Cascade Oxidative Cyclization and Hydride Shifts in the Synthesis of the ABC Spiroketal Ring System of Pectenotoxin-4. *Angew. Chem. Int. Ed.* **2013**, *52*, 2491–2494.

[2] Musilek, K.; Holas, O.; Kuca, K.; Jun, D.; Dohnal, V.; Opletalova, V.; Dolezal, M. Novel series of bispyridinium compounds bearing a (*Z*)-but-2-ene linker – Synthesis and evaluation of their reactivation activity against tabun and paraoxon-inhibited acetylcholinesterase. *Bioorg. Med. Chem. Lett.* **2007**, *17*, 3172–3176.

[3] Olszewska, B.; Kryczka, B.; Zawisza, A. Asymmetric Synthesis of Optically Active 2-Vinylpyrrolidines and 2-Vinylpiperidines by Palladium-Catalysed Cyclisation of Amino Allylic Carbonates. *Tetrahedron Lett.* **2012**, *53*, 6826–6829.
